# Hypervalent Iodine-Mediated Chemoselective Bromination of Terminal Alkynes

**DOI:** 10.3389/fchem.2022.879789

**Published:** 2022-04-07

**Authors:** Youzhi Li, Xuemei Chen, Daya Huang, Zhenming Xie, Yan Liu

**Affiliations:** ^1^ School of Chemical Engineering and Light Industry, Guangdong University of Technology, Guangzhou, China; ^2^ Guangdong Provincial Key Laboratory of Plant Resources Biorefinery, Guangdong University of Technology, Guangzhou, China

**Keywords:** bromination, hypervalent iodine reagent, 1-bromoalkynes, 1,2-dibromoalkene, α,α-dibromoketone, tetrabromoalkanes, chemoselectivity, alkyne

## Abstract

Practical approaches for chemoselective mono-bromination, di-bromination, and tetra-bromination of terminal alkynes to generate 1-bromoalkynes, 1,2-dibromoalkenes, α,α-dibromoketones, and 1,1,2,2-tetrabromoalkanes based on efficient oxidative brominations mediated by a hypervalent iodine reagent have been developed. Chemoselective bromination can be realized under mild conditions by altering the bromine source. The tetrabutylammonium bromide (TBAB)/(diacetoxyiodo)benzene (PIDA) system is specific for mono-bromination to provide 1-bromoalkynes, while the NaBr/PIDA system is selective toward di-bromination to achieve 1,2-dibromoalkenes. When a certain amount of water was added to the NaBr/PIDA system, a different di-bromination product, α,α-dibromo ketones, was generated. Tetra-bromination of terminal alkynes provides an efficient protocol for the synthesis of 1,1,2,2-tetrabromoalkanes in a system with an excess loading of NaBr/PIDA in one pot. This bromination affords good yields (up to 99%) with excellent chemoselectivity (up to 100%). These methods can be applied to the efficient chemoselective synthesis of bromide derivatives, intermediates, and related biologically active compounds.

## Introduction

Halogenated organic compounds play an important role in organic synthesis as synthetic intermediates or key synthetic precursors due to their feasible transformation into a variety of functional compounds for material science, bioactive compounds, or industrial chemicals (Chen et al., 2010; Adimurthym et al., 2013; Wu et al., 2017). For example, monohaloalkynes and α,β-dihaloalkenes have found a variety of application as dual functionalized molecules (Brand and Waser, 2012; Shi and Lei, 2014; Wu and Jang, 2014; Shi, 2015), and α,α-dihaloketones are important building blocks in the synthesis of heterocyclic compounds, unsaturated acids and acetylenic alcohols, and cyclopropanation (Furukawa et al., 1976; Kawabata et al., 1977; Kowalski and Fields, 1982; Zhdankin and Stang, 1993; SooáKim et al., 1995; Madabhushi et al., 2013; Sadhukhan et al., 2020). In addition, α,α-dihaloketones and tetrahalides have been reported to be potent antitumor, antibacterial, antifungal, antineoplastic, antiviral, and antioxidizing agents (Butler and Walker, 1993; Bora et al., 2000; Khazaei et al., 2010). Various synthetic methods for halogenated products have been developed in the past decades by utilizing Cl_2_/AsF_5_ or XeF + MF_6_^−^ (Olah et al., 1998), hydrogen halide (De la Mare and Bolton, 1982; T Becker, 2001), molecular bromine (Chiappe et al., 2001; Ryu et al., 2002), dichlorosulfoxide and m-chloroperoxybenzoic acid (MCPBA) and BCl_3_ (Larock, 1999), or *N*-bromosuccinimide (Dalton et al., 1968) as halogen source. Among them, oxidative halogenations mediated by hypervalent iodine compounds (Braddock et al., 2004; Liu et al., 2017), which have the advantages of low toxicity, readily available reagents, mild conditions, excellent selectivity, and a comparable reactivity to alternative methods, have recently attracted considerable attention.

We recently reported our investigation of the oxidative iodination of alkynes mediated by the hypervalent iodine reagent (diacetoxyiodo)benzene (PIDA) (Liu et al., 2017). The chemoselective mono-iodination, di-iodination, and tri-iodination of alkynes were efficiently achieved in good yield with excellent chemoselectivity by altering the iodine source. The system based on hypervalent iodine, i.e., the tetrabutylammonium iodide (TBAI)/PIDA system, is specific for monoiodination, and the KI/PIDA system results in di-iodination. Combining the TBAI/PIDA and KI/PIDA systems in one pot provided the corresponding tri-iodination products efficiently.

Inspired by these observations, and as part of our ongoing interest in constructing practical methods for selective halogenation reactions, we have become keenly interested in the possibility of chemoselective bromination of alkynes mediated by hypervalent iodine reagents. In this context, we wish to report our initial study on the mono-bromination, di-bromination, or tetra-bromination of alkynes to construct 1-bromoalkynes, 1,2-dibromoalkenes, α,α-dibromoketones, and 1,1,2,2-tetrabromoalkanes, respectively, based on a highly chemoselective oxidative bromination method mediated by hypervalent iodine reagents.

## Results and Discussion

To investigate the oxidative bromination of alkynes mediated by PIDA, we initially studied the influence of the bromine source, tetrabutylammonium bromide (TBAB), KBr, NaBr, by using *p*-tolylethyne as a mode substrate. Acetonitrile (CH_3_CN) or CH_3_CN/H_2_O solvent system was employed in our present bromination study, since these solvents system are optimized for high chemoselective iodination of alkynes mediated by hypervalent iodine reagents in our previous research (Liu et al., 2017). According to our previous results, tetrabutylammonium iodide (TBAI) are specific for the mono-iodination product. Similarly, 1-bromoalkyne **2a** (the mono-bromination products of alkyne) was observed as the major product in 76% yield when TBAB was used as the bromine source, under the reaction conditions specified in Table 1, the molar ratio of **2a** to **3a** to **4a** to **5a** to **6a** is 97:1.5:1.5:0:0 (entry 1), indicating good selectivity toward **2a**. Increasing the loading of PIDA (2.0 equiv) is favorable and more efficient for mono-bromination of alkyne, 89% yield of **2a** was observed and the molar ratio of **2a** to **3a** to **4a** to **5a** to **6a** is 94:0:0:6:0 (entry 2). Interestingly, good selectivity toward **2a** can still be observed when changing the bromine source to KBr, albeit the yield of **2a** was remarkably decrease (entry 3).

**TABLE 1:** Bromination of *p*-tolylethyne with hypervalent iodine reagent.

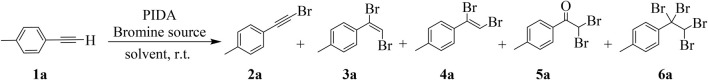							
Entry	(O) (equiv)	Bromine source (equiv)	Solvent	Temp. (°C)	t (h)	Major product	% Yield (2a:3a:4a:5a:6a)^a^
1	PIDA (1.0)	TBAB (1.2)	CH_3_CN	r.t	24	2a	76^b^ (97:1.5:1.5:0:0)
2	PIDA (2.0)	TBAB (1.2)	CH_3_CN	r.t	3	2a	89^c^ (94:0:0:6:0)
3	PIDA (1.0)	KBr (2.5)	CH_3_CN	r.t	24	2a	44 (87.2:12.8:0:0:0)
4	PIDA (1.0)	NaBr (2.5)	CH_3_CN	r.t	24	3a/4a	66/6 (5.1:84.6:10.3:0:0)
5	PIDA (2.0)	NaBr (2.5)	CH_3_CN	r.t	3	3a/4a	62/28 (0:62:28:10:0)
6	PIDA (1.0)	NaBr (4.0)	CH_3_CN	r.t	5	3a/4a	77^d^/8 (0:79.4:8.2:7.2:5.2)
7	PIDA (1.0)	KBr (2.5)	CH_3_CN/H_2_O (1/3)	r.t	24	5a	48 (1.6:18:3.3:77.1:0)
8	PIDA (1.0)	NaBr (2.5)	CH_3_CN/H_2_O (1/3)	r.t	24	5a	42 (0:13.7:3.9:82.3:0)
9	PIDA (2.0)	NaBr (2.5)	CH_3_CN/H_2_O (1/3)	r.t	10	5a	82 (0:2.9:13.5:83.6:0)
10	PIDA (3.0)	NaBr (3.0)	CH_3_CN/H_2_O (1/3)	r.t	10	5a	97 (0:0:0:97:3)

a The yield and the molar ratio of **2a** to **3a** to **4a** to **5a** to **6a** were determined via 1H NMR, spectroscopy using acetophenone as the internal standard.

b The isolated yield of **2a** in entry 1 was 74%.

c The isolated yield of **2a** in entry 2 was 84%.

d The isolated yield of **3a** in entry 6 was 74%.

Interestingly, changing the bromine source from KBr to NaBr dramatically changed the chemoselectivity to give the di-bromination product of the alkyne, **3a** (E-type 1,2-dibromoalkene), as the major product in 66% yield, and **4a** (Z-type 1,2-dibromoalkene) as the minor product in 6% yield; the **2a**:**3a**:**4a**:**5a**:**6a** ratio is 5.1:84.6:10.3:0:0 (entry 4). Increasing the loading of PIDA or NaBr can efficiently promote the selectivity of the transformation towards 1,2-dibromoalkene, as observed from the resulting total di-bromination product yields of 90% (entry 5) and 85% (entry 6), respectively.

With these promising results in hand, we further investigated the influence of the solvent on the chemoselectivity of the bromination. In our previous study of the hypervalent iodine-reagent mediated di-iodination of alkynes to construct 1,2-diiodoalkenes, use of the KI/PIDA system in CH_3_CN/H_2_O (1:3 v/v) was crucial to achieve this chemoselectivity (Liu et al., 2017). In the present study, however, when the halogen source was changed from KI to KBr, the expected selectivity towards the di-bromination product (1,2-tribromoalkene) was not observed. Interestingly, α,α-dibromoketone **5a** was obtained instead in 48% yield with good chemoselectivity; the **2a**:**3a**:**4a**:**5a**:**6a** ratio was 1.6:18:3.3:77.1:0 (entry 7). Further changing the bromine source to NaBr provided **5a** with higher selectivity (entry 8). Furthermore, increasing the loading of PIDA (2 equiv) favorably and strongly influenced the transformation towards **5a** in 82% yield without loss of chemoselectivity (entry 9). A further increased loading of PIDA (3 equiv) afforded **5a** almost exclusively, giving a **2a**:**3a**:**4a**:**5a**:**6a** ratio of 0:0:0:97:3 (entry 10). According to the literatures (Madabhushi et al., 2013; Wu et al., 2017), it’s highly likely that the residual water in solvent participated in the generation of HBrO, which subsequently promoted the generation α,α-dibromoketones under our reaction conditions.

Encouraged by these favorable results, we became interested in the possibility of the direct transformation of **1a** into tetra-bromination product **6a** mediated by hypervalent iodine reagents. It is worth mentioning that the construction of tetrahalides in good yield and selectivity remains challenging. In our system, the tetra-bromination product (1,1,2,2-tetrabromoalkanes) may be derived from the *in-situ* generated 1,2-dibromoalkene through addition reaction. To realize the construction of the target molecule, we initially tried increasing the loading of both PIDA (2 equiv) and NaBr (4 equiv). Fortunately, the tetra-bromination product **6a** was obtained in 54% yield; the **2a**:**3a**:**4a**:**5a**:**6a** ratio was 0:30.2:15.9:0:53.9 (Table 2, entry 1). To consume the intermediate 1,2-dibromoalkene, we tried using a higher loading of both PIDA (3 equiv) and NaBr (6 equiv). We were delighted to obtain a good yield of 74% and high chemoselectivity toward **6a**, with a **2a**:**3a**:**4a**:**5a**:**6a** ratio of 3.8:5.9:3.5:0:86.8 (entry 2). Based on the results from entries 1 and 2, we studied the influence of loading PIDA/NaBr in two batches (in one pot). The result showed that the 1,2-dibromoalkene was fully consumed under the two-batch reaction conditions to afford **6a** in good yield and excellent chemoselectivity; the **2a**:**3a**:**4a**:**5a**:**6a** ratio was 0:0:0:9.2:90.8 (entry 3). Further increasing the amounts of both PIDA and NaBr resulted in this transformation almost exclusively, with 93% yield, high chemoselectivity, and a **2a**:**3a**:**4a**:**5a**:**6a** ratio of 0:0:0:6.7:93.3 (entry 4).

**TABLE 2:** One-Pot Bromination of *p*-Tolylethyne with Hypervalent iodine Reagent.

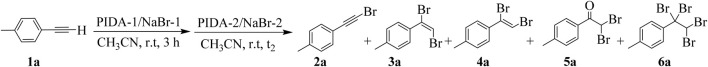						
Entry	PIDA-1 (equiv)	NaBr-1 (equiv)	PIAD-2 (equiv)	NaBr-2 (equiv)	t_2_ (h)	% Yield (2a:3a:4a:5a:6a)^a^
1	2.0	4.0	—	—	0	54 (0:30.2:15.9:0:53.9)
2	3.0	6.0	—	—	0	74 (3.8:5.9:3.5:0:86.8)
3	2.0	4.0	2.0	4.0	3	80 (0:0:0:9.2:90.8)
4	4.0	6.0	1.0	3.0	3	93 (0:0:0:6.7:93.3)

a The yield and the molar ratio of **2a** to **3a** to **4a** to **5a** to **6a** were determined via ^1^H NMR, spectroscopy using acetophenone as the internal standard.

Therefore, the optimal conditions for the synthesis of **2a**, **3a**, **4a**, **5a** or **6a** were established as follows: 1) reaction of the alkyne (1.0 equiv) with TBAB (1.2 equiv) and PIDA (2.0 equiv) in CH_3_CN at room temperature for 1–3 h affords **2a** (method **A**); 2) reaction of the alkyne (1.0 equiv) with NaBr (4.0 equiv) and PIDA (1.0 equiv) in CH_3_CN at room temperature for 1–5 h provides **3a**/**4a** (method **B**); 3) reaction of the alkyne (1.0 equiv) with NaBr (3.0 equiv) and PIDA (3.0 equiv) in CH_3_CN/H_2_O (1:3, v/v) at room temperature for 10 h provides **5a** (method **C**); 4) reaction of the alkyne (1.0 equiv) with NaBr (6.0 equiv) and PIDA (4.0 equiv) in CH_3_CN at room temperature for 3 h, after that, NaBr (3.0 equiv) and PIDA (1.0 equiv) were then added and stirred for 3 h provides **6a** (method **D**); Having established practical methods for the chemoselective mono-bromination, di-bromination, and tri-bromination of *p*-tolylethyne mediated by hypervalent iodine reagents to furnish 1-bromoalkynes, 1,2-dibromoalkene, α,α-dibromoketone and 1.1,2,2-tetrabromoalkanes, respectively, we next examined the generality and selectivity of the bromination of terminal alkynes (Table 3).

**TABLE 3:** Selective bromination of terminal alkynes with hypervalent iodine reagents.

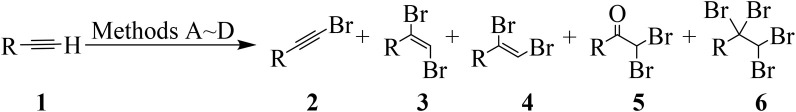				
Entry	Alkyne	Method^a^	Major product	% Yield (2a:3a:4a:5a:6a)^b^
1	R = *p*-to1yl	A	2a	89 (94:0:0:6:0)
2		B	3a/4a	77/8 (0:83.7:8.7:7.6:0)
3		C	5a	97 (0:0:0:96.6:3.4)
4		D	6a	93 (0:0:0:6.4:93.6)
5	R = *m*-to1yl	A	2b	95 (100:0:0:0:0)
6		B	3b/4b	58/3 (0:87.9:4.5:7.6:0)
7		C	5b	73 (0:0:0:81.1:18.9)
8		D	6b	64 (0:0:0:0:100)
9	R = Ph	A	2c	84 (100:0:0:0:0)
10		B	3c/4c	45/3 (17.4:52.4:3.5:17.4:9.3)
11		C	5c	72 (0:0:0:92.3:7.7)
12		D	6c	64 (0:0:0:0:100)
13	R = *p*-MeO-C_6_H_4_	A	2d	69 (75.8:24.2:0:0:0)^c^
14		B	3d/4d	82/3 (10.5:86.3:3.2:0:0)^d^
15		C	5d	87 (0:0:0:100:0)
16		D	6d	trace
17	R = *p*-MeOCO-C_6_H_4_	A	2e	88 (100:0:0:0:0)
18		B	3e/4e	56/8 (20:70:10:0:0)
19		C	5e	9 (0:8.3:53.6:10.7:27.4)
20		D	6e	15 (0:64.9:14.9:0:20.2)
21	R = *p*-F-C_6_H_4_	A	2f	98 (100:0:0:0:0)
22		B	3f/4f	55/6 (0:79.7:8.7:0:11.6)
23		C	5f	78 (0:3.1:0:80.4:16.5)
24		D	6f	53 (0:0:0:8.6:91.4)
25	R = *p*-Cl-C_6_H_4_	A	2g	99 (100:0:0:0:0)
26		B	3g/4g	40/4 (49.4:46:4.6:0:0)
27		C	5g	69 (0:0:0:82.1:17.9)
28		D	6g	31 (0:12.1:34.5:0:53.4)
29	R = *p*-Br-C_6_H_4_	A	2h	78 (100:0:0:0:0)
30		B	3h/4h	42/4 (14.8:77.8:7.4:0:0)
31		C	5h	54 (21.3:4.3:0:57.4:17)
32		D	6h	22 (32.2:6.2:20.8:0:33.8)
33	R = *p*-CF_3_-C_6_H_4_	A	2i	99 (100:0:0:0:0)
34		B	3i/4i	54/11 (16.7:69.2:14.1:0:0)
35		C	5i	9 (0:11.1:65.4:11.1:12.4)
36		D	6i	14 (0:12.2:71.5:2:14.3)
37	R = *p*-N O _2_-C_6_H_4_	A	2j	85 (97.7:2.3:0:0:0)
38		B	3j/4j	39/10 (7.5:73.6:18.9:0:0)
39		C	5j	2 (0:7.5:62.7:3:26.8)
40		D	6j	18 (53.8:12.5:15.4:0:18.3)
41	R = CH_2_CH_2_OH	A	2k	56 (100:0:0:0:0)
42		B	3k/4k	trace
43		C	5k	trace
44		D	6k	61 (0:10.4:2.6:7.8:79.2)

a Method **A**: Alkyne (1.0 equiv), TBAB (1.2 equiv), and PIDA (2.0 equiv) in CH_3_CN, r. t, 1–3 h. Method **B**: Alkyne (1.0 equiv), NaBr (4.0 equiv), and PIDA (1.0 equiv) in CH_3_CN, r. t, 1–5 h. Method C: Alkyne (1.0 equiv), NaBr (3.0 equiv), and PIDA (3.0 equiv) in CH_3_CN/H_2_O (1/3), r. t, 10 h. Method **D**: (i) Alkyne (1.0 equiv), NaBr (6.0 equiv), and PIDA (4.0 equiv) in CH_3_CN, r. t, 3 h; (ii) NaBr (3.0 equiv), and PIDA (1.0 equiv), r. t, 3 h.

b The yield and the molar ratio of **2a** to **3a** to **4a** to **5a** to **6a** were determined via ^1^H NMR, spectroscopy using acetophenone as the internal standard.

c The isolated yield of **2d**ays in entry 13 was 73%.

d The isolated yield of **3d**ays in entry 14 was 86%.

We initially investigated the chemoselective bromination of various types of terminal aromatic alkynes. Our results showed that the mono-bromination of various terminal aromatic alkynes using method A afforded 1-bromoalkynes in good to high yields (69–99%) with excellent chemoselectivity (up to 100% in most cases). The substituents on the aromatic ring of the alkynes did not significantly influence the selectivity (entries 1, 5, 9, 13, 17, 21, 25, 29, 33, and 37). In the di-bromination of various terminal aromatic alkynes using method B, the desired 1,2-dibromoalkenes were obtained in moderate to good yields with the trans-configuration (*E*-type) products as the major products. Electron-donating groups were tolerated under the applied reaction conditions (entries 2, 6 and 14). However, electron-withdrawing groups resulted in some loss of both yield and selectivity (entries 18, 22, 26, 30, 34, and 38).

Interestingly, the hypervalent iodine-reagent mediated di-bromination of terminal alkynes to furnish α,α-dibromoketone **5** was noticeably affected by the substituents on the aromatic ring of the alkynes. Under the reaction conditions in method C, electron-donating groups were favorable for the synthesis of the corresponding products in good to high yield (72–97%) with very high chemoselectivity (entries 3, 7 and 15). However, halogen substituents on the aromatic ring of the alkynes slightly decreased both the yield and selectivity (entries 23, 27, and 31). The yield and the selectivity decreased dramatically when strongly electron-withdrawing groups were introduced, and the cis-configuration (*Z*-type) 1,2-dibromoalkenes were unexpectedly obtained as the major products (entries 19, 35, and 39).

With these results in hand, we studied the tetra-bromination of terminal aromatic alkynes utilizing the one-pot system described in Table 2. Substrates with weakly electron-donating groups smoothly furnished the corresponding tetra-bromination products (1,1,2,2-tetrabromoalkanes **6**) in up to 93% yield with high chemoselectivity (entries 4, 8, and 12). In contrast, the use of alkynes containing halogen groups resulted in a significant loss of both yield and selectivity (entries 24, 28, and 32). Unfortunately, alkynes with strongly electron-donating or electron-withdrawing groups were unfavorable for the tetra-bromination. For example, only 14 and 18% yields were observed when a trifluoromethyl group (CF_3_) and nitro group (NO_2_) were introduced, respectively (entries 36 and 40). The corresponding product was not obtained when a methoxy group (MeO) was introduced, instead, the α,α-dibromoketone was unexpectedly obtained as the major product with high selectivity (entry 16).

In our previous study, terminal aliphatic alkynes were tolerated in the hypervalent iodine-reagent mediated iodination. Therefore, we were also interested in comparing the bromination of terminal aliphatic alkynes with that of terminal aromatic alkynes. Under the applied reaction conditions, an aliphatic terminal alkyne with a hydroxyl group was suitable for mono-bromination, giving 56% yield and high chemoselectivity (entry 41), and for tetra-bromination in a similar yield (entry 44). However, the di-bromination conditions failed to provide either 1,2-dibromoalkenes or α,α-dibromoketone (entries 42 and 43).

## Conclusion

In summary, we have demonstrated a practical chemoselective approach for the mono-bromination, di-bromination, or tetra-bromination of terminal alkynes to furnish 1-bromoalkynes, 1,2-dibromoalkenes, α,α-dibromoketones, and 1.1,2,2-tetrabromoalkanes based on efficient oxidative bromination mediated by hypervalent iodine reagents. Practically, chemoselective bromination can be realized under mild conditions by suitable choice of the bromine source. The TBAB/PIDA system, which is specific toward the mono-bromination to provide 1-bromoalkynes, is favorable for both aromatic and aliphatic alkynes. The NaBr/PIDA system is selective toward di-bromination to achieve 1,2-dibromoalkenes from aromatic alkynes. When a certain amount of water was added to the NaBr/PIDA system, a different di-bromination product, α,α-dibromoketones, were successfully generated. Tetra-bromination of terminal alkynes by using the NaBr/PIDA system with an excess loading provided an efficient protocol for the synthesis of 1,1,2,2-tetrabromoalkanes in one pot. Electron-donating groups on the aromatic alkynes showed a positive effect on the chemoselectivity towards α,α-dibromoketones and 1,1,2,2-tetrabromoalkanes, while electron-withdrawing groups had a negative influence. This bromination affords good yields (up to 99%) and proceeds highly selectively (up to 100%) and can thus be applied to the efficient chemoselective synthesis of bromide derivatives, intermediates, and related biologically active compounds.

## Materials and Methods

### General

^1^H NMR and ^13^C{^1^H} NMR spectra were recorded on a Bruker AVANCE III 400 MHz or 500 MHz spectrometer (400 MHz or 500 MHz for ^1^H NMR, 100 MHz or 125 MHz for ^13^C NMR). Tetramethylsilane (TMS) was used as an internal standard (0 ppm) for the ^1^H NMR spectra, and CDCl_3_ was used as the internal standard (77.0 ppm) for the ^13^C{^1^H} NMR spectra. Reactions were monitored by thin-layer chromatography (TLC). Reaction products were purified by column chromatography on silica gel (300–400 mesh). Chemical reagents were purchased from common commercial suppliers and used as received.

General Procedures for the Bromination of Alkynes. **Method A**. PIDA (193.2 mg, 0.6 mmol) was added in portions over a period of 15 min to a mixture of the alkyne (0.3 mmol) and TBAB (133.0 mg, 0.36 mmol) in CH_3_CN (3 ml), and the reaction mixture was stirred at room temperature for 1–3 h. The reaction progress was monitored by TLC. Upon completion, the reaction mixture was quenched with saturated aqueous Na_2_S_2_O_3_, washed with brine, extracted with ethyl acetate, and dried over anhydrous Na_2_SO_4_. After filtration, the solvent was removed under reduced pressure to afford the crude product, which was purified by column chromatography using hexane or hexane/ethyl acetate and analyzed by ^1^H and ^13^C NMR spectroscopy.

**Method B**. PIDA (96.6 mg, 0.3 mmol) was added in portions over a period of 15 min to a mixture of the alkyne (0.3 mmol) and NaBr (123.5 mg, 1.2 mmol) in CH_3_CN (3 ml), and the reaction mixture was stirred at room temperature for 1–5 h. The reaction progress was monitored by TLC. Upon completion, the reaction mixture was quenched with saturated aqueous Na_2_S_2_O_3_, washed with brine, extracted with ethyl acetate, and dried over anhydrous Na_2_SO_4_. After filtration, the solvent was removed under reduced pressure to afford the crude product, which was purified by column chromatography using hexane or hexane/ethyl acetate and analyzed by ^1^H and ^13^C NMR spectroscopy.

**Method C**. PIDA (289.8 mg, 0.9 mmol) was added in portions over a period of 15 min to a mixture of the alkyne (0.3 mmol) and NaBr (92.6 mg, 0.9 mmol) in CH_3_CN (1 ml) and H_2_O (3 ml), and the reaction mixture was stirred at room temperature for 10 h. The reaction progress was monitored by TLC. Upon completion, the reaction mixture was quenched with saturated aqueous Na_2_S_2_O_3_, washed with brine, extracted with ethyl acetate, and dried over anhydrous Na_2_SO_4_. After filtration, the solvent was removed under reduced pressure to afford the crude product, which was purified by column chromatography using hexane or hexane/ethyl acetate and analyzed by ^1^H and ^13^C NMR spectroscopy.

**Method D**. PIDA (386.4 mg, 1.2 mmol) was added in portions over a period of 15 min to a mixture of the alkyne (0.3 mmol) and NaBr (185.2.0 mg, 1.8 mmol) in CH_3_CN (3 ml). After stirring at room temperature for 3 h, NaBr (92.6 mg, 0.9 mmol) were added. More PIDA (96.6 mg, 0.3 mmol) was added in portions over a period of 15 min, and the reaction mixture was stirred at room temperature for 3 h. The reaction progress was monitored by TLC. Upon completion, the reaction mixture was quenched with saturated aqueous Na_2_S_2_O_3_, washed with brine, extracted with ethyl acetate, and dried over anhydrous Na_2_SO_4_. After filtration, the solvent was removed under reduced pressure to afford the crude product, which was purified by column chromatography using hexane or hexane/ethyl acetate and analyzed by ^1^H and ^13^C NMR spectroscopy.

**1-(bromoethynyl)-4-methylbenzene (2a)**: The crude product was purified by silica gel column chromatography with hexanes as the eluent to give a yellow oil (52.1 mg, 89%). ^1^H NMR (400 MHz, CDCl_3_): *δ* = 7.33 (d, *J* = 7.6 Hz, 2H), 7.11 (d, *J* = 8.0 Hz, 2H), 2.34 ppm (s, 3H); ^13^C{^1^H} NMR (100 MHz, CDCl_3_): *δ* = 138.9, 131.9, 129.2, 119.7, 80.2, 48.9, 21.6 ppm.

**(*E/Z*)-1-(1,2-dibromovinyl)-4-methylbenzene (3a/4a)**: The crude product was purified by silica gel column chromatography with hexanes as the eluent to give **3a** as a yellow oil (63.8 mg, 77%) and **4a** as yellow oil (6.6 mg, 8%), respectively. **3a**: ^1^H NMR (400 MHz, CDCl_3_): *δ* = 7.41 (d, *J* = 8.0 Hz, 2H), 7.19 (d, *J* = 7.6 Hz, 2H), 6.76 (s, ^1^H), 2.37 ppm (s, 3H); ^13^C{^1^H} NMR (100 MHz, CDCl_3_): *δ* = 139.6, 134.1, 129.1, 129.0, 121.6, 102.5, 21.5 ppm **4a**: ^1^H NMR (400 MHz, CDCl_3_): *δ* = 7.39 (d, *J* = 8.4 Hz, 2H), 7.15 (d, *J* = 8.0 Hz, 2H), 7.00 (s, ^1^H), 2.36 ppm (s, 3H); ^13^C{^1^H} NMR (100 MHz, CDCl_3_): *δ* = 139.6, 135.7, 131.3, 129.3, 127.6, 108.0, 21.3 ppm.

**2,2-dibromo-1-(p-tolyl)ethanone (5a)**: The crude product was purified by silica gel column chromatography with 50:1 hexanes/EtOAc as the eluent to give a yellow oil (85 mg, 97%). ^1^H NMR (400 MHz, CDCl_3_): *δ* = 7.97 (d, *J* = 8.0 Hz, 2H), 7.29 (d, *J* = 8.4 Hz, 2H), 6.74 (s, ^1^H), 2.43 ppm (s, 3H); ^13^C{^1^H} NMR (100 MHz, CDCl_3_): *δ* = 185.7, 145.8, 129.8, 129.7, 128.2, 40.2, 21.9 ppm.

**1-methyl-4-(1,1,2,2-tetrabromoethyl)benzene (6a)**: The crude product was purified by silica gel column chromatography with 100:1 hexanes/EtOAc as the eluent to give a yellow oil (121.6 mg, 93%). ^1^H NMR (400 MHz, CDCl_3_): *δ* = 7.74 (d, *J* = 8.0 Hz, 2H), 7.17 (d, *J* = 8.0 Hz, 2H), 6.39 (s, ^1^H), 2.36 ppm (s, 3H); ^13^C{^1^H} NMR (100 MHz, CDCl_3_): *δ* = 140.3, 137.1, 129.1, 128.6, 73.0, 54.9, 21.2 ppm.

**1-(bromoethynyl)-3-methylbenzene (2b)**: The crude product was purified by silica gel column chromatography with hexanes as the eluent to give a yellow oil (55.6 mg, 95%). ^1^H NMR (400 MHz, CDCl_3_): *δ* = 7.28–7.22 (m, 2H), 7.19 (t, *J* = 7.6 Hz, ^1^H), 7.14 (d, *J* = 7.6 Hz, ^1^H), 2.31 ppm (s, 3H); ^13^C{^1^H} NMR (100 MHz, CDCl_3_): *δ* = 138.1, 132.6, 129.6, 129.1, 128.2, 122.5, 80.2, 49.2, 21.2 ppm.

**(*E/Z*)-1-(1,2-dibromovinyl)-3-methylbenzene (3b/4b)**: The crude product was purified by silica gel column chromatography with hexanes as the eluent to give **3b** as a yellow oil (48 mg, 58%) and **4b** as yellow oil (2.5 mg, 3%), respectively. **3b**: ^1^H NMR (400 MHz, CDCl_3_): *δ* = 7.32–7.29 (m, 2H), 7.26 (d, *J* = 8.0 Hz, ^1^H), 7.17 (d, *J* = 7.2 Hz, ^1^H), 6.78 (s, ^1^H), 2.38 ppm (s, 3H); ^13^C{^1^H} NMR (100 MHz, CDCl_3_): *δ* = 138.1, 137.0, 130.2, 129.6, 128.2, 126.2, 121.6, 102.8, 77.3, 77.0, 76.7, 21.4 ppm **4b**: ^1^H NMR (400 MHz, CDCl_3_): *δ* = 7.31–7.28 (m, 2H), 7.22 (t, *J* = 8.4 Hz, ^1^H), 7.16 (d, *J* = 7.2 Hz, ^1^H), 7.03 (s, ^1^H), 2.37 ppm (s, 3H); ^13^C{^1^H} NMR (100 MHz, CDCl_3_): *δ* = 138.5, 138.4, 131.3, 130.2, 128.5, 128.4, 124.9, 108.5, 21.3 ppm.

**(*Z*)-1-(1,2-dibromovinyl)-3-methylbenzene (5b)**: The crude product was purified by silica gel column chromatography with 50:1 hexanes/EtOAc as the eluent to give a yellow oil (64 mg, 73%). ^1^H NMR (400 MHz, CDCl_3_): *δ* = 7.87–7.86 (m, 2H), 7.44 (d, *J* = 7.6 Hz, ^1^H), 7.39 (t, *J* = 8.0 Hz, ^1^H), 6.73 ppm (s, ^1^H); ^13^C{^1^H} NMR (100 MHz, CDCl_3_): *δ* = 186.2, 139.0, 135.3, 130.9, 130.1, 128.8, 126.8, 39.9, 21.4 ppm.

**1-methyl-3-(1,1,2,2-tetrabromoethyl)benzene (6b)**: The crude product was purified by silica gel column chromatography with 100:1 hexanes/EtOAc as the eluent to give a white solid (83.7 mg, 64%). ^1^H NMR (400 MHz, CDCl_3_): *δ* = 7.66–7.64 (m, 2H), 7.26 (t, *J* = 7.6 Hz, ^1^H), 7.17 (d, *J* = 7.6 Hz, ^1^H), 6.41 (s, ^1^H), 2.40 ppm (s, 3H); ^13^C{^1^H} NMR (100 MHz, CDCl_3_): *δ* = 139.8, 138.2, 130.8, 129.2, 128.3, 125.7, 73.0, 54.7, 21.6 ppm.

**(bromoethynyl)benzene (2c)**: The crude product was purified by silica gel column chromatography with hexanes as the eluent to give a brown oil (45.6 mg, 84%). ^1^H NMR (400 MHz, CDCl_3_): *δ* = 7.44 (d, *J* = 8.0 Hz, 2H), 7.34–7.27 ppm (m, 3H); ^13^C{^1^H} NMR (100 MHz, CDCl_3_): *δ* = 132.0, 128.7, 128.4, 122.7, 80.1, 49.7 ppm.

**(*E/Z*)-(1,2-dibromovinyl)benzene (3c/4c)**: The crude product was purified by silica gel column chromatography with hexanes as the eluent to give **3c** as a brown oil (35.4 mg, 45%) and **4c** as brown oil (2.4 mg, 3%), respectively. **3c**: ^1^H NMR (400 MHz, CDCl_3_): *δ* = 7.50 (d, *J* = 8.0 Hz, 2H), 7.42–7.34 (m, 3H), 6.79 ppm (s, ^1^H); ^13^C{^1^H} NMR (100 MHz, CDCl_3_): *δ* = 136.0, 128.4, 128.1, 127.2, 120.3, 102.0 ppm **4c**: ^1^H NMR (400 MHz, CDCl_3_): *δ* = 7.52–7.49 (m, 2H), 7.38–7.32 (m, 3H), 7.06 ppm (s, ^1^H); ^13^C{^1^H} NMR (100 MHz, CDCl_3_): *δ* = 137.5, 130.1, 128.4, 127.6, 126.7, 107.8 ppm.

**2,2-dibromo-1-phenylethanone (5c)**: The crude product was purified by silica gel column chromatography with 50:1 hexanes/EtOAc as the eluent to give a brown oil (60 mg, 72%). ^1^H NMR (400 MHz, CDCl_3_): *δ* = 8.08 (d, *J* = 8.4 Hz, 2H), 7.63 (t, *J* = 7.2 Hz, ^1^H), 7.51 (t, *J* = 8.0 Hz, 2H), 6.72 ppm (s, ^1^H). ^13^C{^1^H} NMR (100 MHz, CDCl_3_): *δ* = 184.9, 133.4, 129.9, 128.7, 127.9, 38.7 ppm.

**(1,1,2,2-tetrabromoethyl)benzene (6c)**: The crude product was purified by silica gel column chromatography with 100:1 hexanes/EtOAc as the eluent to give a brown oil (81 mg, 64%). ^1^H NMR (500 MHz, CDCl_3_): *δ* = 7.89–7.87 (m, 2H), 7.40–7.38 (m, 3H), 6.41 ppm (s, ^1^H); ^13^C{^1^H} NMR (125 MHz, CDCl_3_): *δ* = 139.8, 130.0, 128.7, 128.4, 72.7, 54.7 ppm.

**1-(bromoethynyl)-4-methoxybenzene (2d)**: The crude product was purified by silica gel column chromatography with 100:1 hexanes/EtOAc as the eluent to give a brown solid (43.7 mg, 69%). ^1^H NMR (400 MHz, CDCl_3_): *δ* = 7.37 (d, *J* = 8.4 Hz, ^1^H), 6.82 (d, *J* = 8.5 Hz, ^1^H), 3.79 ppm (s, 2H); ^13^C{^1^H} NMR (100 MHz, CDCl_3_): *δ* = 159.9), 133.5, 114.8, 114.0, 80.0, 55.3, 47.8 ppm.

**(*E/Z*)-1-(1,2-dibromovinyl)-4-methoxybenzene (3d/4d)**: The crude product was purified by silica gel column chromatography with 100:1 hexanes/EtOAc as the eluent to give **3d** as a brown solid (71.8 mg, 82%) and **4d** as brown solid (2.6 mg, 3%), respectively. **3d**: ^1^H NMR (400 MHz, CDCl_3_): *δ* = 7.48 (d, *J* = 8.8 Hz, 2H), 6.89 (d, *J* = 8.8 Hz, 2H), 6.73 (s, ^1^H), 3.82 ppm (s, 3H); ^13^C{^1^H} NMR (100 MHz, CDCl_3_): *δ* = 160.2, 130.8, 129.2, 121.5, 113.6, 102.0, 55.4 ppm **4d**: ^1^H NMR (400 MHz, CDCl_3_): δ = 7.44 (d, *J* = 8.8 Hz, 2H), 6.94 (s, ^1^H), 6.86 (d, *J* = 8.8 Hz, 2H), 3.82 ppm (s, 3H); ^13^C{^1^H} NMR (100 MHz, CDCl_3_): δ = 159.5, 130.1, 129.9, 128.1, 112.9, 106.0, 54.4 ppm.

**2,2-dibromo-1-(4-methoxyphenyl)ethanone (5d)**: The crude product was purified by silica gel column chromatography with 30:1 hexanes/EtOAc as the eluent to give a light brown solid (80.4 mg, 87%). ^1^H NMR (500 MHz, CDCl_3_): *δ* = 8.08 (d, *J* = 9.0 Hz, 2H), 6.97 (d, *J* = 8.0 Hz, 2H), 6.68 (s, ^1^H), 3.90 ppm (s, 3H);^13^C{^1^H} NMR (125 MHz, CDCl_3_): *δ* = 184.7, 164.6, 132.3, 123.4, 114.2, 55.7, 39.9 ppm.

**methyl 4-(bromoethynyl)benzoate (2e)**: The crude product was purified by silica gel column chromatography with 100:1 hexanes/EtOAc as the eluent to give a light yellow solid (63.1 mg, 88%). ^1^H NMR (400 MHz, CDCl_3_): *δ* = 7.98 (d, *J* = 8.4 Hz, 2H), 7.50 (d, *J* = 8.4 Hz, 2H), 3.92 ppm (s, 3H); ^13^C{^1^H} NMR (100 MHz, CDCl_3_): *δ* = 165.4, 130.9, 129.0, 128.5, 126.3, 78.4, 52.3, 51.3 ppm.

**(*E*/*Z*)-methyl 4-(1,2-dibromovinyl)benzoate (3e/4e)**: The crude product was purified by silica gel column chromatography with 100:1 hexanes/EtOAc as the eluent to give **3e** as a brownish yellow solid (53.8 mg, 56%) and **4e** as brownish yellow solid (mg, 8%), respectively. **3e**: mp:73–75°C; ^1^H NMR (400 MHz, CDCl_3_): *δ* = 8.06 (d, *J* = 8.4 Hz, 2H), 7.58 (d, *J* = 8.4 Hz, 2H), 6.87 (s, ^1^H), 3.93 ppm (s, 3H); ^13^C{^1^H} NMR (100 MHz, CDCl_3_): *δ* = 165.3, 140.3, 129.8, 128.5, 128.2, 119.0, 103.4, 51.3 ppm; IR (KBr): 3,078, 3,069, 2,958, 2,924, 2,853, 1724, 1,603, 1,564, 1,438, 1,430, 1,312, 1,286, 1,182, 1,286, 1,183, 1,110, 1,019, 964, 883, 853, 822, 797, 780, 769, 717, 698, 633, 564 cm^−1^.**4e**: mp:77–79°C; ^1^H NMR (400 MHz, CDCl_3_): *δ* = 8.01 (d, *J* = 8.8 Hz, 2H), 7.57 (d, *J* = 8.8 Hz, 2H), 7.19 (s, ^1^H), 3.92 ppm (s, 3H); ^13^C{^1^H} NMR (100 MHz, CDCl_3_): *δ* = 165.2, 141.3, 129.8, 129.0, 128.8, 126.6, 109.9, 51.3 ppm; IR (KBr): 3,063, 3,039, 3,002, 2,953, 2,853, 1941, 1725, 1,606, 1,584, 1,500, 1,430, 1,407, 1,318, 1,278, 1,218, 1,186, 1,110, 1,020, 970, 895, 862, 801, 769, 745, 697, 652, 542 cm^−1^.

**methyl 4-(2,2-dibromoacetyl)benzoate (5e)**: The crude product was purified by silica gel column chromatography with 30:1 hexanes/EtOAc as the eluent to give a brown solid (9.1 mg, 9%). Mp: 79–80°C; ^1^H NMR (400 MHz, CDCl_3_): *δ* = 8.16 (s, 4H), 6.66 (s, ^1^H), 3.97 ppm (s, 3H); ^13^C{^1^H} NMR (100 MHz, CDCl_3_): *δ* = 184.5, 164.8, 134.0, 133.2, 129.0, 128.7, 51.6, 38.3 ppm; IR (KBr): 2,952, 2,925, 2,854, 1721, 1,690, 1,604, 1,571, 1,528, 1,504, 1,436, 1,406, 1,277, 1,190, 1,108, 1,018, 987, 966, 869, 793, 733, 715, 684, 655, 559 cm^−1^.

**methyl 4-(1,1,2,2-tetrabromoethyl)benzoate (6e)**: The crude product was purified by silica gel column chromatography with 50:1 hexanes/EtOAc as the eluent to give a brownish yellow solid (21.6 mg, 15%). Mp: 66–68°C; ^1^H NMR (400 MHz, CDCl_3_): *δ* = 8.05 (d, *J* = 8.8 Hz, 2H), 7.96 (d, *J* = 8.8 Hz, 2H), 6.41 (s, ^1^H), 3.94 (s, 3H) ppm; ^13^C{^1^H} NMR (100 MHz, CDCl_3_): *δ* = 164.9, 143.1, 130.4, 128.5, 127.8, 70.1, 52.7, 51.4 ppm; IR (KBr): 2,994, 2,955, 2,924, 2,853, 1716, 1,608, 1,432, 1,397, 1,313, 1,289, 1,261, 1,184, 1,137, 1,111, 1,018, 960, 863, 840, 766, 736, 707, 691, 671, 629, 607, 570 cm^−1^.

**1-(bromoethynyl)-4-fluorobenzene (2f)**: The crude product was purified by silica gel column chromatography with hexanes as the eluent to give a yellow oil (58.5 mg, 98%). ^1^H NMR (400 MHz, CDCl_3_): *δ* = 7.44–7.41 (m, 2H), 7.02–6.98 (m, 2H) ppm; ^13^C{^1^H} NMR (100 MHz, CDCl_3_): *δ* = 162.7(d, *J* = 248.6 Hz), 133.9 (d, *J* = 8.4 Hz), 118.8 (d, *J* = 3.6 Hz), 115.78 (d, *J* = 22.1 Hz), 79.0, 49.5 ppm.

**(*E/Z*)-1-(1,2-dibromovinyl)-4-fluorobenzene (3f/4f)**: The crude product was purified by silica gel column chromatography with hexanes as the eluent to give **3f** as a yellow oil (46.2 mg, 55%) and **4f** as a yellow oil (46.2 mg, 55%) and **4f** as yellow oil (5 mg, 6%), respectively. **3f**: ^1^H NMR (400 MHz, CDCl_3_): *δ* = 7.52–7.49 (m, 2H), 7.10–7.05 (m, 2H), 6.80 ppm (s, ^1^H); ^13^C{^1^H} NMR (100 MHz, CDCl_3_): *δ* = 162.9 (d, *J* = 248.9 Hz), 133.1 (d, *J* = 3.5 Hz), 131.3 (d, *J* = 8.5 Hz), 120.3, 115.5 (d, *J* = 21.9 Hz), 103.4 ppm **4f**: ^1^H NMR (400 MHz, CDCl_3_): *δ* = 7.51–7.47 (m, 2H), 7.07–7.02 (m, 2H), 7.01 ppm (s, ^1^H); ^13^C{^1^H} NMR (100 MHz, CDCl_3_): *δ* = 163.2 (d, *J* = 248.8 Hz), 134.7 (d, *J* = 3.3 Hz), 129.9, 129.6 (d, *J* = 8.4 Hz), 115.6 (d, *J* = 21.8 Hz), 108.7 ppm.

**2,2-dibromo-1-(4-fluorophenyl)ethanone (5f)**: The crude product was purified by silica gel column chromatography with 50:1 hexanes/EtOAc as the eluent to give a yellow oil (69.2 mg, 78%). ^1^H NMR (400 MHz, CDCl_3_): *δ* = 8.17–8.14 (m, 2H), 7.21–7.16 (m, 2H), 6.63 ppm (s, ^1^H); ^13^C{^1^H} NMR (100 MHz, CDCl_3_): *δ* = 184.6, 166.4 (d, *J* = 256.4 Hz), 132.7(d, *J* = 9.6 Hz), 127.1 (d, *J* = 3.1 Hz), 116.3 (d, *J* = 22 Hz), 39.4 ppm.

**1-fluoro-4-(1,1,2,2-tetrabromoethyl)benzene (6f)**: The crude product was purified by silica gel column chromatography with 100:1 hexanes/EtOAc as the eluent to give a yellow oil (69.9 mg, 53%). ^1^H NMR (400 MHz, CDCl_3_): *δ* = 7.91–7.88 (m, 2H), 7.09–7.05 (m, 2H), 6.35 ppm (s, ^1^H); ^13^C{^1^H} NMR (100 MHz, CDCl_3_): *δ* = 163.1 (d, *J* = 250.6 Hz), 135.6 (d, *J* = 3.1 Hz), 131.0 (d, *J* = 8.4 Hz), 115.1 (d, *J* = 21.9 Hz), 71.3, 54.6 ppm.

**1-(bromoethynyl)-4-chlorobenzene (2g)**: The crude product was purified by silica gel column chromatography with hexanes as the eluent to give a white solid (64 mg, 99%). ^1^H NMR (400 MHz, CDCl_3_): *δ* = 7.37 (d, *J* = 8.8 Hz, 2H), 7.28 ppm (d, *J* = 8.4 Hz, 2H); ^13^C{^1^H} NMR (100 MHz, CDCl_3_): *δ* = 134.8, 133.2, 128.7, 121.2, 79.0, 51.0 ppm.

**(*E/Z*)-1-chloro-4-(1,2-dibromovinyl)benzene (3g/4g)**: The crude product was purified by silica gel column chromatography with hexanes as the eluent to give **3g** as a white solid (39.1 mg, 44%) and **4g** as white solid (3.6 mg, 4%), respectively. **3g**: ^1^H NMR (400 MHz, CDCl_3_): *δ* = 7.45 (d, *J* = 8.4 Hz, 2H), 7.36 (d, *J* = 8.8 Hz, 2H), 6.81 ppm (s, ^1^H); ^13^C NMR (100 MHz, CDCl_3_): *δ* = 135.5, 135.4, 130.6, 128.6, 120.1, 103.8 ppm **4g**: ^1^H NMR (400 MHz, CDCl_3_): *δ* = 7.44 (d, *J* = 8.4 Hz, 2H), 7.33 (d, *J* = 8.8 Hz, 2H), 7.06 ppm (s, ^1^H); ^13^C{^1^H} NMR (100 MHz, CDCl_3_): *δ* = 136.9, 135.5, 129.9, 128.9,128.8, 109.5 ppm.

**2,2-dibromo-1-(4-chlorophenyl)ethanone (5g)**: The crude product was purified by silica gel column chromatography with 50:1 hexanes/EtOAc as the eluent to give a white solid (64.7 mg, 69%). ^1^H NMR (400 MHz, CDCl_3_): *δ* = 8.05 (d, *J* = 8.8 Hz, 2H), 7.48 (d, *J* = 8.8 Hz, 2H), 6.62 ppm (s, ^1^H); ^13^C{^1^H} NMR (100 MHz, CDCl_3_): *δ* = 184.9, 141.1, 131.2, 129.3, 129.1, 39.3 ppm.

**1-chloro-4-(1,1,2,2-tetrabromoethyl)benzene (6g)**: The crude product was purified by silica gel column chromatography with 100:1 hexanes/EtOAc as the eluent to give a yellow oil (42.4 mg, 31%). ^1^H NMR (400 MHz, CDCl_3_): *δ* = 7.83 (d, *J* = 8.8 Hz, 2H), 7.36 (d, *J* = 8.8 Hz, 2H), 6.35 ppm (s, ^1^H); ^13^C{^1^H} NMR (100 MHz, CDCl_3_): *δ* = 138.3, 136.2, 130.2, 128.4, 71.1, 54.2 ppm.

**bromo-4-(bromoethynyl)benzene (2h)**: The crude product was purified by silica gel column chromatography with hexanes as the eluent to give a white solid (60.8 mg, 78%). ^1^H NMR (400 MHz, CDCl_3_): *δ* = 7.45 (d, *J* = 8.8 Hz, 2H), 7.30 ppm (d, *J* = 8.4 Hz, 2H); ^13^C{^1^H} NMR (100 MHz, CDCl_3_): *δ* = 133.4, 131.6, 123.0, 121.7, 79.1, 51.2 ppm.

**(*E/Z*)-1-bromo-4-(1,2-dibromovinyl)benzene (3h/4h)**: The crude product was purified by silica gel column chromatography with hexanes as the eluent to give **3h** as a light yellow solid (42.9 mg, 42%) and **4h** as light yellow solid (4.1 mg, 4%), respectively. 3 h: ^1^H NMR (400 MHz, CDCl_3_): *δ* = 7.53 (d, *J* = 8.8 Hz, 2H), 7.38 (d, *J* = 8.4 Hz, 2H), 6.82 ppm (s, ^1^H); ^13^C{^1^H} NMR (100 MHz, CDCl_3_): *δ* = 135.9, 131.6, 130.8, 123.7, 120.1, 103.8 ppm **4h**: ^1^H NMR (400 MHz, CDCl_3_): *δ* = 7.49 (d, *J* = 8.8 Hz, 2H), 7.37 (d, *J* = 8.4 Hz, 2H), 7.07 (s, ^1^H); ^13^C{^1^H} NMR (100 MHz, CDCl_3_): *δ* = 137.4, 131.8, 129.9, 129.2, 123.7, 109.5 ppm.

**2,2-dibromo-1-(4-bromophenyl)ethanone (5h)**: The crude product was purified by silica gel column chromatography with 50:1 hexanes/EtOAc as the eluent to give a light yellow solid (57.8 mg, 54%). ^1^H NMR (400 MHz, CDCl_3_): *δ* = 7.97 (d, *J* = 8.8 Hz, 2H), 7.66 (d, *J* = 8.8 Hz, 2H), 6.59 ppm (s, ^1^H); ^13^C{^1^H} NMR (100 MHz, CDCl_3_): *δ* = 185.1, 132.3, 131.2, 129.9, 129.5 39.2 ppm.

**1-bromo-4-(1,1,2,2-tetrabromoethyl)benzene (6h)**: The crude product was purified by silica gel column chromatography with 100:1 hexanes/EtOAc as the eluent to give a yellow oil (33 mg, 22%). ^1^H NMR (400 MHz, CDCl_3_): *δ* = 7.76 (d, *J* = 8.8 Hz, 2H), 7.51 (d, *J* = 9.2 Hz, 2H), 6.35 ppm (s, ^1^H); ^13^C{^1^H} NMR (100 MHz, CDCl_3_): *δ* = 138.8, 131.4, 130.4, 124.5, 71.2, 54.0 ppm.

**1-(bromoethynyl)-4-(trifluoromethyl)benzene (2i)**: The crude product was purified by silica gel column chromatography with hexanes as the eluent to give a brown solid (74 mg, 99%). ^1^H NMR (400 MHz, CDCl_3_): *δ* = 7.59–7.53 ppm (m, 4H); ^13^C{^1^H} NMR (100 MHz, CDCl_3_): *δ* = 137.5, 132.3, 130.5 (q, *J* = 32.5 Hz), 125.3 (q, *J* = 32.5 Hz), 125.2 (q, *J* = 270.6 Hz), 78.8, 53.0 ppm.

**(*E/Z*)-1-(1,2-dibromovinyl)-4-(trifluoromethyl)benzene (3i/4i)**: The crude product was purified by silica gel column chromatography with hexanes as the eluent to give **3i** as a brown solid (53.5 mg, 54%) and **4i** as brown solid (10.9 mg, 11%), respectively. **3i**: ^1^H NMR (400 MHz, CDCl_3_): *δ* = 7.67–7.61 (m, 4H), 6.89 (s, ^1^H); ^13^C{^1^H} NMR (100 MHz, CDCl_3_): *δ* = 140.6, 131 (q, *J* = 32.7 Hz), 129.6, 126.4(q, *J* = 270.6 Hz), 125.4 (q, *J* = 3.7 Hz), 119.4, 104.7.**4i**: ^1^H NMR (400 MHz, CDCl_3_): *δ* = 7.66 (s, 4H), 7.17 ppm (s, ^1^H); ^13^C{^1^H} NMR (100 MHz, CDCl_3_): *δ* = 141.7, 131.3 (q, *J* = 32.6 Hz), 129.5, 126.4 (q, *J* = 270.6 Hz), 125.6 (q, *J* = 3.8 Hz), 119.7, 111.1 ppm.

**2,2-dibromo-1-(4-(trifluoromethyl)phenyl)ethanone (5i)**: The crude product was purified by silica gel column chromatography with 50:1 hexanes/EtOAc as the eluent to give a brownish yellow solid (9.3 mg, 9%). ^1^H NMR (400 MHz, CDCl_3_): *δ* = 8.23 (d, *J* = 8.4 Hz, 2H), 7.78 (d, *J* = 8.4 Hz, 2H), 6.62 ppm (s, ^1^H); ^13^C{^1^H} NMR (100 MHz, CDCl_3_): *δ* = 185.0, 135.5 (q, *J* = 32.8 Hz), 133.7, 130.2, 125.9 (q, *J* = 3.7 Hz), 126.0 (q, *J* = 270 Hz), 39.0 ppm.

**1-(1,1,2,2-tetrabromoethyl)-4-(trifluoromethyl)benzene (6i)**: The crude product was purified by silica gel column chromatography with 100:1 hexanes/EtOAc as the eluent to give **6i** as a yellow solid (20.6 mg, 14%). Mp: 63–65°C; ^1^H NMR (400 MHz, CDCl_3_): *δ* = 8.03 (d, *J* = 8.4 Hz, 2H), 7.65 (d, *J* = 8.4 Hz, 2H), 6.40 ppm (s, ^1^H); ^13^C{^1^H} NMR (100 MHz, CDCl_3_): *δ* = 143.3, 131.9 (q, *J* = 32.8 Hz), 129.3, 125.3 (q, *J* = 3.7 Hz), 123.5 (q, *J* = 270.8 Hz), 70.4, 53.6 ppm. IR (KBr): 2,987, 2,920, 2,830, 1,617, 1,408, 1,328, 1,157, 1,136, 1,070, 1,015, 842, 760, 726, 701, 643, 621 cm^−1^.

**1-(bromoethynyl)-4-nitrobenzene (2j)**: The crude product was purified by silica gel column chromatography with 50:1 hexanes/EtOAc as the eluent to give a brown solid (57.6 mg, 85%). ^1^H NMR (400 MHz, CDCl_3_): *δ* = 8.19 (d, *J* = 8.8 Hz, 2H), 7.59 ppm (d, *J* = 8.8 Hz, 2H); ^13^C{^1^H} NMR (100 MHz, CDCl_3_): *δ* = 146.4, 131.8, 128.5, 122.6, 77.4, 55.3 ppm.

**(*E/Z*)-1-(1,2-dibromovinyl)-4-nitrobenzene (3j/4j)**: The crude product was purified by silica gel column chromatography with 50:1 hexanes/EtOAc as the eluent to give **3j** as a brown solid (35.9 mg, 39%) and **4j** as brown solid (9.2 mg, 10%), respectively. **3j**: ^1^H NMR (400 MHz, CDCl_3_): *δ* = 8.26 (d, *J* = 8.8 Hz, 2H), 7.69 (d, *J* = 8.8 Hz, 2H), 6.95 ppm (s, ^1^H); ^13^C{^1^H} NMR (100 MHz, CDCl_3_): *δ* = 146.9, 142.3, 129.3, 122.6, 117.4, 104.8 ppm **4j**: ^1^H NMR (400 MHz, CDCl_3_): *δ* = 8.22 (d, *J* = 8.8 Hz, 2H), 7.69 (d, *J* = 9.2 Hz, 2H), 7.29 ppm (s, ^1^H); ^13^C{^1^H} NMR (100 MHz, CDCl_3_): *δ* = 148.1, 144.1, 128.7, 128.5, 123.9, 112.8 ppm.

**2,2-dibromo-1-(4-nitrophenyl)ethanone (5j)**: The crude product was purified by silica gel column chromatography with 20:1 hexanes/EtOAc as the eluent to give **5j** as a brown oil (2 mg, 2%). ^1^H NMR (400 MHz, CDCl_3_): *δ* = 8.25 (d, *J* = 8.8 Hz, 2H), 7.75 (d, *J* = 8.8 Hz, 2H), 6.67 ppm (s, ^1^H); ^13^C{^1^H} NMR (100 MHz, CDCl_3_): *δ* = 201.7, 145.4, 128.3, 126.7, 123.0, 53.6 ppm.

**1-nitro-4-(1,1,2,2-tetrabromoethyl)benzene (6j)**: The crude product was purified by silica gel column chromatography with 30:1 hexanes/EtOAc as the eluent to give **6j** as a brown solid (25.2 mg, 18%). Mp: 77–79°C; ^1^H NMR (400 MHz, CDCl_3_): *δ* = 8.25 (d, *J* = 9.2 Hz, 2H), 8.10 (d, *J* = 8.8 Hz, 2H), 6.40 ppm (s, ^1^H); ^13^C{^1^H} NMR (100 MHz, CDCl_3_): *δ* = 147.3, 129.1, 123.0, 122.3, 82.7, 52.0 ppm; IR (KBr): 3,103, 3,002, 2,961, 2,924, 2,850, 1,600, 1,513, 1,485, 1,400, 1,346, 1,318, 1,261, 1,166, 1,136, 1,110, 1,014, 867, 845, 815, 765, 752, 705, 676, 608, 570 cm^−1^.

## Data Availability

The original contributions presented in the study are included in the article/Supplementary Material, further inquiries can be directed to the corresponding authors.
