# Supplementary material for: Hypervalent Iodine-Mediated Chemoselective Bromination of Terminal Alkynes
Source: Front Chem. 2022 Apr 7;10:879789. doi: 10.3389/fchem.2022.879789 (PMC9021491; doi:10.3389/fchem.2022.879789)
Supplement: Supplementary file 1 [file DataSheet1.pdf]

Supplementary Material

Hypervalent Iodine Mediated Chemoselective Bromination of Terminal Alkynes

Youzhi Li, [1] Xuemei Chen,[1] Daya Huang, [1] Zhenming Xie,*[1] Yan Liu*[1,2]

[1] School of Chemical Engineering and Light Industry, Guangdong University of Technology, Guangzhou, 510006, China.
[2] Guangdong Provincial Key Laboratory of Plant Resources Biorefinery, Guangdong University of Technology, Guangzhou, 510006, China

Table of contents

1.	General information	S2
2.	General procedures for the bromination of alkynes	S2-S3
3.	Spectral data for selected products	S3-S13
4.	NMR spectra of products	S14-S63

1. General information.

^{1}H NMR and ^{13}C{1H} NMR spectra were recorded on a Bruker AVANCE III 400 MHz or 500 MHz spectrometer (400 MHz or 500 MHz for ^{1}H NMR, 100 MHz or 125 MHz for ^{13}C NMR). Tetramethylsilane (TMS) was used as an internal standard (0 ppm) for the ^{1}H NMR spectra, and $CDCl_3$ was used as the internal standard (77.0 ppm) for the ^{13}C{1H} NMR spectra. Reactions were monitored by thin-layer chromatography (TLC). Reaction products were purified by column chromatography on silica gel (300-400 mesh). Chemical reagents were purchased from common commercial suppliers and used as received.

2. General Procedures for the Bromination of Alkynes.

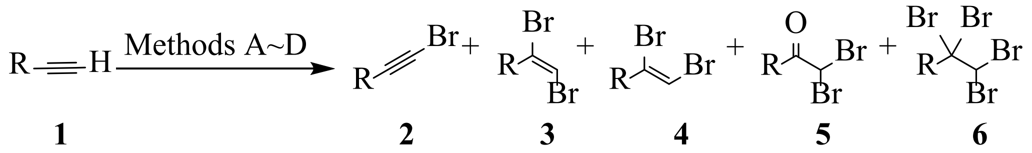

Method A. PIDA (193.2 mg, 0.6 mmol) was added in portions over a period of 15 min to a mixture of the alkyne (0.3 mmol) and TBAB (133.0 mg, 0.36 mmol) in CH_3CN (3 mL), and the reaction mixture was stirred at room temperature for 1~3h. The reaction progress was monitored by TLC. Upon completion, the reaction mixture was quenched with saturated aqueous $Na_2S_2O_3$, washed with brine, extracted with ethyl acetate, and dried over anhydrous Na_2SO_4. After filtration, the solvent was removed under reduced pressure to afford the crude product, which was purified by column chromatography using hexane or hexane/ethyl acetate and analyzed by ^{1}H and ^{13}C NMR spectroscopy.

Method B. PIDA (96.6 mg, 0.3 mmol) was added in portions over a period of 15 min to a mixture of the alkyne (0.3 mmol) and NaBr (123.5 mg, 1.2 mmol) in CH_3CN (3 mL), and the reaction mixture was stirred at room temperature for 1~5 h. The reaction progress was monitored by TLC. Upon completion, the reaction mixture was quenched with saturated aqueous $Na_2S_2O_3$, washed with brine, extracted with ethyl acetate, and dried over anhydrous Na_2SO_4. After filtration, the solvent was removed under reduced pressure to afford the crude product, which was purified by column chromatography using hexane or hexane/ethyl acetate and analyzed by ^{1}H and ^{13}C NMR spectroscopy.

Method C. PIDA (289.8 mg, 0.9 mmol) was added in portions over a period of 15 min to a mixture of the alkyne (0.3 mmol) and NaBr (92.6 mg, 0.9 mmol) in CH_3CN (1 mL) and H_2O (3 mL), and the reaction mixture was stirred at room temperature for 10 h. The reaction progress was monitored by TLC. Upon completion, the reaction mixture was quenched with saturated aqueous $Na_2S_2O_3$, washed with brine, extracted with ethyl acetate, and dried over anhydrous Na_2SO_4. After filtration, the solvent was removed under reduced pressure to afford the crude product, which was purified by column chromatography using hexane or hexane/ethyl acetate and analyzed by ^{1}H and ^{13}C NMR spectroscopy.

Method D. PIDA (386.4 mg, 1.2 mmol) was added in portions over a period of 15 min to a mixture of the alkyne (0.3 mmol) and NaBr (185.2.0 mg, 1.8 mmol) in CH_3CN (3 mL). After stirring at room temperature for 3 h, NaBr (92.6 mg, 0.9 mmol) were added. More PIDA (96.6 mg, 0.3 mmol) was added in portions over a period of 15 min, and the reaction mixture was stirred at room temperature for 3 h. The reaction progress was monitored by TLC. Upon completion, the reaction mixture was quenched with saturated aqueous $Na_2S_2O_3$, washed with brine, extracted with ethyl acetate, and dried over anhydrous Na_2SO_4. After filtration, the solvent was removed under reduced pressure to afford the crude product, which was purified by column chromatography using hexane or hexane/ethyl acetate and analyzed by ^{1}H and ^{13}C NMR spectroscopy.

3. Spectral data for selected products

1-(bromoethynyl)-4-methylbenzene (2a):

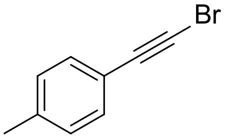

(*E*)-1-(1,2-dibromovinyl)-4-methylbenzene (3a):

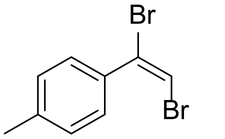

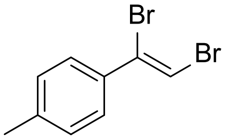

2,2-dibromo-1-(p-tolyl)ethanone (5a):

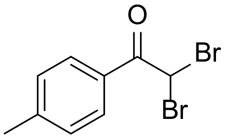

1-methyl-4-(1,1,2,2-tetrabromoethyl)benzene (6a):

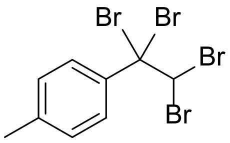

6a: The crude product was purified by silica gel column chromatography with 100:1 hexanes/EtOAc as the eluent to give a yellow oil (121.6 mg, 93%). ^{1}H NMR (400 MHz, $CDCl_3$): δ = 7.74 (d, J = 8.0 Hz, 2H), 7.17

(d, J = 8.0 Hz, 2H), 6.39 (s, 1H), 2.36 ppm (s, 3H); $^{13}C\{^1H\}$ NMR (100 MHz, $CDCl_3$): δ =140.3, 137.1, 129.1, 128.6, 73.0, 54.9, 21.2 ppm.

1-(bromoethynyl)-3-methylbenzene (2b):

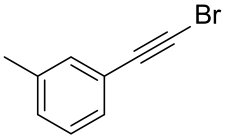

2b: The crude product was purified by silica gel column chromatography with hexanes as the eluent to give a yellow oil (55.6 mg, 95%). ^1H NMR (400 MHz, $CDCl_3$): δ = 7.28–7.22 (m, 2H), 7.19 (t, J = 7.6 Hz, 1H), 7.14 (d, J = 7.6 Hz, 1H), 2.31 ppm (s, 3H); $^{13}C\{^1H\}$ NMR (100 MHz, $CDCl_3$): δ =138.1, 132.6, 129.6, 129.1, 128.2, 122.5, 80.2, 49.2, 21.2 ppm.

(*E*)-1-(1,2-dibromovinyl)-3-methylbenzene (3b):

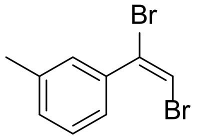

(*Z*)-1-(1,2-dibromovinyl)-3-methylbenzene (4b):

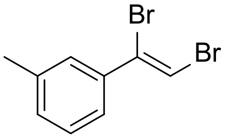

3b/4b: The crude product was purified by silica gel column chromatography with hexanes as the eluent to give **3b** as a yellow oil (48 mg, 58%) and **4b** as yellow oil (2.5 mg, 3%), respectively. **3b**: ^1H NMR (400 MHz, $CDCl_3$): δ = 7.32–7.29 (m, 2H), 7.26 (d, J = 8.0 Hz, 1H), 7.17 (d, J = 7.2 Hz, 1H), 6.78 (s, 1H), 2.38 ppm (s, 3H); $^{13}C\{^1H\}$ NMR (100 MHz, $CDCl_3$): δ = 138.1, 137.0, 130.2, 129.6, 128.2, 126.2, 121.6, 102.8, 77.3, 77.0, 76.7, 21.4 ppm. **4b**: ^1H NMR (400 MHz, $CDCl_3$): δ = 7.31–7.28 (m, 2H), 7.22 (t, J = 8.4 Hz, 1H), 7.16 (d, J = 7.2 Hz, 1H), 7.03 (s, 1H), 2.37 ppm (s, 3H); $^{13}C\{^1H\}$ NMR (100 MHz, $CDCl_3$): δ = 138.5, 138.4, 131.3, 130.2, 128.5, 128.4, 124.9, 108.5, 21.3 ppm.

(*Z*)-1-(1,2-dibromovinyl)-3-methylbenzene (5b):

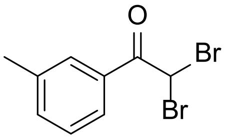

5b: The crude product was purified by silica gel column chromatography with 50:1 hexanes/EtOAc as the eluent to give a yellow oil (64 mg, 73%). ^1H NMR (400 MHz, $CDCl_3$): δ = 7.87–7.86 (m, 2H), 7.44 (d, J = 7.6 Hz, 1H), 7.39 (t, J = 8.0 Hz, 1H), 6.73 ppm (s, 1H); $^{13}C\{^1H\}$ NMR (100 MHz, $CDCl_3$): δ = 186.2, 139.0, 135.3, 130.9, 130.1, 128.8, 126.8, 39.9, 21.4 ppm.

1-methyl-3-(1,1,2,2-tetrabromoethyl)benzene (6b):

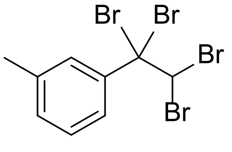

6b: The crude product was purified by silica gel column chromatography with 100:1 hexanes/EtOAc as the eluent to give a white solid (83.7 mg, 64%). ^{1}H NMR (400 MHz, $CDCl_3$): δ = 7.66–7.64 (m, 2H), 7.26 (t, J = 7.6 Hz, 1H), 7.17 (d, J = 7.6 Hz, 1H), 6.41 (s, 1H), 2.40 ppm (s, 3H); ^{13}C{^{1}H} NMR (100 MHz, $CDCl_3$): δ = 139.8, 138.2, 130.8, 129.2, 128.3, 125.7, 73.0, 54.7, 21.6 ppm.

(bromoethynyl)benzene (2c):

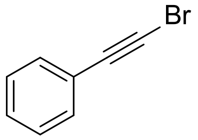

2c: The crude product was purified by silica gel column chromatography with hexanes as the eluent to give a brown oil (45.6 mg, 84%). ^{1}H NMR (400 MHz, $CDCl_3$): δ = 7.44 (d, J = 8.0 Hz, 2H), 7.34–7.27 ppm (m, 3H); ^{13}C{^{1}H} NMR (100 MHz, $CDCl_3$): δ = 132.0, 128.7, 128.4, 122.7, 80.1, 49.7 ppm.

(*E*)-(1,2-dibromovinyl)benzene (3c):

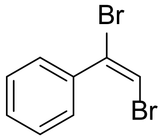

(*Z*)-(1,2-dibromovinyl)benzene (4c):

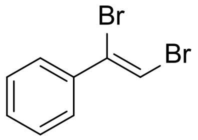

3c/4c: The crude product was purified by silica gel column chromatography with hexanes as the eluent to give **3c** as a brown oil (2.4 mg, 3%), respectively. **3c**: ^{1}H NMR (400 MHz, $CDCl_3$): δ = 7.50 (d, J = 8.0 Hz, 2H), 7.42–7.34 (m, 3H), 6.79 ppm (s, 1H); ^{13}C{^{1}H} NMR (100 MHz, $CDCl_3$): δ = 136.0, 128.4, 128.1, 127.2, 120.3, 102.0 ppm. **4c**: ^{1}H NMR (400 MHz, $CDCl_3$): δ = 7.52–7.49 (m, 2H), 7.38–7.32 (m, 3H), 7.06 ppm (s, 1H); ^{13}C{^{1}H} NMR (100 MHz, $CDCl_3$): δ = 137.5, 130.1, 128.4, 127.6, 126.7, 107.8 ppm.

2,2-dibromo-1-phenylethanone (5c):

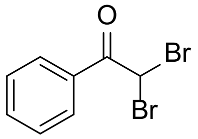

5c: The crude product was purified by silica gel column chromatography with 50:1 hexanes/EtOAc as the eluent to give a brown oil (60 mg, 72%). ^{1}H NMR (400 MHz, $CDCl_3$): δ = 8.08 (d, J = 8.4 Hz, 2H), 7.63 (t, J = 7.2 Hz, 1H), 7.51 (t, J = 8.0 Hz, 2H), 6.72 ppm (s, 1H). ^{13}C{^{1}H} NMR (100 MHz, $CDCl_3$): δ = 184.9, 133.4, 129.9, 128.7, 127.9, 38.7 ppm.

(1,1,2,2-tetrabromoethyl)benzene (6c):

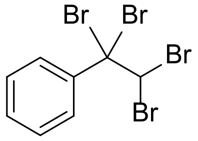

6c: The crude product was purified by silica gel column chromatography with 100:1 hexanes/EtOAc as the eluent to give a brown oil (81 mg, 64%). ^{1}H NMR (500 MHz, $CDCl_3$): δ = 7.89–7.87 (m, 2H), 7.40–7.38 (m, 3H), 6.41 ppm (s, 1H); $^{13}C\{^{1}H\}$ NMR (125 MHz, $CDCl_3$): δ = 139.8, 130.0, 128.7, 128.4, 72.7, 54.7 ppm.

1-(bromoethynyl)-4-methoxybenzene (2d):

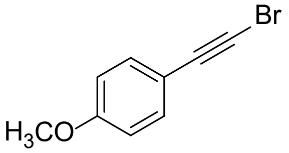

2d: The crude product was purified by silica gel column chromatography with 100:1 hexanes/EtOAc as the eluent to give a brown solid (43.7 mg, 69%). ^{1}H NMR (400 MHz, $CDCl_3$): δ = 7.37 (d, J = 8.4 Hz, 1H), 6.82 (d, J = 8.5 Hz, 1H), 3.79 ppm (s, 2H); $^{13}C\{^{1}H\}$ NMR (100 MHz, $CDCl_3$): δ = 159.9), 133.5, 114.8, 114.0, 80.0, 55.3, 47.8 ppm.

(*E*)-1-(1,2-dibromovinyl)-4-methoxybenzene (3d):

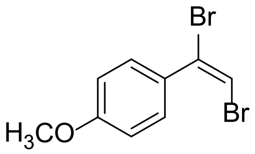

(*Z*)-1-(1,2-dibromovinyl)-4-methoxybenzene (4d):

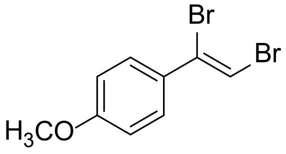

3d/4d: The crude product was purified by silica gel column chromatography with 100:1 hexanes/EtOAc as the eluent to give **3d** as a brown solid (71.8 mg, 82%) and **4d** as brown solid (2.6 mg, 3%), respectively. **3d**: ^{1}H NMR (400 MHz, $CDCl_3$): δ = 7.48 (d, J = 8.8 Hz, 2H), 6.89 (d, J = 8.8 Hz, 2H), 6.73 (s, 1H), 3.82 ppm (s, 3H); $^{13}C\{^{1}H\}$ NMR (100 MHz, $CDCl_3$): δ = 160.2, 130.8, 129.2, 121.5, 113.6, 102.0, 55.4 ppm. **4d**: ^{1}H NMR (400 MHz, $CDCl_3$): δ = 7.44 (d, J = 8.8 Hz, 2H), 6.94 (s, 1H), 6.86 (d, J = 8.8 Hz, 2H), 3.82 ppm (s, 3H); $^{13}C\{^{1}H\}$ NMR (100 MHz, $CDCl_3$): δ = 159.5, 130.1, 129.9, 128.1, 112.9, 106.0, 54.4 ppm.

2,2-dibromo-1-(4-methoxyphenyl)ethanone (5d):

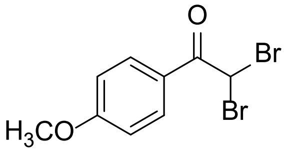

5d: The crude product was purified by silica gel column chromatography with 30:1 hexanes/EtOAc as the eluent to give a light brown solid (80.4 mg, 87%). ^{1}H NMR (500 MHz, $CDCl_3$): δ = 8.08 (d, J = 9.0 Hz, 2H), 6.97 (d, J = 8.0 Hz, 2H), 6.68 (s, 1H), 3.90 ppm (s, 3H);$^{13}C\{^{1}H\}$ NMR (125 MHz, $CDCl_3$): δ = 184.7, 164.6, 132.3, 123.4, 114.2, 55.7, 39.9 ppm.

methyl 4-(bromoethynyl)benzoate (2e):

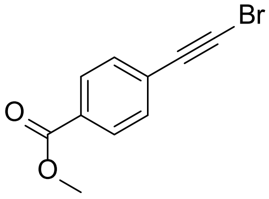

2e: The crude product was purified by silica gel column chromatography with 100:1 hexanes/EtOAc as the eluent to give a light yellow solid (63.1 mg, 88%). ^{1}H NMR (400 MHz, $CDCl_3$): δ = 7.98 (d, J = 8.4 Hz, 2H), 7.50 (d, J = 8.4 Hz, 2H), 3.92 ppm (s, 3H); $^{13}C\{^1H\}$ NMR (100 MHz, $CDCl_3$): δ = 165.4, 130.9, 129.0, 128.5, 126.3, 78.4, 52.3, 51.3 ppm.

(*E*)-methyl 4-(1,2-dibromovinyl)benzoate (3e):

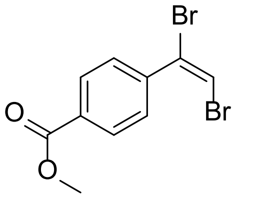

(*Z*)-methyl 4-(1,2-dibromovinyl)benzoate (4e):

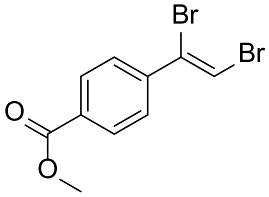

3e/4e: The crude product was purified by silica gel column chromatography with 100:1 hexanes/EtOAc as the eluent to give **3e** as a brownish yellow solid (53.8 mg, 56%) and **4e** as brownish yellow solid (mg, 8%), respectively. **3e**: mp: 73–75 °C; ^{1}H NMR (400 MHz, $CDCl_3$): δ = 8.06 (d, J = 8.4 Hz, 2H), 7.58 (d, J = 8.4 Hz, 2H), 6.87 (s, 1H), 3.93 ppm (s, 3H); $^{13}C\{^1H\}$ NMR (100 MHz, $CDCl_3$): δ = 165.3, 140.3, 129.8, 128.5, 128.2, 119.0, 103.4, 51.3 ppm; IR (KBr): 3078, 3069, 2958, 2924, 2853, 1724, 1603, 1564, 1438, 1430, 1312, 1286, 1182, 1286, 1183, 1110, 1019, 964, 883, 853, 822, 797, 780, 769, 717, 698, 633, 564 cm^{-1}. **4e**: mp: 77–79 °C; ^{1}H NMR (400 MHz, $CDCl_3$): δ = 8.01 (d, J = 8.8 Hz, 2H), 7.57 (d, J = 8.8 Hz, 2H), 7.19 (s, 1H), 3.92 ppm (s, 3H); $^{13}C\{^1H\}$ NMR (100 MHz, $CDCl_3$): δ = 165.2, 141.3, 129.8, 129.0, 128.8, 126.6, 109.9, 51.3 ppm; IR (KBr): 3063, 3039, 3002, 2953, 2853, 1941, 1725, 1606, 1584, 1500, 1430, 1407, 1318, 1278, 1218, 1186, 1110, 1020, 970, 895, 862, 801, 769, 745, 697, 652, 542 cm^{-1}.

methyl 4-(2,2-dibromoacetyl)benzoate (5e):

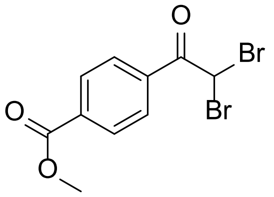

5e: The crude product was purified by silica gel column chromatography with 30:1 hexanes/EtOAc as the eluent to give a brown solid (9.1 mg, 9%). Mp: 79–80 °C; ^{1}H NMR (400 MHz, $CDCl_3$): δ = 8.16 (s, 4H), 6.66 (s, 1H), 3.97 ppm (s, 3H); $^{13}C\{^1H\}$ NMR (100 MHz, $CDCl_3$): δ = 184.5, 164.8, 134.0, 133.2, 129.0, 128.7, 51.6, 38.3 ppm; IR (KBr): 2952, 2925, 2854, 1721, 1690, 1604, 1571, 1528, 1504, 1436, 1406, 1277, 1190, 1108, 1018, 987, 966, 869, 793, 733, 715, 684, 655, 559 cm^{-1}.

methyl 4-(1,1,2,2-tetrabromoethyl)benzoate (6e):

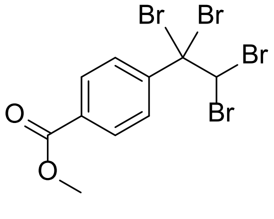

6e: The crude product was purified by silica gel column chromatography with 50:1 hexanes/EtOAc as the eluent to give a brownish yellow solid (21.6 mg, 15%). Mp: 66–68 °C; ^{1}H NMR (400 MHz, $CDCl_3$): δ = 8.05 (d, J = 8.8 Hz, 2H), 7.96 (d, J = 8.8 Hz, 2H), 6.41 (s, 1H), 3.94 (s, 3H) ppm; $^{13}C\{^1H\}$ NMR (100 MHz, $CDCl_3$): δ = 164.9, 143.1, 130.4, 128.5, 127.8, 70.1, 52.7, 51.4 ppm; IR (KBr): 2994, 2955, 2924, 2853, 1716, 1608, 1432, 1397, 1313, 1289, 1261, 1184, 1137, 1111, 1018, 960, 863, 840, 766, 736, 707, 691, 671, 629, 607, 570 cm^{-1}.

1-(bromoethynyl)-4-fluorobenzene (2f):

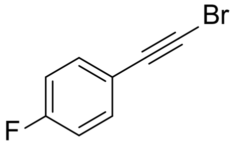

2f: The crude product was purified by silica gel column chromatography with hexanes as the eluent to give a yellow oil (58.5 mg, 98%). ^{1}H NMR (400 MHz, $CDCl_3$): δ = 7.44–7.41 (m, 2H), 7.02–6.98 (m, 2H) ppm; $^{13}C\{^1H\}$ NMR (100 MHz, $CDCl_3$): δ = 162.7(d, J = 248.6 Hz), 133.9 (d, J = 8.4 Hz), 118.8 (d, J = 3.6 Hz), 115.78 (d, J = 22.1 Hz), 79.0, 49.5 ppm.

(*E*)-1-(1,2-dibromovinyl)-4-fluorobenzene (3f):

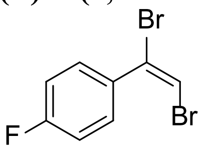

(*Z*)-1-(1,2-dibromovinyl)-4-fluorobenzene (4f):

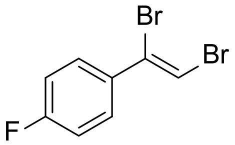

3f/4f: The crude product was purified by silica gel column chromatography with hexanes as the eluent to give **3f** as a yellow oil (46.2 mg, 55%) and **4f** as yellow oil (5 mg, 6%), respectively. **3f**: ^{1}H NMR (400 MHz, $CDCl_3$): δ = 7.52–7.49 (m, 2H), 7.10–7.05 (m, 2H), 6.80 ppm (s, 1H); $^{13}C\{^1H\}$ NMR (100 MHz, $CDCl_3$): δ = 162.9 (d, J = 248.9 Hz), 133.1 (d, J = 3.5 Hz), 131.3 (d, J = 8.5 Hz), 120.3, 115.5 (d, J = 21.9 Hz), 103.4 ppm. **4f**: ^{1}H NMR (400 MHz, $CDCl_3$): δ = 7.51–7.47 (m, 2H), 7.07–7.02 (m, 2H), 7.01 ppm (s, 1H); $^{13}C\{^1H\}$ NMR (100 MHz, $CDCl_3$): δ = 163.2 (d, J = 248.8 Hz), 134.7 (d, J = 3.3 Hz), 129.9, 129.6 (d, J = 8.4 Hz), 115.6 (d, J = 21.8 Hz), 108.7 ppm.

2,2-dibromo-1-(4-fluorophenyl)ethanone (5f):

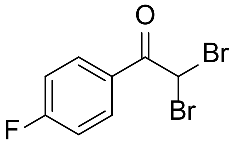

5f: The crude product was purified by silica gel column chromatography with 50:1 hexanes/EtOAc as the eluent to give a yellow oil (69.2 mg, 78%). ^{1}H NMR (400 MHz, $CDCl_3$): δ = 8.17–8.14 (m, 2H), 7.21–7.16 (m, 2H), 6.63 ppm (s, 1H); $^{13}C\{^1H\}$ NMR (100 MHz, $CDCl_3$): δ = 184.6, 166.4 (d, J = 256.4 Hz), 132.7(d, J = 9.6 Hz), 127.1 (d, J = 3.1 Hz), 116.3 (d, J = 22 Hz), 39.4 ppm.

1-fluoro-4-(1,1,2,2-tetrabromoethyl)benzene (6f):

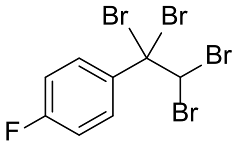

6f: The crude product was purified by silica gel column chromatography with 100:1 hexanes/EtOAc as the eluent to give a yellow oil (69.9 mg, 53%). ^{1}H NMR (400 MHz, CDCl$_3$): δ = 7.91–7.88 (m, 2H), 7.09–7.05 (m, 2H), 6.35 ppm (s, 1H); ^{13}C{^{1}H} NMR (100 MHz, CDCl$_3$): δ = 163.1 (d, J = 250.6 Hz), 135.6 (d, J = 3.1 Hz), 131.0 (d, J = 8.4 Hz), 115.1 (d, J = 21.9 Hz), 71.3, 54.6 ppm.

1-(bromoethynyl)-4-chlorobenzene (2g):

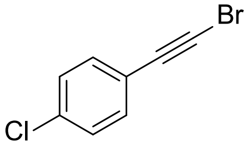

2g: The crude product was purified by silica gel column chromatography with hexanes as the eluent to give a white solid (64 mg, 99%). ^{1}H NMR (400 MHz, CDCl$_3$): δ = 7.37 (d, J = 8.8 Hz, 2H), 7.28 ppm (d, J = 8.4 Hz, 2H); ^{13}C{^{1}H} NMR (100 MHz, CDCl$_3$): δ = 134.8, 133.2, 128.7, 121.2, 79.0, 51.0 ppm.

(*E*)-1-chloro-4-(1,2-dibromovinyl)benzene (3g):

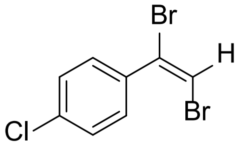

(*Z*)-1-chloro-4-(1,2-dibromovinyl)benzene (4g):

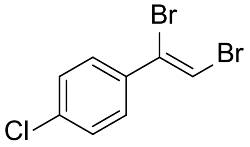

3g/4g: The crude product was purified by silica gel column chromatography with hexanes as the eluent to give **3g** as a white solid (39.1 mg, 44%) and **4g** as white solid (3.6 mg, 4%), respectively. **3g**: ^{1}H NMR (400 MHz, CDCl$_3$): δ = 7.45 (d, J = 8.4 Hz, 2H), 7.36 (d, J = 8.8 Hz, 2H), 6.81 ppm (s, 1H); ^{13}C NMR (100 MHz, CDCl$_3$): δ = 135.5, 135.4, 130.6, 128.6, 120.1, 103.8 ppm. **4g**: ^{1}H NMR (400 MHz, CDCl$_3$): δ = 7.44 (d, J = 8.4 Hz, 2H), 7.33 (d, J = 8.8 Hz, 2H), 7.06 ppm (s, 1H); ^{13}C{^{1}H} NMR (100 MHz, CDCl$_3$): δ = 136.9, 135.5, 129.9, 128.9,128.8, 109.5 ppm.

2,2-dibromo-1-(4-chlorophenyl)ethanone (5g):

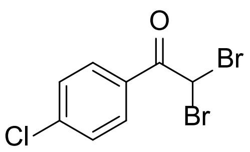

5g: The crude product was purified by silica gel column chromatography with 50:1 hexanes/EtOAc as the eluent to give a white solid (64.7 mg, 69%). ^{1}H NMR (400 MHz, CDCl$_3$): δ =8.05 (d, J = 8.8 Hz, 2H), 7.48 (d, J = 8.8 Hz, 2H), 6.62 ppm (s, 1H); ^{13}C{^{1}H} NMR (100 MHz, CDCl$_3$): δ =184.9, 141.1, 131.2, 129.3, 129.1, 39.3 ppm.

1-chloro-4-(1,1,2,2-tetrabromoethyl)benzene (6g):

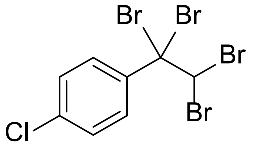

6g: The crude product was purified by silica gel column chromatography with 100:1 hexanes/EtOAc as the eluent to give a yellow oil (42.4 mg, 31%). ^{1}H NMR (400 MHz, CDCl$_3$): δ = 7.83 (d, J = 8.8 Hz, 2H), 7.36 (d, J = 8.8 Hz, 2H), 6.35 ppm (s, 1H); ^{13}C{^{1}H} NMR (100 MHz, CDCl$_3$): δ =138.3, 136.2, 130.2, 128.4, 71.1, 54.2 ppm.

bromo-4-(bromoethynyl)benzene (2h):

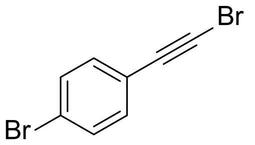

2h: The crude product was purified by silica gel column chromatography with hexanes as the eluent to give a white solid (60.8 mg, 78%). ^{1}H NMR (400 MHz, CDCl$_3$): δ = 7.45 (d, J = 8.8 Hz, 2H), 7.30 ppm (d, J = 8.4 Hz, 2H); ^{13}C{^{1}H} NMR (100 MHz, CDCl$_3$): δ = 133.4, 131.6, 123.0, 121.7, 79.1, 51.2 ppm.

(*E*)-1-bromo-4-(1,2-dibromovinyl)benzene (3h):

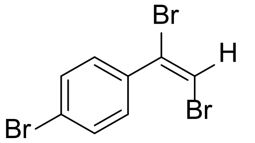

(*Z*)-1-bromo-4-(1,2-dibromovinyl)benzene (4h):

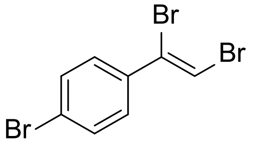

3h/4h: The crude product was purified by silica gel column chromatography with hexanes as the eluent to give **3h** as a light yellow solid (42.9 mg, 42%) and **4h** as light yellow solid (4.1 mg, 4%), respectively. 3h: ^{1}H NMR (400 MHz, CDCl$_3$): δ = 7.53 (d, J = 8.8 Hz, 2H), 7.38 (d, J = 8.4 Hz, 2H), 6.82 ppm (s, 1H); ^{13}C{^{1}H} NMR (100 MHz, CDCl$_3$): δ = 135.9, 131.6, 130.8, 123.7, 120.1, 103.8 ppm. **4h**: ^{1}H NMR (400 MHz, CDCl$_3$): δ = 7.49 (d, J = 8.8 Hz, 2H), 7.37 (d, J = 8.4 Hz, 2H), 7.07 (s, 1H); ^{13}C{^{1}H} NMR (100 MHz, CDCl$_3$): δ = 137.4, 131.8, 129.9, 129.2, 123.7, 109.5 ppm.

2,2-dibromo-1-(4-bromophenyl)ethanone (5h):

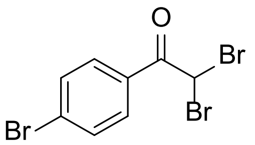

5h: The crude product was purified by silica gel column chromatography with 50:1 hexanes/EtOAc as the eluent to give a light yellow solid (57.8 mg, 54%). ^{1}H NMR (400 MHz, CDCl$_3$): δ = 7.97 (d, J = 8.8 Hz, 2H), 7.66 (d, J = 8.8 Hz, 2H), 6.59 ppm (s, 1H); ^{13}C{^{1}H} NMR (100 MHz, CDCl$_3$): δ = 185.1, 132.3, 131.2, 129.9, 129.5 39.2 ppm.

1-bromo-4-(1,1,2,2-tetrabromoethyl)benzene (6h):

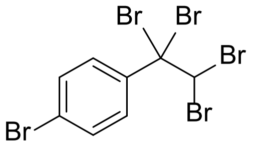

6h: The crude product was purified by silica gel column chromatography with 100:1 hexanes/EtOAc as the eluent to give a yellow oil (33 mg, 22%). ^{1}H NMR (400 MHz, CDCl$_3$): δ = 7.76 (d, J = 8.8 Hz, 2H), 7.51 (d, J = 9.2 Hz, 2H), 6.35 ppm (s, 1H); ^{13}C{^{1}H} NMR (100 MHz, CDCl$_3$): δ = 138.8, 131.4, 130.4, 124.5, 71.2, 54.0 ppm.

1-(bromoethynyl)-4-(trifluoromethyl)benzene (2i):

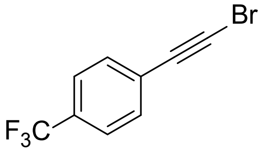

2i: The crude product was purified by silica gel column chromatography with hexanes as the eluent to give a brown solid (74 mg, 99%). ^{1}H NMR (400 MHz, CDCl$_3$): δ = 7.59–7.53 ppm (m, 4H); ^{13}C{^{1}H} NMR (100 MHz, CDCl$_3$): δ = 137.5, 132.3, 130.5 (q, J = 32.5 Hz), 125.3 (q, J = 32.5 Hz), 125.2 (q, J = 270.6 Hz), 78.8, 53.0 ppm.

(*E*)-1-(1,2-dibromovinyl)-4-(trifluoromethyl)benzene (3i):

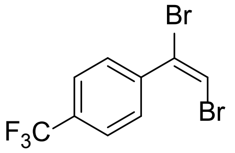

(*Z*)-1-(1,2-dibromovinyl)-4-(trifluoromethyl)benzene (4i):

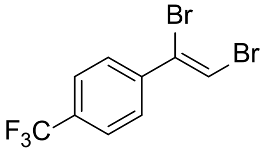

3i/4i: The crude product was purified by silica gel column chromatography with hexanes as the eluent to give **3i** as a brown solid (53.5 mg, 54%) and **4i** as brown solid (10.9 mg, 11%), respectively. **3i**: ^{1}H NMR (400 MHz, CDCl$_3$): δ = 7.67–7.61 (m, 4H), 6.89 (s, 1H); ^{13}C{^{1}H} NMR (100 MHz, CDCl$_3$): δ = 140.6, 131(q, J = 32.7 Hz), 129.6, 126.4(q, J = 270.6 Hz), 125.4 (q, J = 3.7 Hz), 119.4, 104.7. **4i**: ^{1}H NMR (400 MHz, CDCl$_3$): δ = 7.66 (s, 4H), 7.17 ppm (s, 1H); ^{13}C{^{1}H} NMR (100 MHz, CDCl$_3$): δ = 141.7, 131.3 (q, J = 32.6 Hz), 129.5, 126.4 (q, J = 270.6 Hz), 125.6 (q, J = 3.8 Hz), 119.7, 111.1 ppm.

2,2-dibromo-1-(4-(trifluoromethyl)phenyl)ethanone (5i):

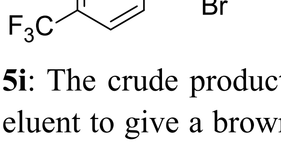

5i: The crude product was purified by silica gel column chromatography with 50:1 hexanes/EtOAc as the eluent to give a brownish yellow solid (9.3 mg, 9%). ^{1}H NMR (400 MHz, CDCl$_3$): δ = 8.23 (d, J = 8.4 Hz, 2H), 7.78 (d, J = 8.4 Hz, 2H), 6.62 ppm (s, 1H); ^{13}C{^{1}H} NMR (100 MHz, CDCl$_3$): δ = 185.0, 135.5 (q, J = 32.8 Hz), 133.7, 130.2, 125.9 (q, J = 3.7 Hz), 126.0 (q, J = 270 Hz), 39.0 ppm.

1-(1,1,2,2-tetrabromoethyl)-4-(trifluoromethyl)benzene (6i):

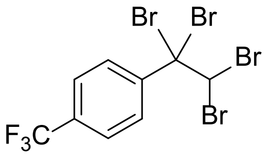

6i: The crude product was purified by silica gel column chromatography with 100:1 hexanes/EtOAc as the eluent to give **6i** as a yellow solid (20.6 mg, 14%). Mp: 63–65 °C; ^{1}H NMR (400 MHz, $CDCl_3$): δ = 8.03 (d, J = 8.4 Hz, 2H), 7.65 (d, J = 8.4 Hz, 2H), 6.40 ppm (s, 1H); $^{13}C\{^{1}H\}$ NMR (100 MHz, $CDCl_3$): δ = 143.3, 131.9 (q, J = 32.8 Hz), 129.3, 125.3 (q, J = 3.7 Hz), 123.5 (q, J = 270.8 Hz), 70.4, 53.6 ppm. IR(KBr): 2987, 2920, 2830, 1617, 1408, 1328, 1157, 1136, 1070, 1015, 842, 760, 726, 701, 643, 621 cm^{-1}.

1-(bromoethynyl)-4-nitrobenzene (2j):

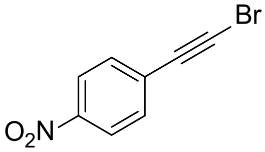

2j: The crude product was purified by silica gel column chromatography with 50:1 hexanes/EtOAc as the eluent to give a brown solid (57.6 mg, 85%). ^{1}H NMR (400 MHz, $CDCl_3$): δ = 8.19 (d, J = 8.8 Hz, 2H), 7.59 ppm (d, J = 8.8 Hz, 2H); $^{13}C\{^{1}H\}$ NMR (100 MHz, $CDCl_3$): δ = 146.4, 131.8, 128.5, 122.6, 77.4, 55.3 ppm.

(*E*)-1-(1,2-dibromovinyl)-4-nitrobenzene (3j):

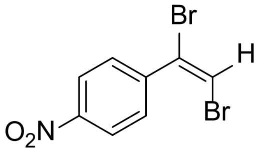

(*Z*)-1-(1,2-dibromovinyl)-4-nitrobenzene (4j):

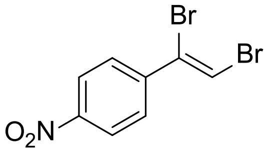

3j/4j: The crude product was purified by silica gel column chromatography with 50:1 hexanes/EtOAc as the eluent to give **3j** as a brown solid (35.9 mg, 39%) and **4j** as brown solid (9.2 mg, 10%), respectively. **3j**: ^{1}H NMR (400 MHz, $CDCl_3$): δ = 8.26 (d, J = 8.8 Hz, 2H), 7.69 (d, J = 8.8 Hz, 2H), 6.95 ppm (s, 1H); $^{13}C\{^{1}H\}$ NMR (100 MHz, $CDCl_3$): δ = 146.9, 142.3, 129.3, 122.6, 117.4, 104.8 ppm. **4j**: ^{1}H NMR (400 MHz, $CDCl_3$): δ = 8.22 (d, J = 8.8 Hz, 2H), 7.69 (d, J = 9.2 Hz, 2H), 7.29 ppm (s, 1H); $^{13}C\{^{1}H\}$ NMR (100 MHz, $CDCl_3$): δ = 148.1, 144.1, 128.7, 128.5, 123.9, 112.8 ppm.

2,2-dibromo-1-(4-nitrophenyl)ethanone (5j):

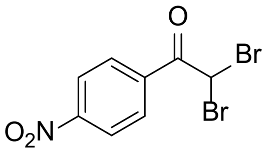

5j: The crude product was purified by silica gel column chromatography with 20:1 hexanes/EtOAc as the eluent to give **5j** as brown oil (2 mg, 2%). ^{1}H NMR (400 MHz, $CDCl_3$): δ = 8.25 (d, J = 8.8 Hz, 2H), 7.75 (d, J = 8.8 Hz, 2H), 6.67 ppm (s, 1H); $^{13}C\{^{1}H\}$ NMR (100 MHz, $CDCl_3$): δ = 201.7, 145.4, 128.3, 126.7, 123.0, 53.6 ppm.

1-nitro-4-(1,1,2,2-tetrabromoethyl)benzene (6j):

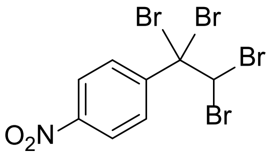

6j: The crude product was purified by silica gel column chromatography with 30:1 hexanes/EtOAc as the eluent to give **6j** as brown solid (25.2 mg, 18%). Mp: 77–79 °C; ^{1}H NMR (400 MHz, $CDCl_3$): δ = 8.25 (d, J = 9.2 Hz, 2H), 8.10 (d, J = 8.8 Hz, 2H), 6.40 ppm (s, 1H); $^{13}C\{^{1}H\}$ NMR (100 MHz, $CDCl_3$): δ = 147.3, 129.1, 123.0, 122.3, 82.7, 52.0 ppm; IR(KBr): 3103, 3002, 2961, 2924, 2850, 1600, 1513, 1485, 1400, 1346, 1318, 1261, 1166, 1136, 1110, 1014, 867, 845, 815, 765, 752, 705, 676, 608, 570 cm^{-1}.

4. NMR Spectra of products

NMR spectra of 1-(bromoethynyl)-4-methylbenzene (2a):

^{1}H NMR (400 MHz, CDCl$_3$) spectrum of **2a**

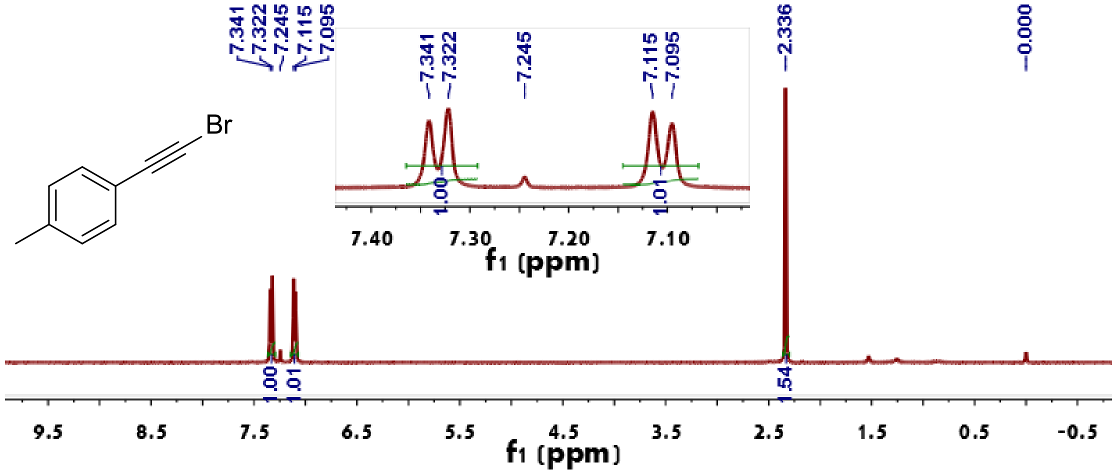

^{13}C{^{1}H} NMR (100 MHz, CDCl$_3$) spectrum of **2a**

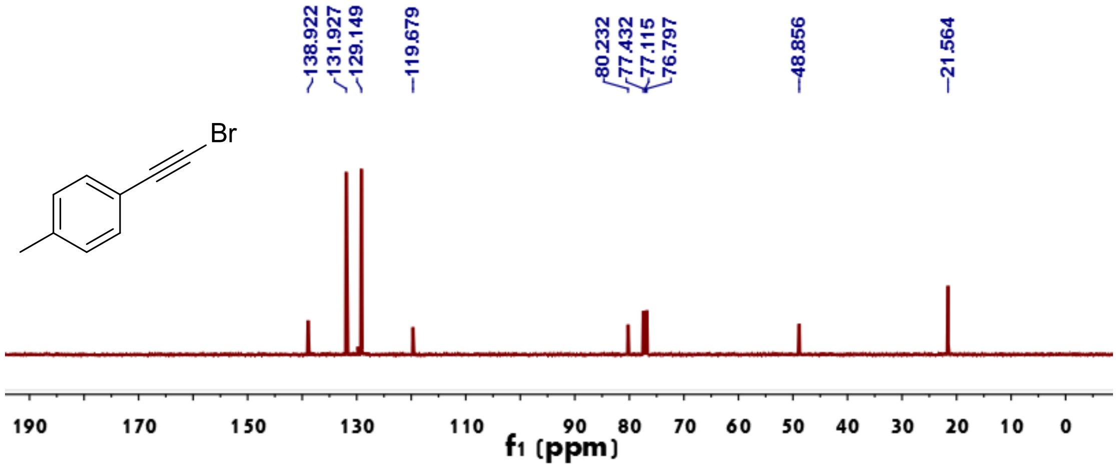

NMR spectra of (*E*)-1-(1,2-dibromovinyl)-4-methylbenzene (3a):

^{1}H NMR (400 MHz, $CDCl_3$) spectrum of **3a**

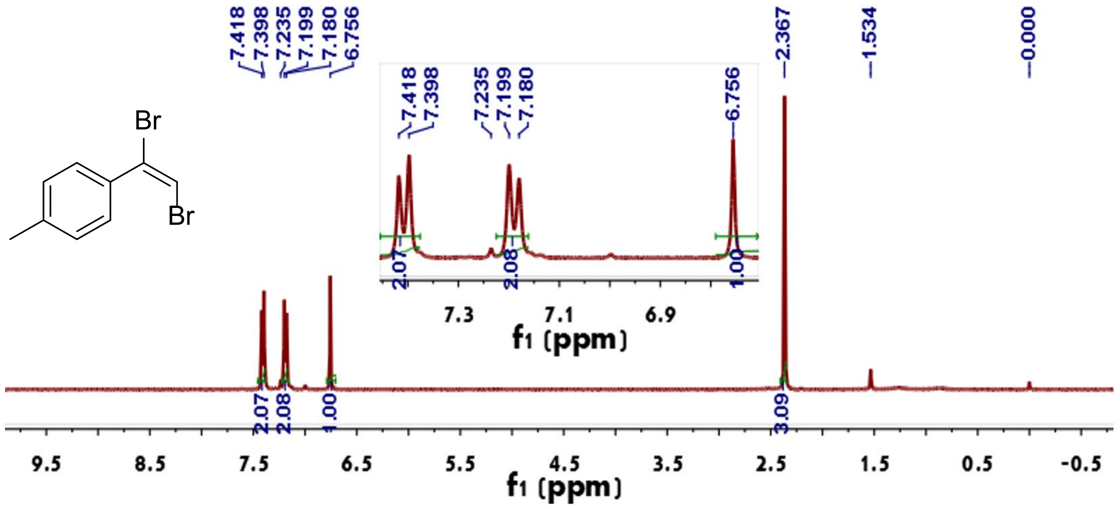

^{13}C{^{1}H} NMR (100 MHz, $CDCl_3$) spectrum of **3a**

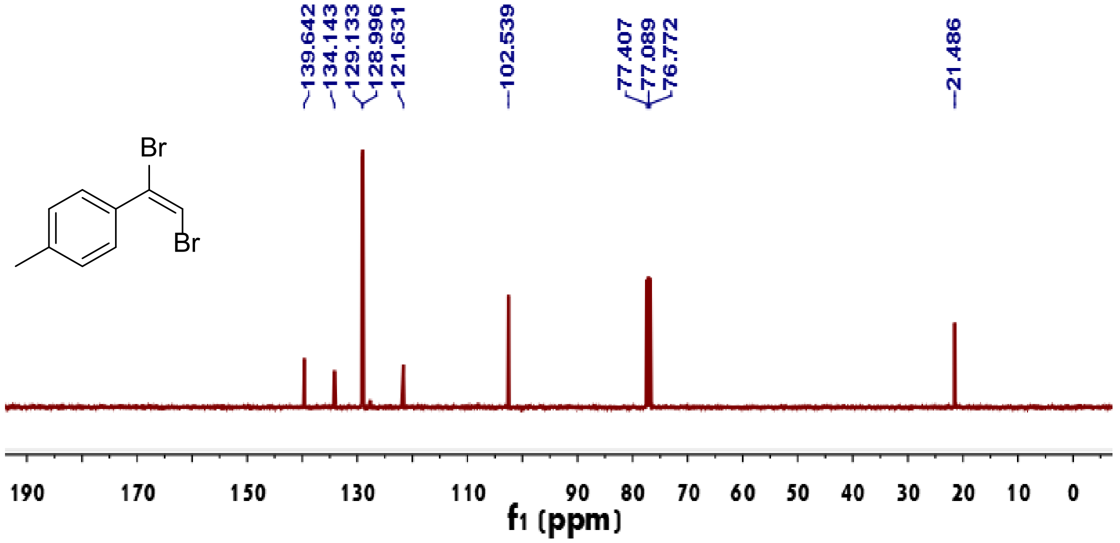

NMR spectra of (*Z*)-1-(1,2-dibromovinyl)-4-methylbenzene (4a):

^{1}H NMR (400 MHz, $CDCl_3$) spectrum of **4a**

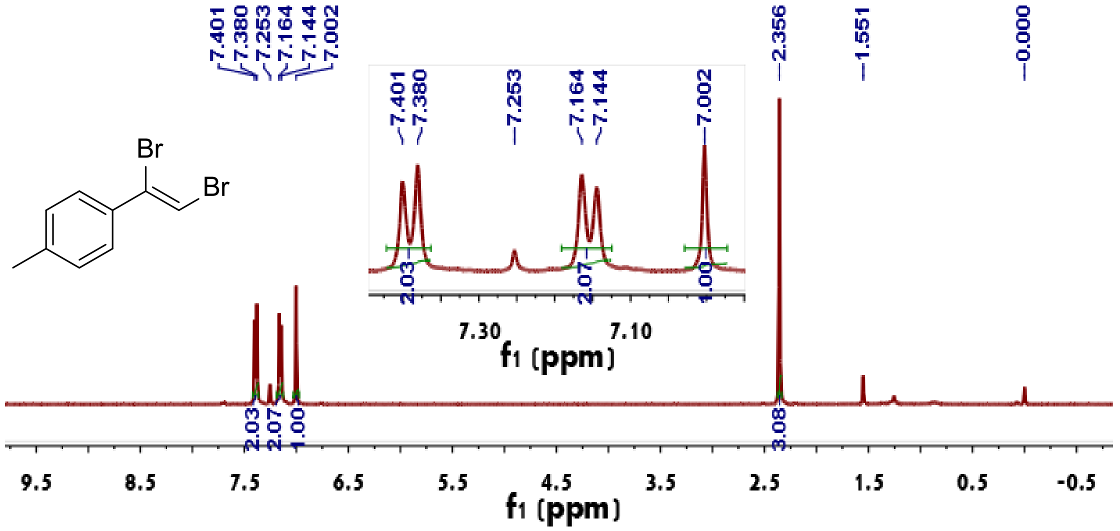

^{13}C{^{1}H} NMR (100 MHz, $CDCl_3$) spectrum of **4a**

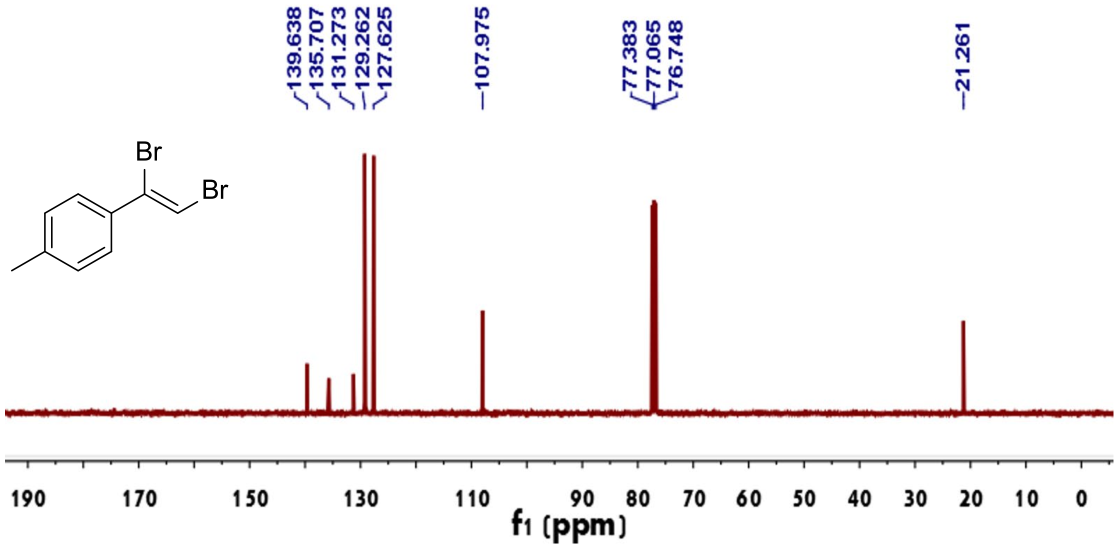

NMR Spectra of 2,2-dibromo-1-(p-tolyl)ethanone (5a):

^{1}H NMR (400 MHz, $CDCl_3$) spectrum of **5a**

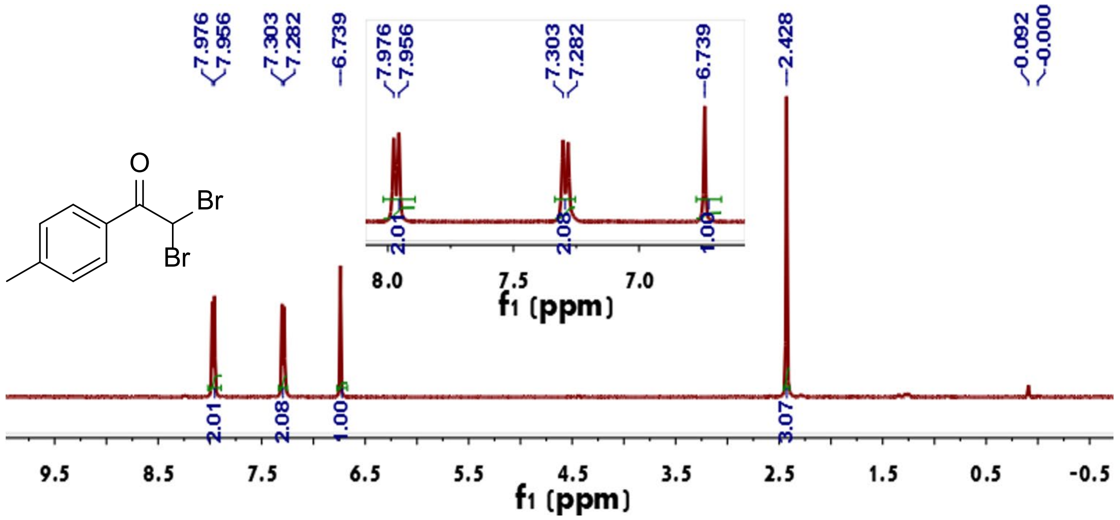

^{13}C{^{1}H} NMR (100 MHz, $CDCl_3$) spectrum of **5a**

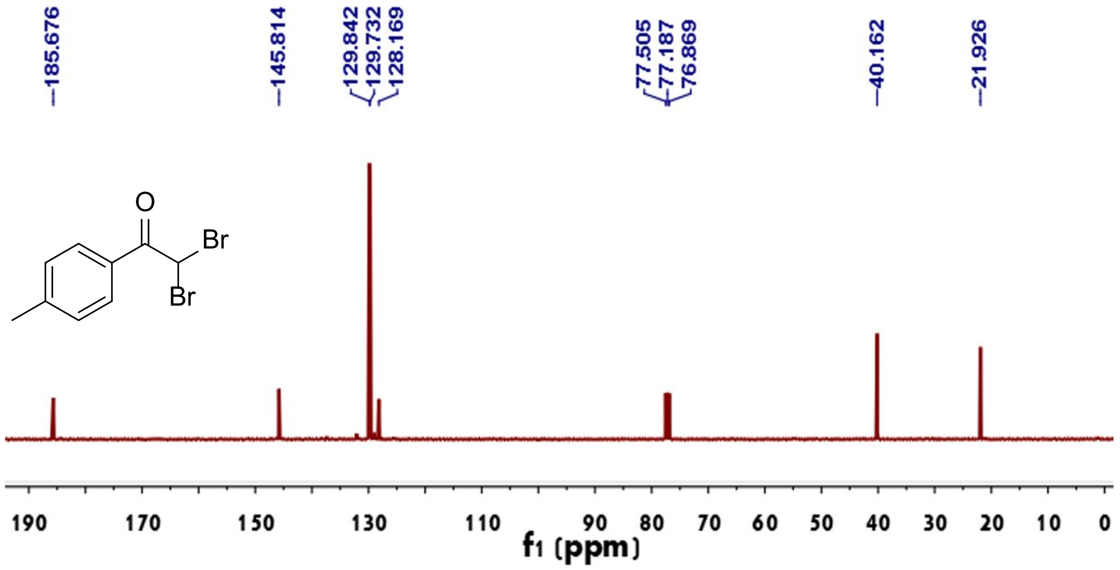

NMR Spectra of 1-methyl-4-(1,1,2,2-tetrabromoethyl)benzene (6a):

^{1}H NMR (400 MHz, $CDCl_3$) spectrum of **6a**

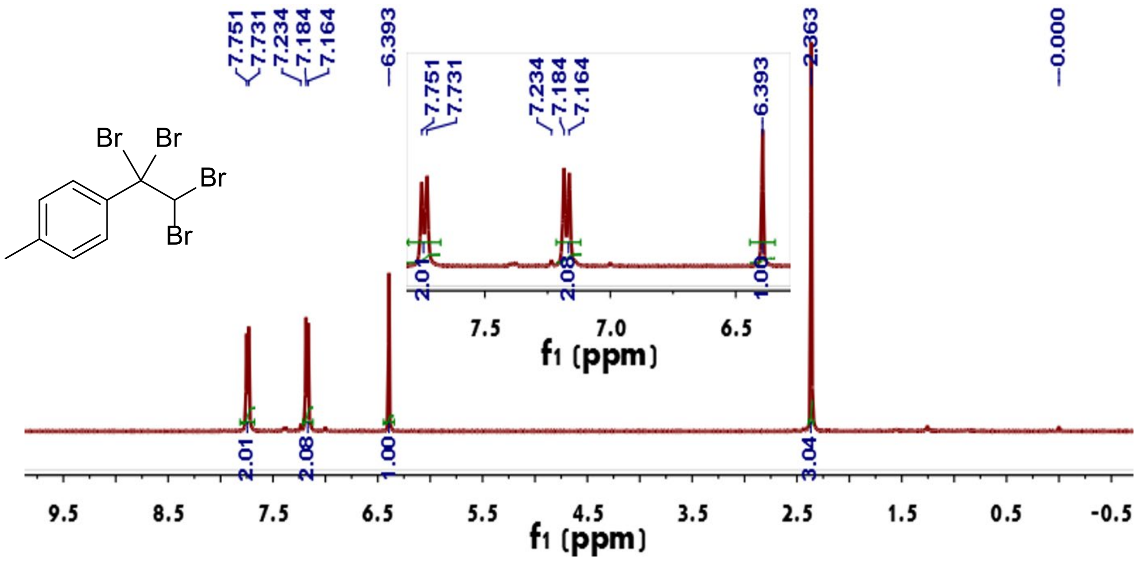

^{13}C{^{1}H} NMR (100 MHz, $CDCl_3$) spectrum of **6a**

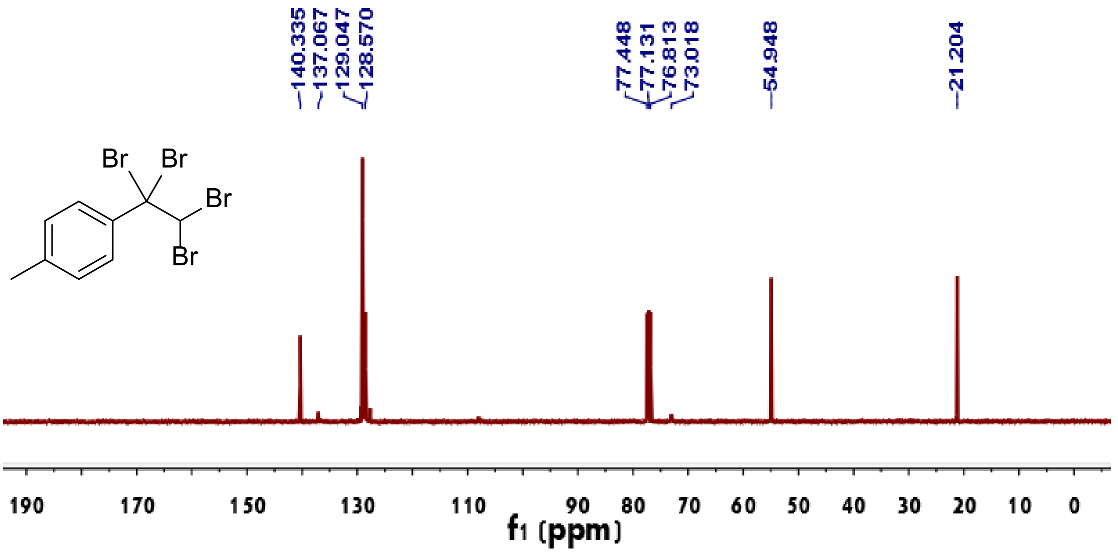

NMR Spectra of 1-(bromoethynyl)-3-methylbenzene (2b):

^{1}H NMR (400 MHz, $CDCl_3$) spectrum of **2b**

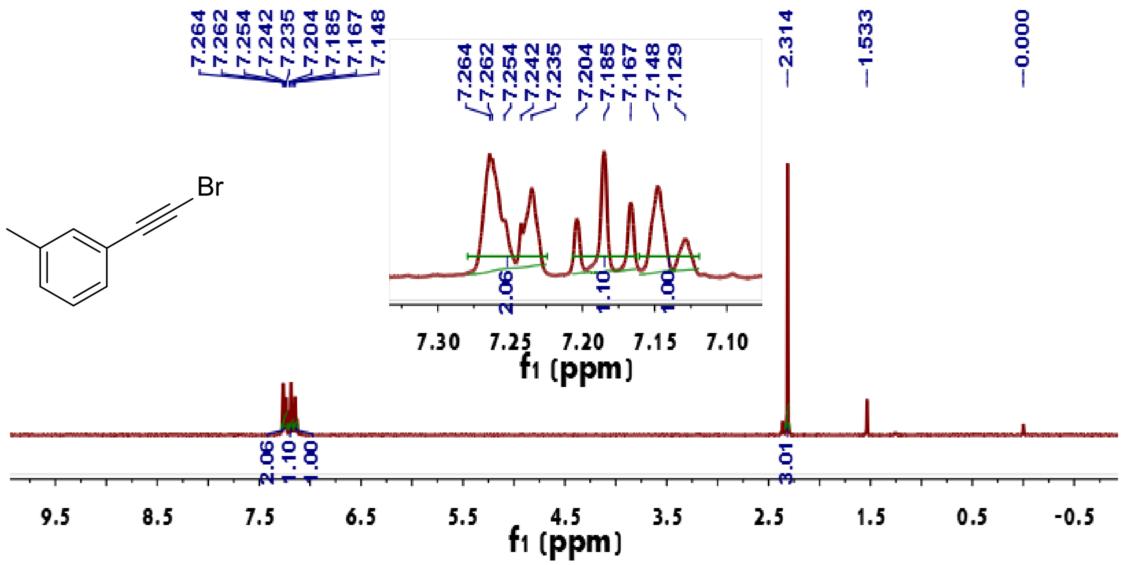

^{13}C{^{1}H} NMR (100 MHz, $CDCl_3$) spectrum of **2b**

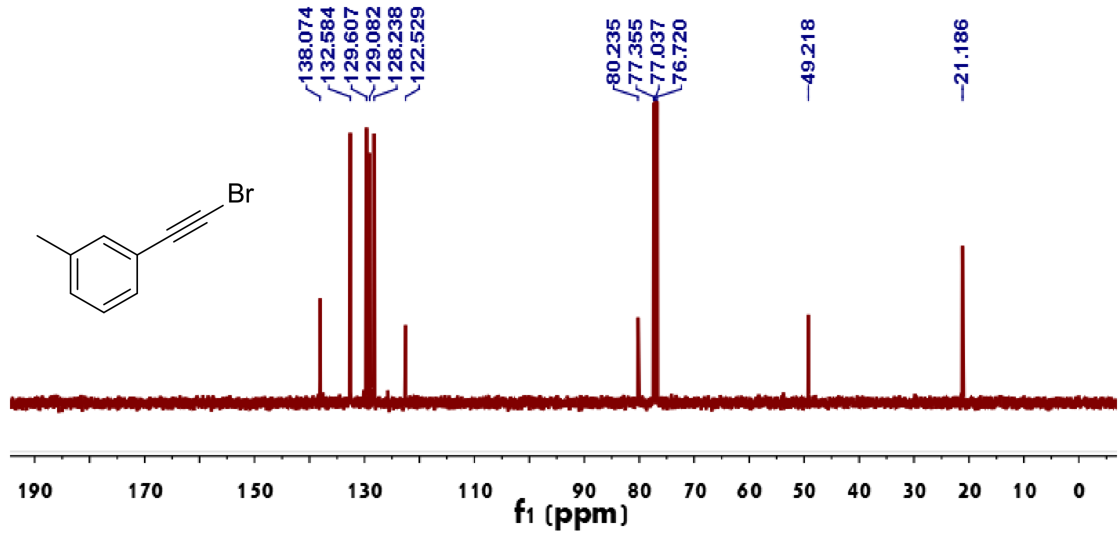

NMR Spectra of (*E*)-1-(1,2-dibromovinyl)-3-methylbenzene (3b):

^{1}H NMR (400 MHz, $CDCl_3$) spectrum of **3b**

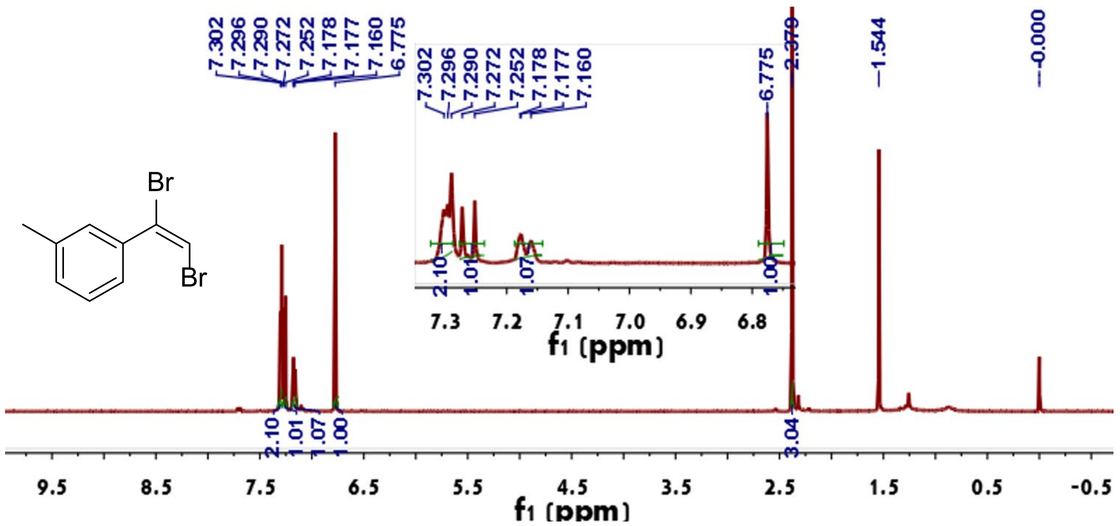

$^{13}C\{^1H\}$ NMR (100 MHz, $CDCl_3$) spectrum of **3b**

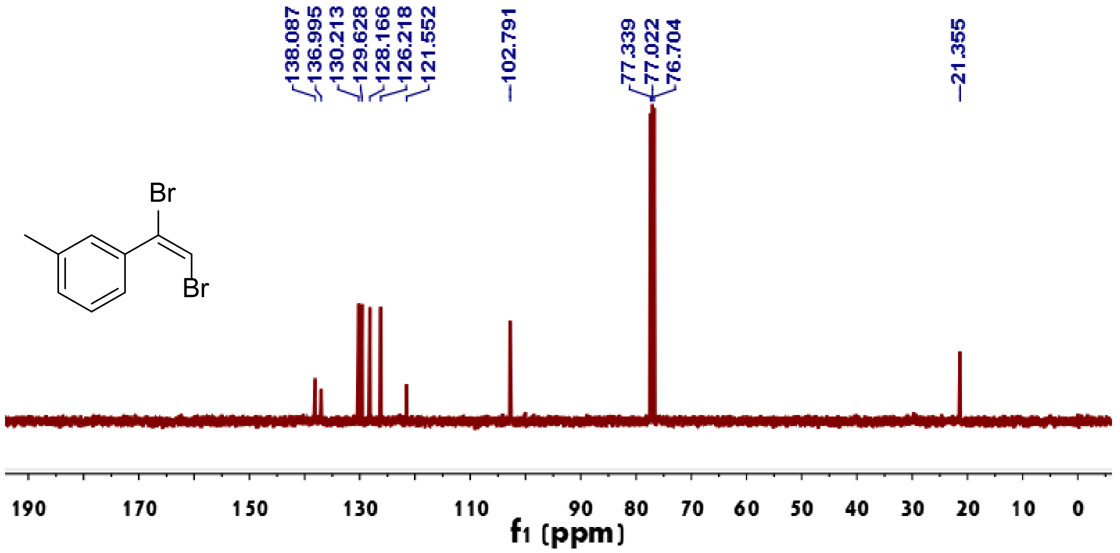

NMR Spectra of (*Z*)-1-(1,2-dibromovinyl)-3-methylbenzene (4b):

^{1}H NMR (400 MHz, $CDCl_3$) spectrum of **4b**

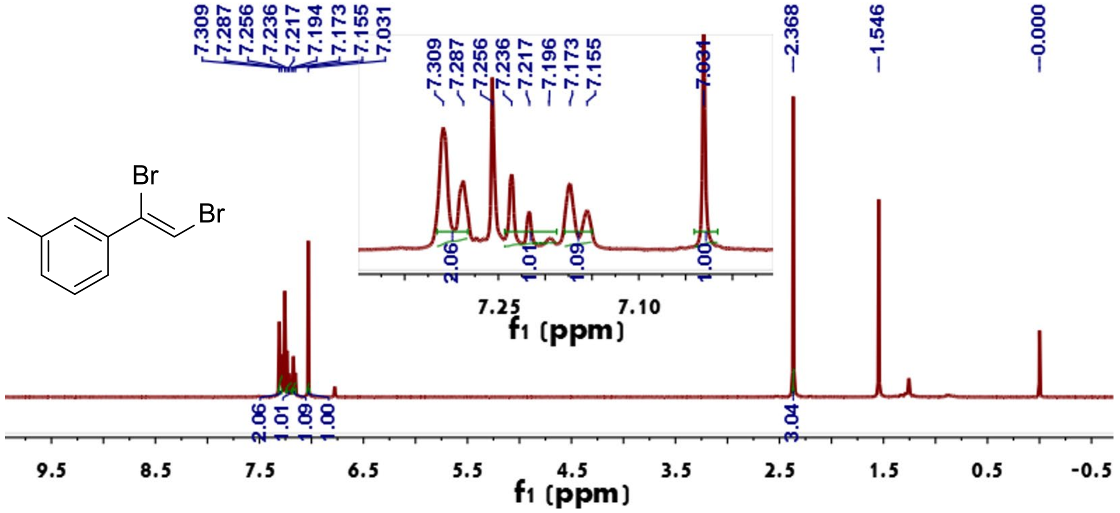

$^{13}C\{^{1}H\}$ NMR (100 MHz, $CDCl_3$) spectrum of **4b**

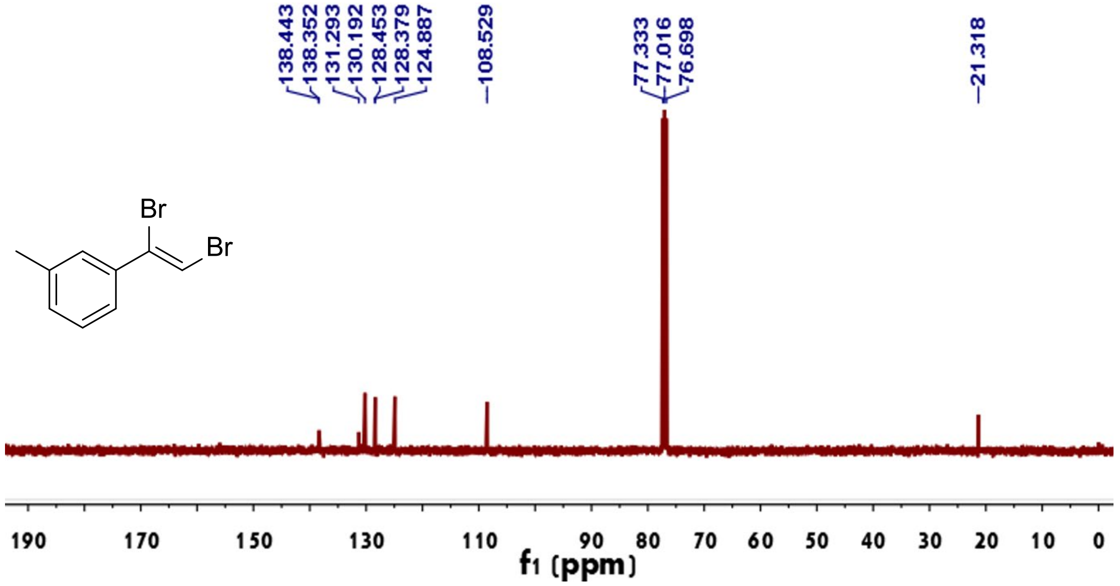

NMR Spectra of (*Z*)-1-(1,2-dibromovinyl)-3-methylbenzene (5b):

^{1}H NMR (400 MHz, $CDCl_3$) spectrum of **5b**

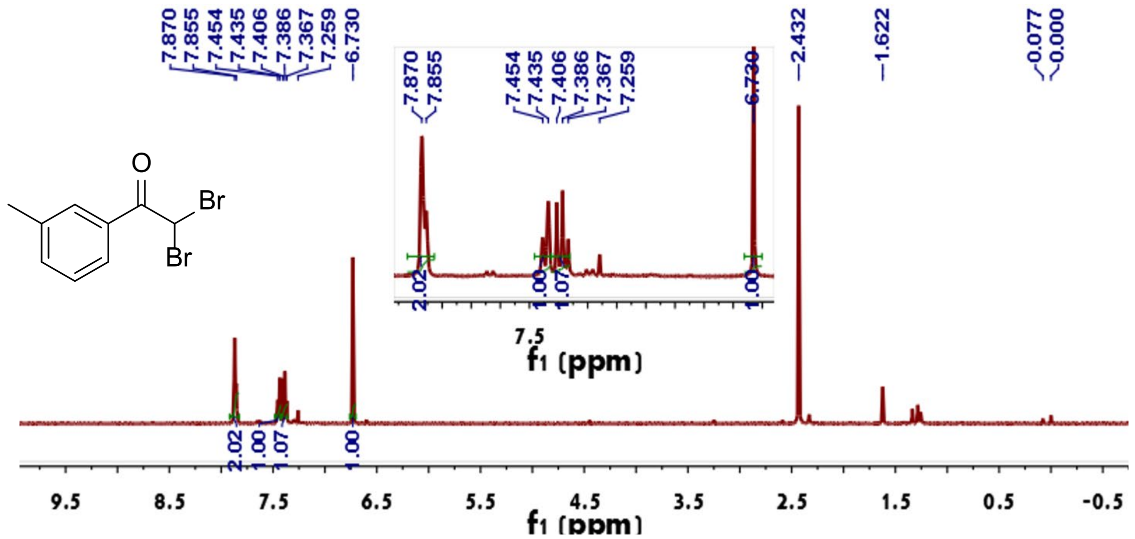

$^{13}C\{^{1}H\}$ NMR (100 MHz, $CDCl_3$) spectrum of **5b**

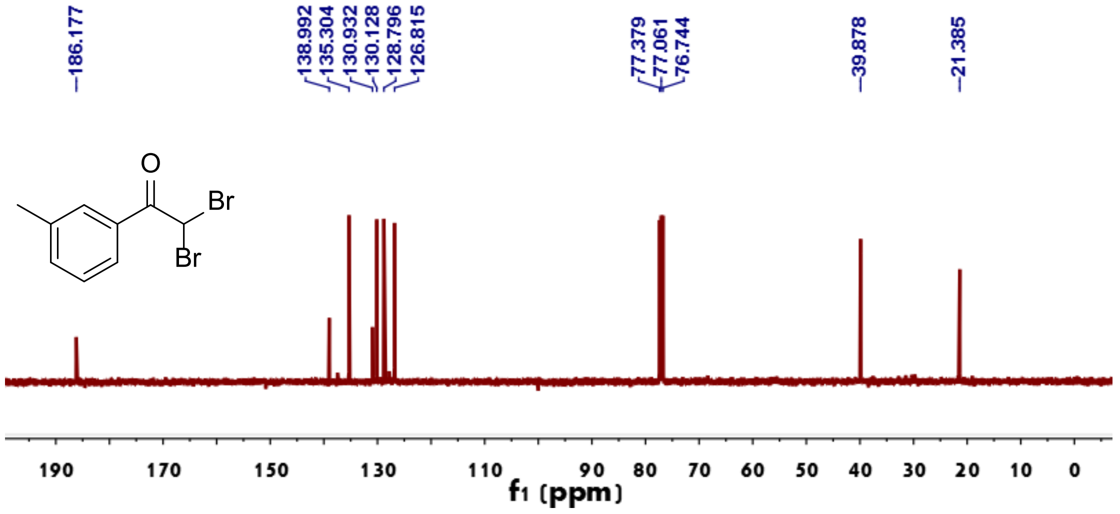

NMR Spectra of 1-methyl-3-(1,1,2,2-tetrabromoethyl)benzene (6b):

^{1}H NMR (400 MHz, $CDCl_3$) spectrum of **6b**

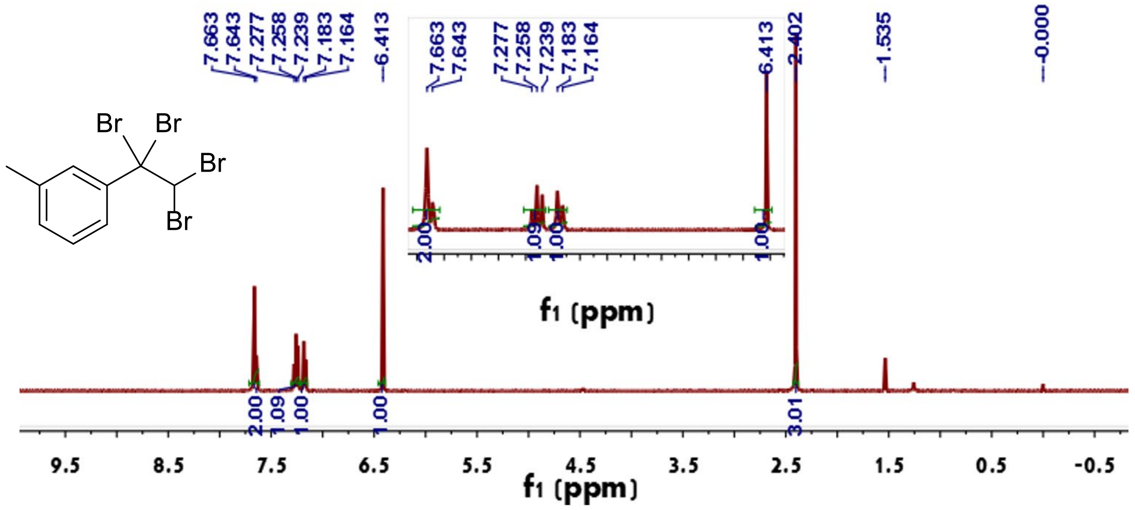

$^{13}C\{^{1}H\}$ NMR (100 MHz, $CDCl_3$) spectrum of **6b**

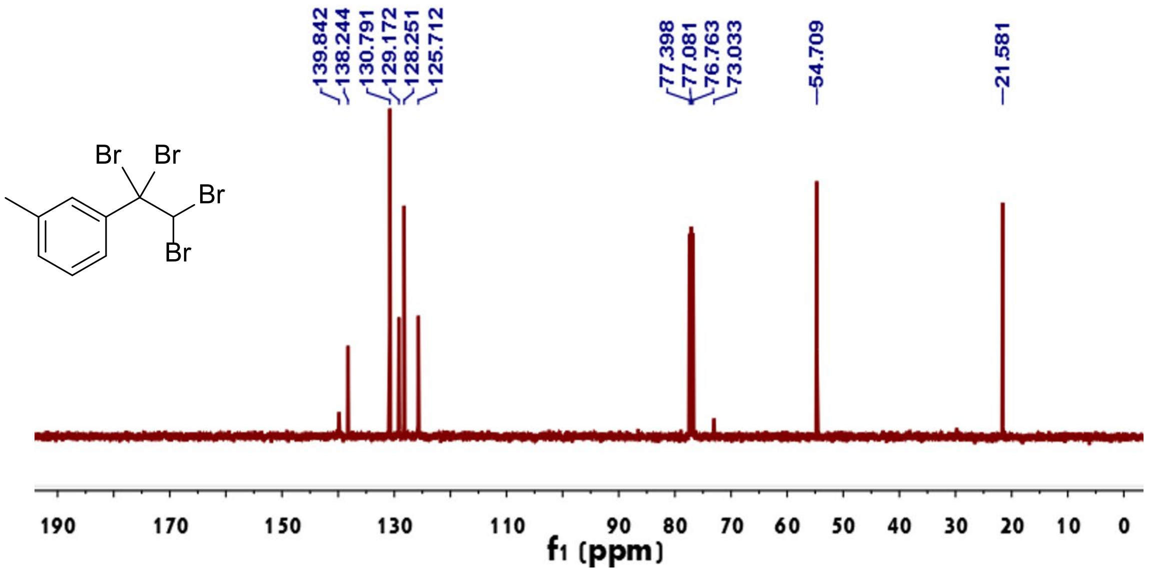

NMR Spectra of (bromoethynyl)benzene (2c):

^{1}H NMR (400 MHz, $CDCl_3$) spectrum of **2c**

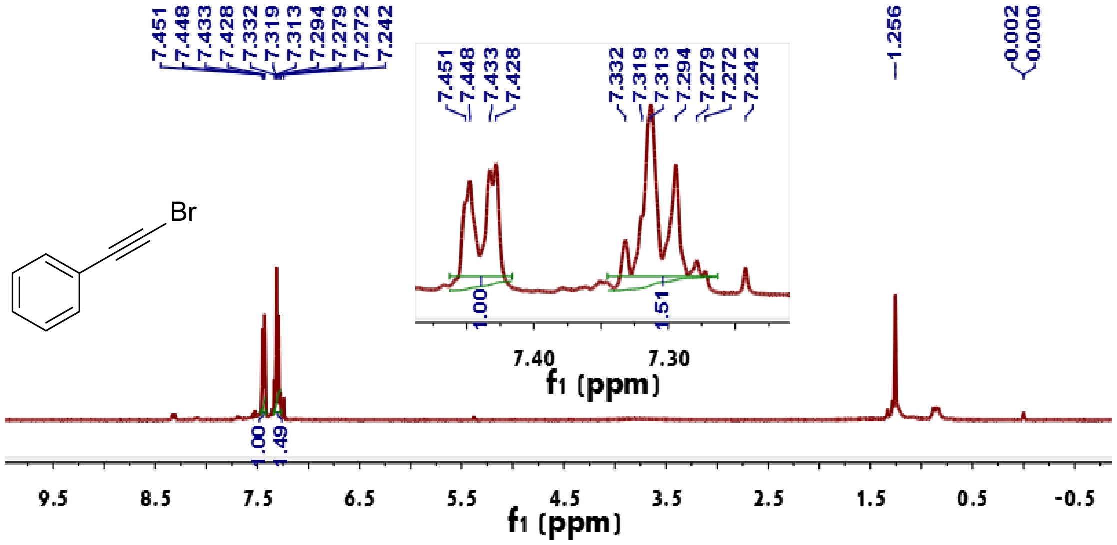

^{13}C{^{1}H} NMR (100 MHz, $CDCl_3$) spectrum of **2c**

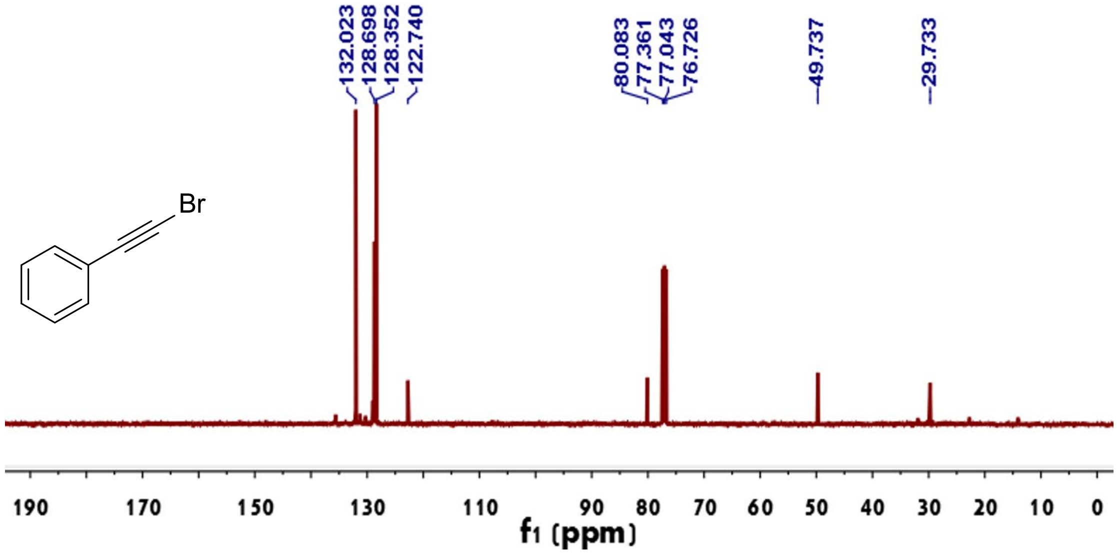

NMR Spectra of (*E*)-(1,2-dibromovinyl)benzene (3c):

^{1}H NMR (400 MHz, $CDCl_3$) spectrum of **3c**

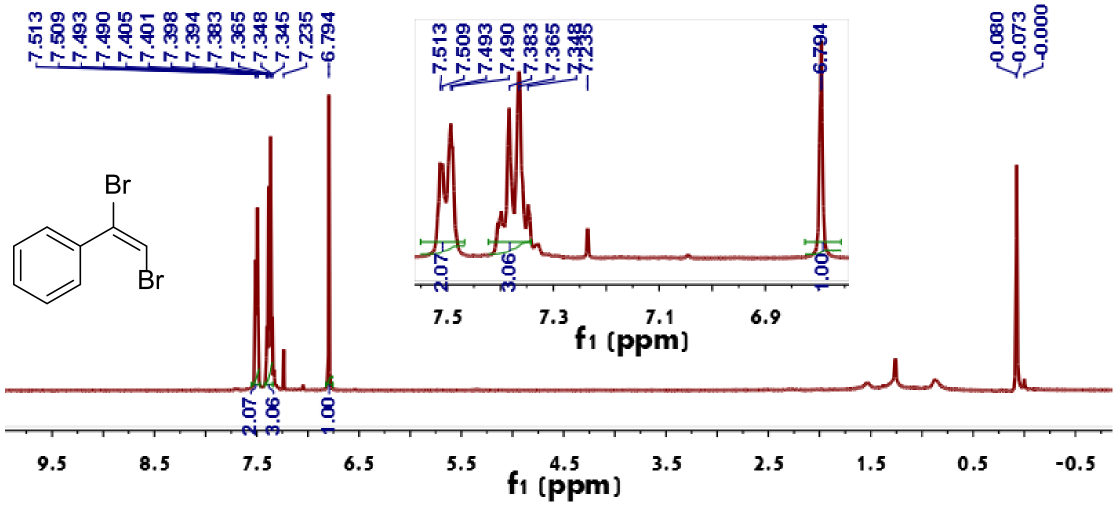

^{13}C{^{1}H} NMR (100 MHz, $CDCl_3$) spectrum of **3c**

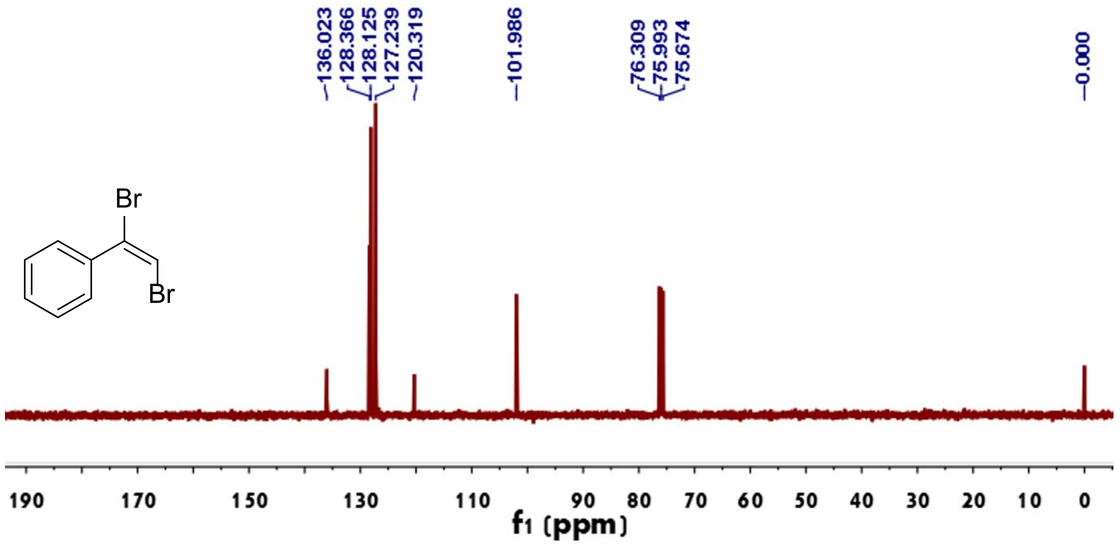

NMR Spectra of (*Z*)-(1,2-dibromovinyl)benzene (4c):

^{1}H NMR (400 MHz, $CDCl_3$) spectrum of **4c**

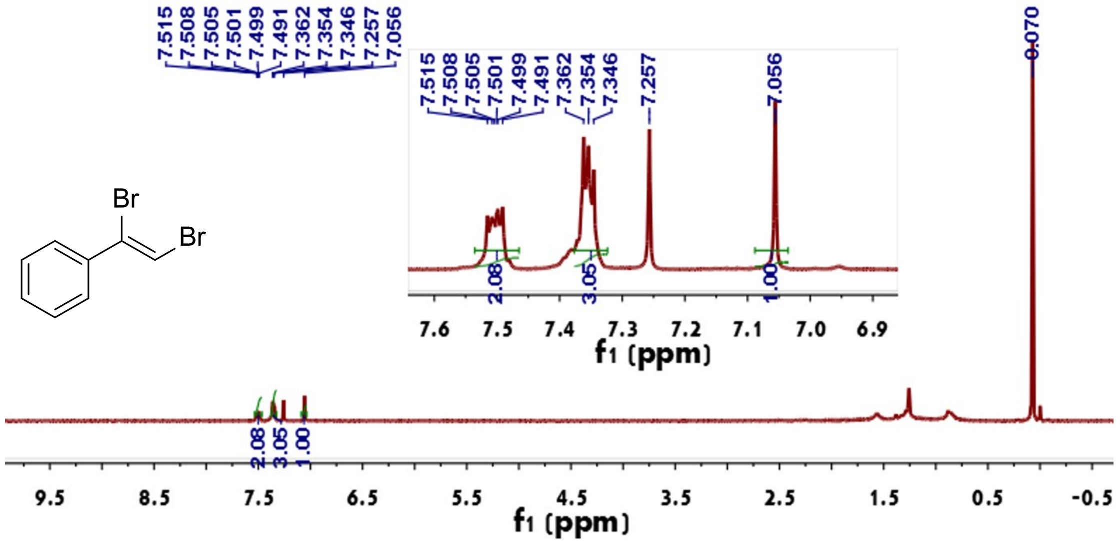

$^{13}C\{^1H\}$ NMR (100 MHz, $CDCl_3$) spectrum of **4c**

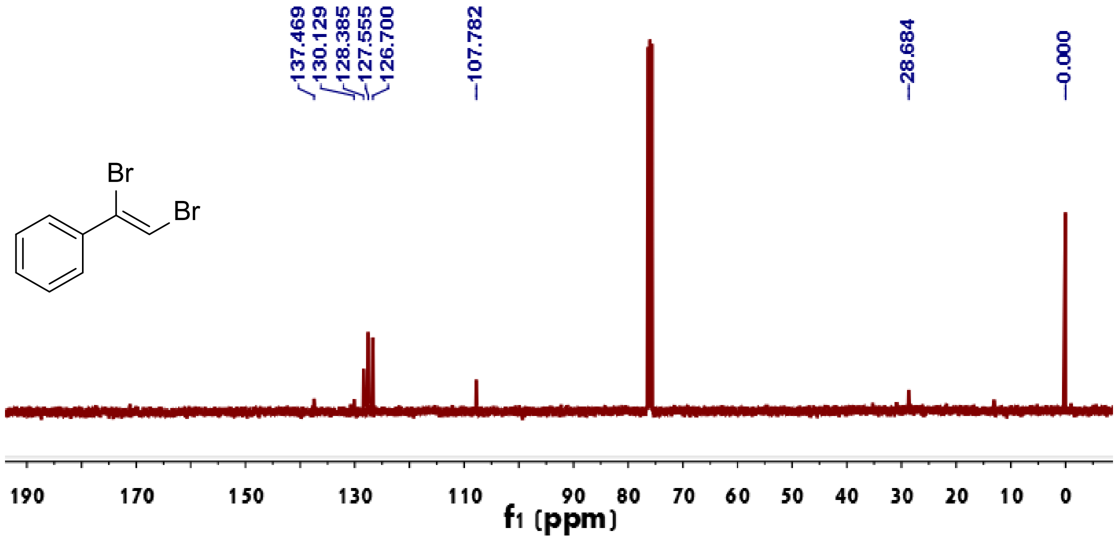

NMR Spectra of 2,2-dibromo-1-phenylethanone (5c):

^{1}H NMR (400 MHz, $CDCl_3$) spectrum of **5c**

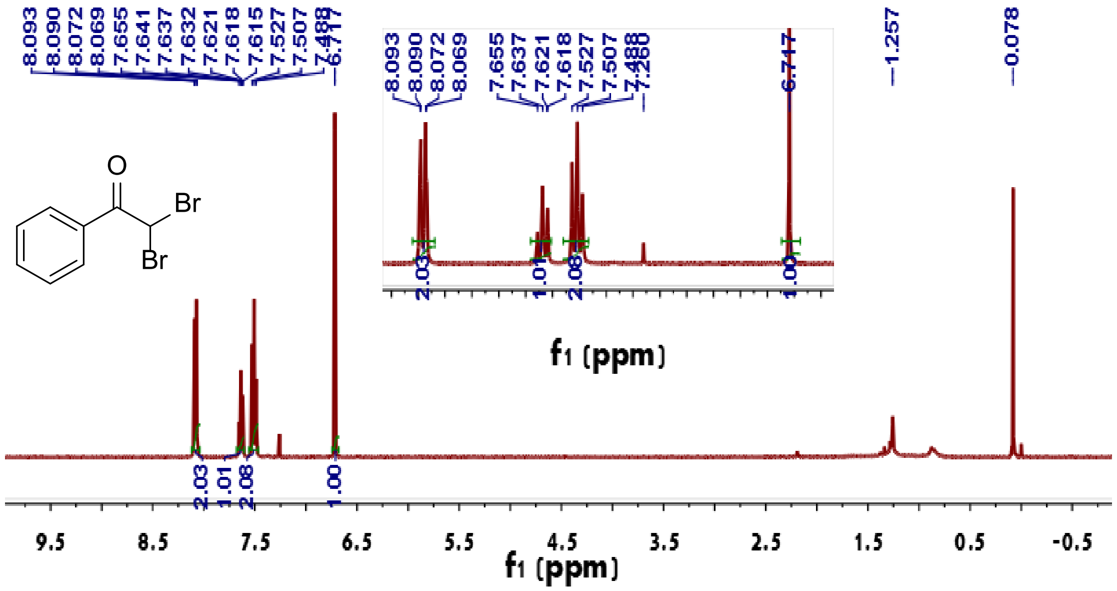

^{13}C{^{1}H} NMR (100 MHz, $CDCl_3$) spectrum of **5c**

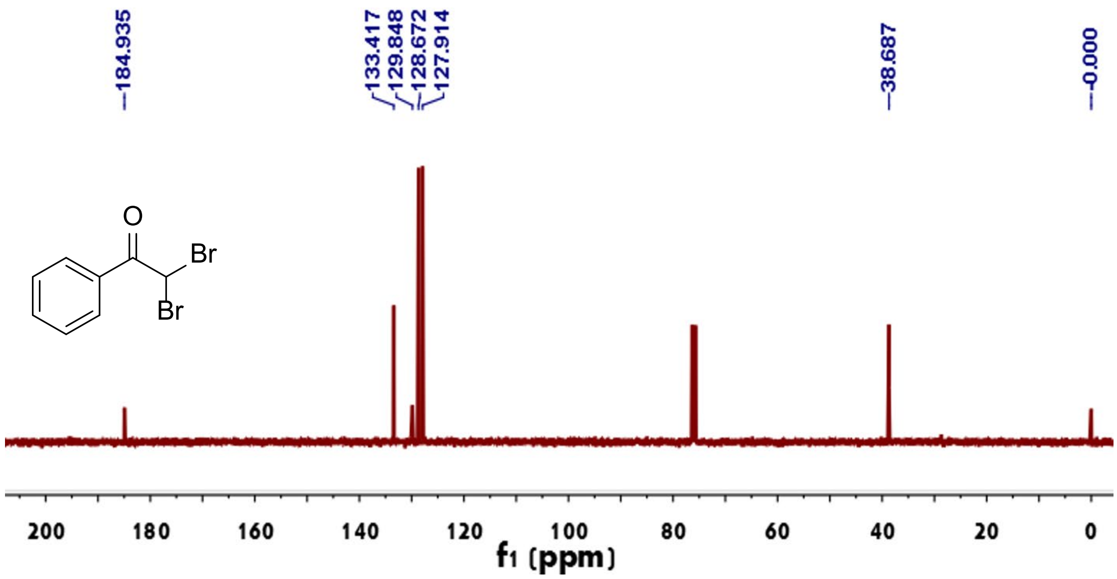

NMR Spectra of (1,1,2,2-tetrabromoethyl)benzene (6c):

^{1}H NMR (500 MHz, $CDCl_3$) spectrum of **6c**

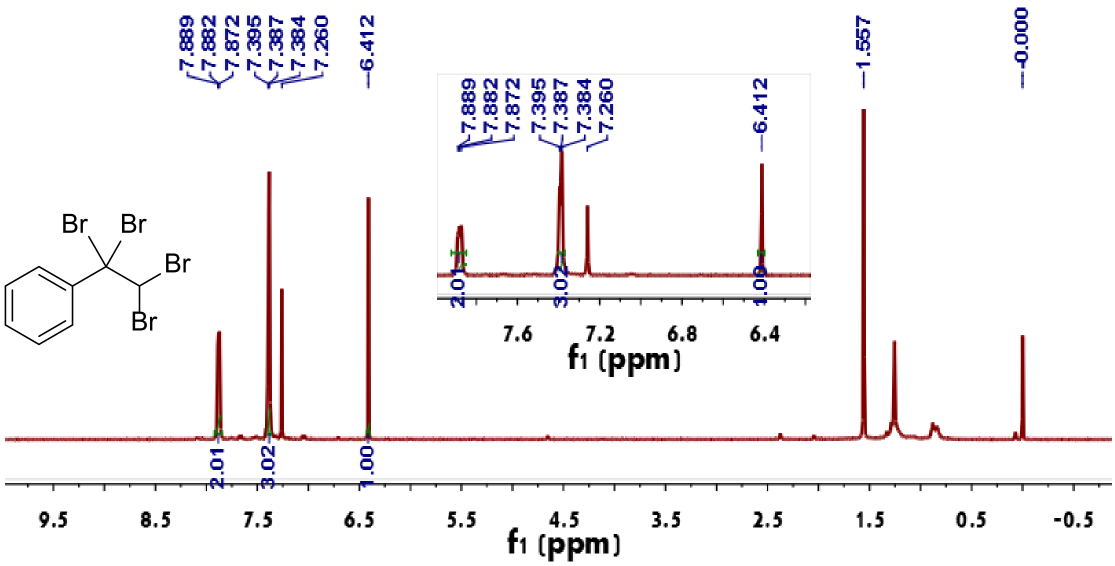

$^{13}C\{^{1}H\}$ NMR (125 MHz, $CDCl_3$) spectrum of **6c**

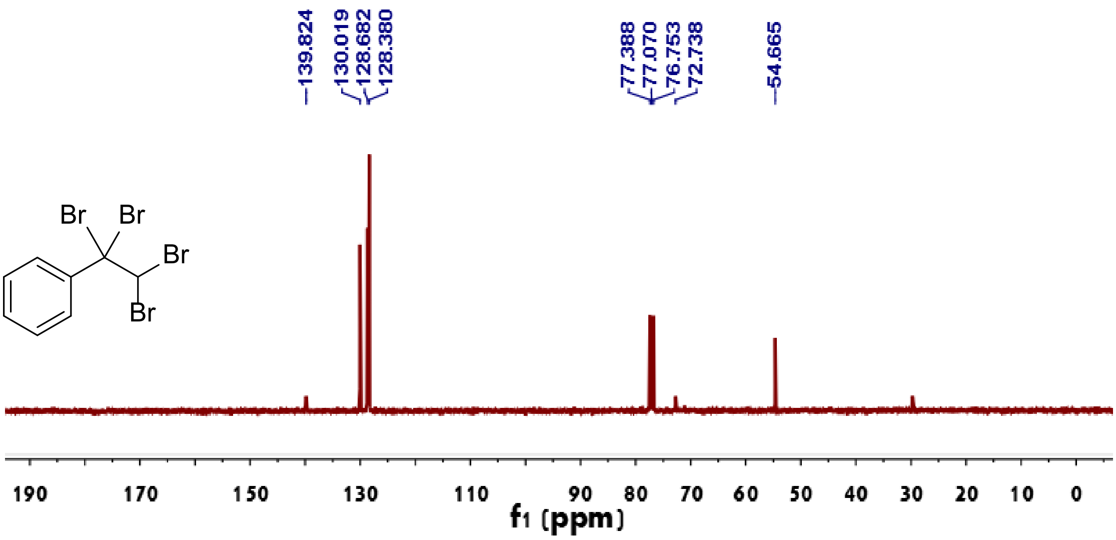

NMR Spectra of 1-(bromoethynyl)-4-methoxybenzene (2d):

^{1}H NMR (400 MHz, $CDCl_3$) spectrum of **2d**

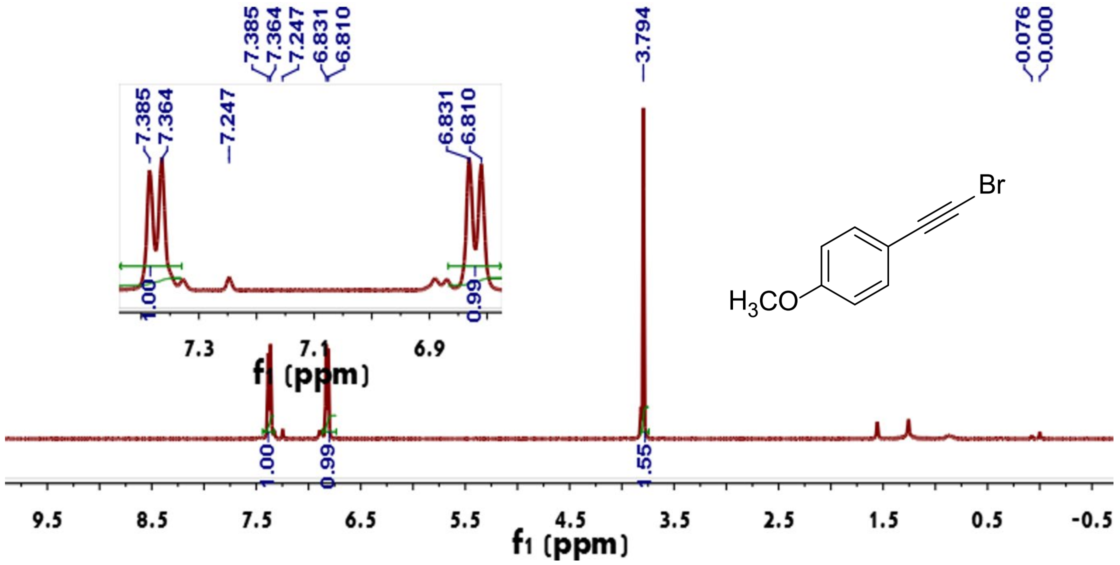

$^{13}C\{^{1}H\}$ NMR (100 MHz, $CDCl_3$) spectrum of **2d**

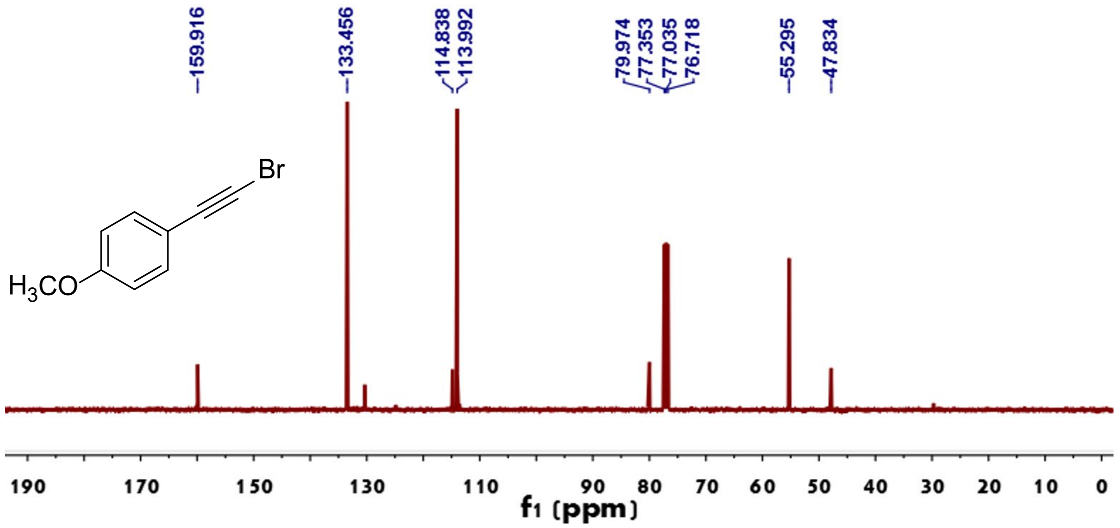

NMR Spectra of (*E*)-1-(1,2-dibromovinyl)-4-methoxybenzene (3d):

^{1}H NMR (400 MHz, $CDCl_3$) spectrum of **3d**

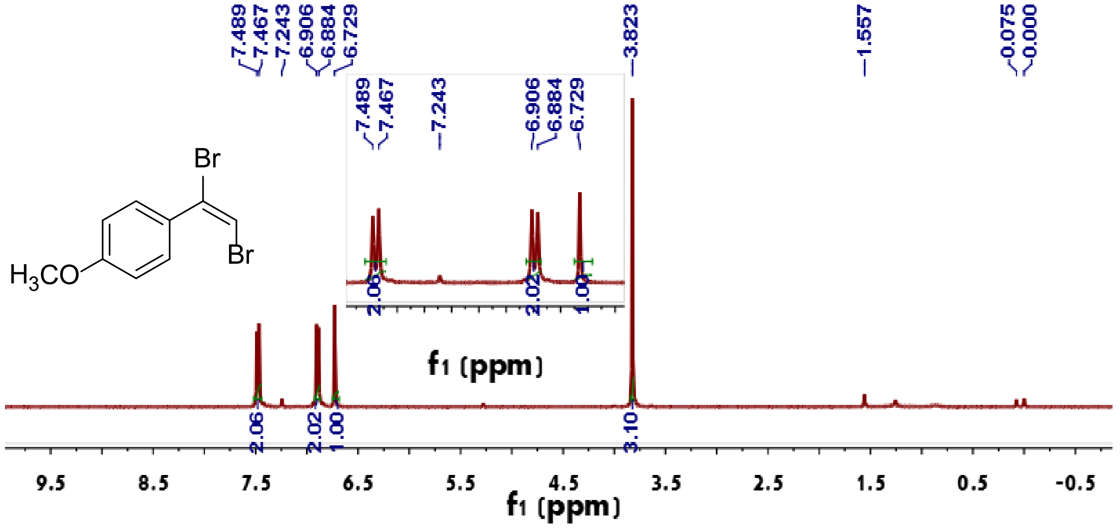

$^{13}C\{^{1}H\}$ NMR (100 MHz, $CDCl_3$) spectrum of **3d**

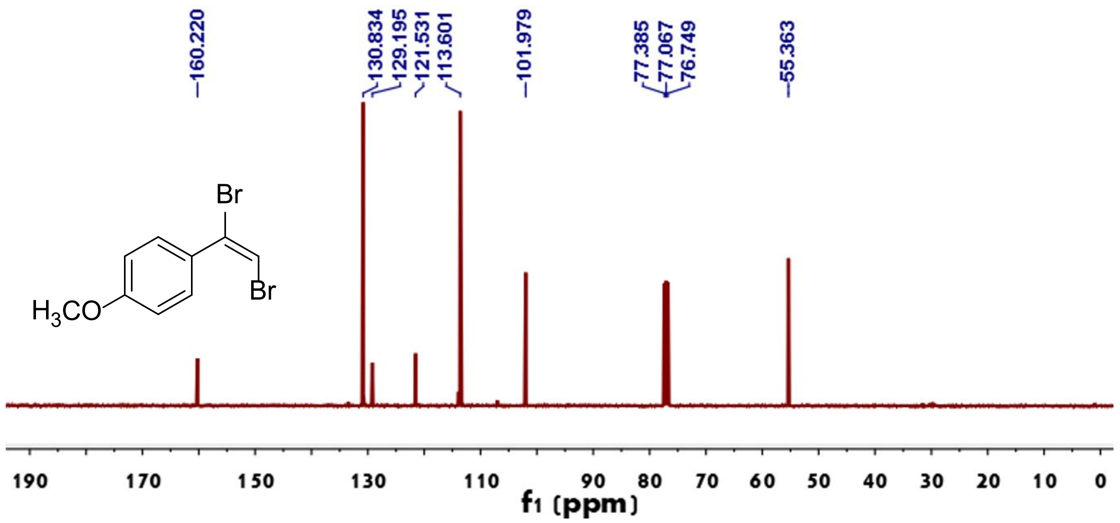

NMR Spectra of (*Z*)-1-(1,2-dibromovinyl)-4-methoxybenzene (4d):

^{1}H NMR (400 MHz, $CDCl_3$) spectrum of **4d**

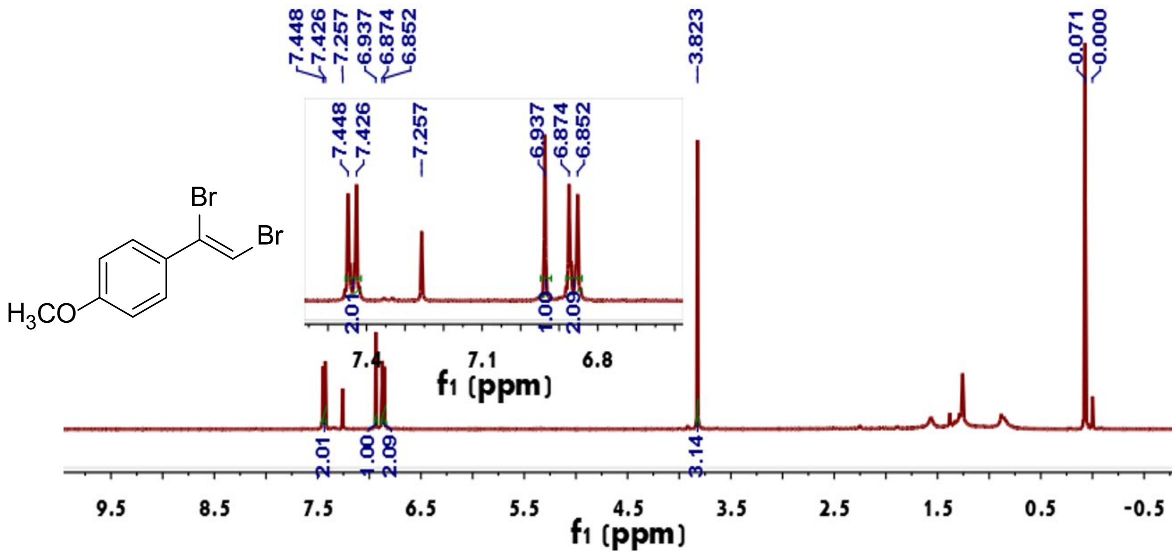

^{13}C{^{1}H} NMR (100 MHz, $CDCl_3$) spectrum of **4d**

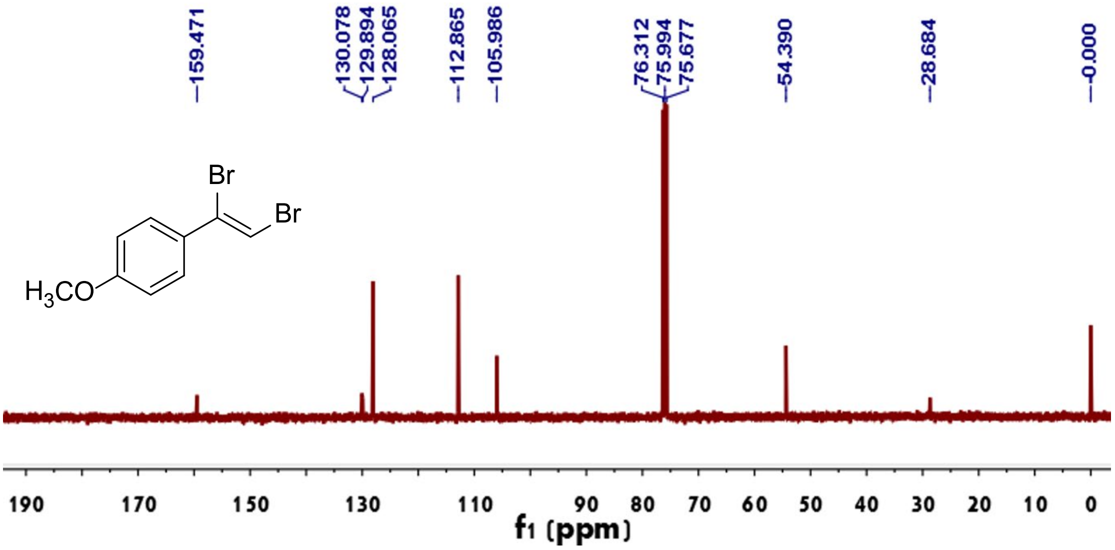

NMR Spectra of 2,2-dibromo-1-(4-methoxyphenyl)ethanone (5d):

^{1}H NMR (500 MHz, $CDCl_3$) spectrum of **5d**

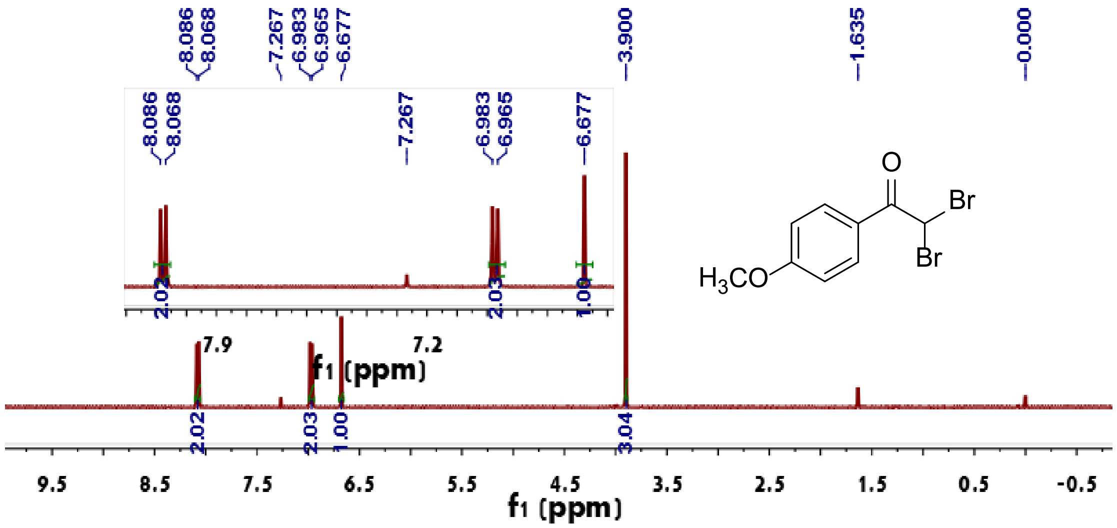

$^{13}C\{^{1}H\}$ NMR (125 MHz, $CDCl_3$) spectrum of **5d**

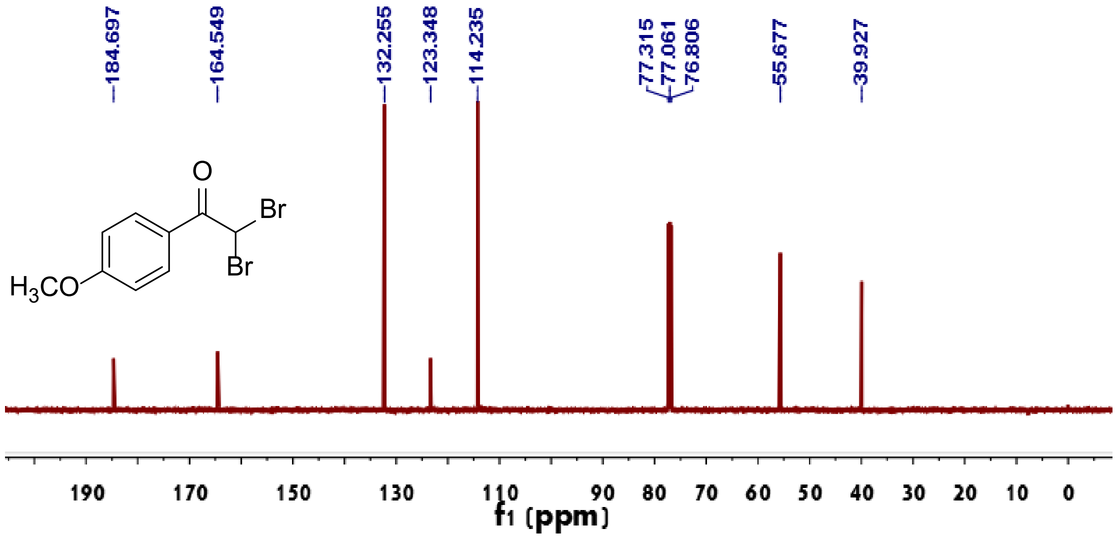

NMR Spectra of methyl 4-(bromoethynyl)benzoate (2e):

^{1}H NMR (400 MHz, $CDCl_3$) spectrum of **2e**

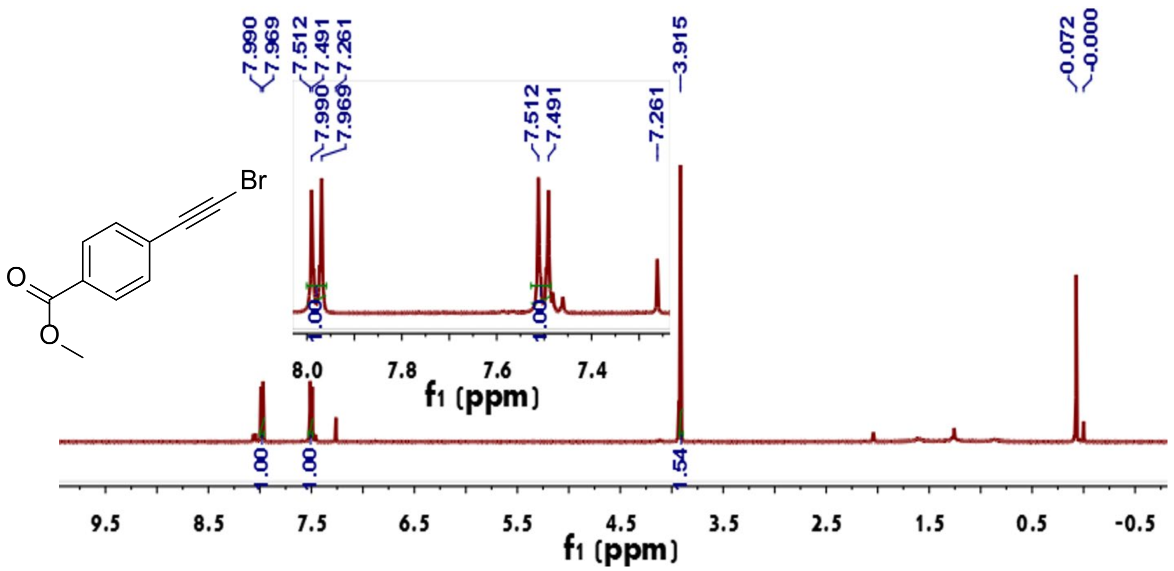

$^{13}C\{^{1}H\}$ NMR (100 MHz, $CDCl_3$) spectrum of **2e**

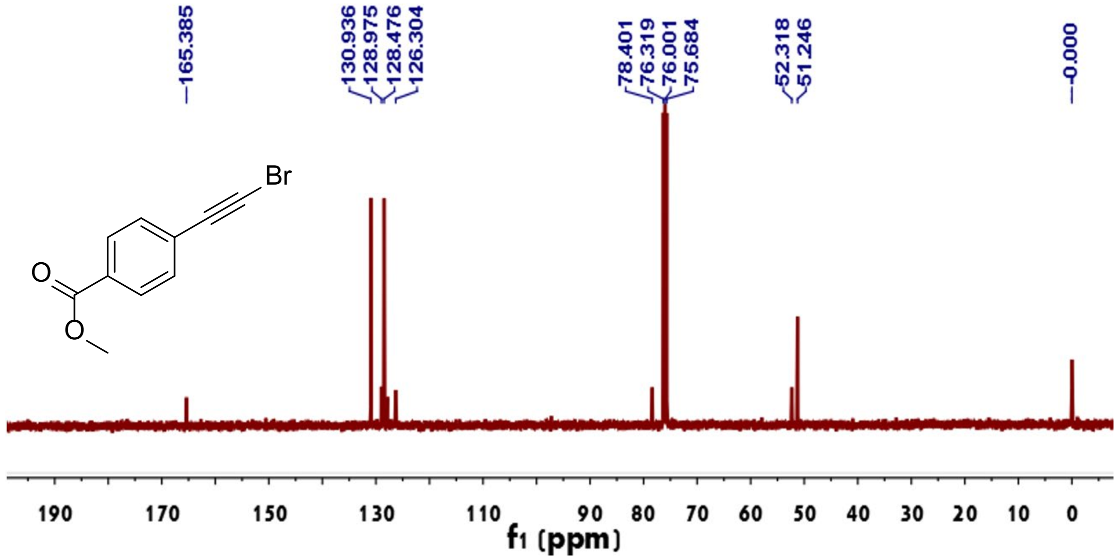

NMR Spectra of (*E*)-methyl 4-(1,2-dibromovinyl)benzoate (3e):

^{1}H NMR (400 MHz, $CDCl_3$) spectrum of **3e**

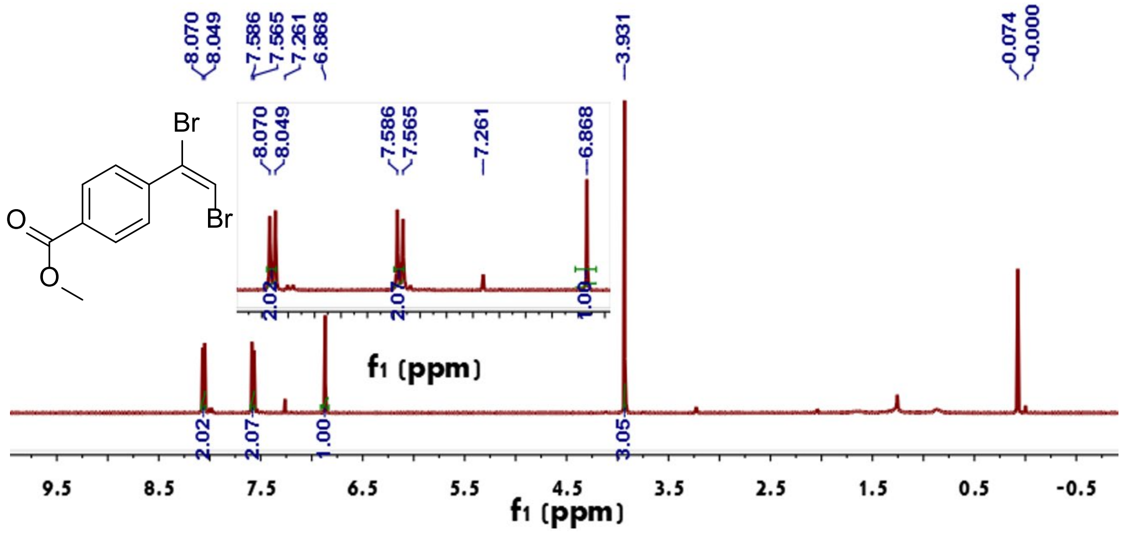

$^{13}C\{^{1}H\}$ NMR (100 MHz, $CDCl_3$) spectrum of **3e**

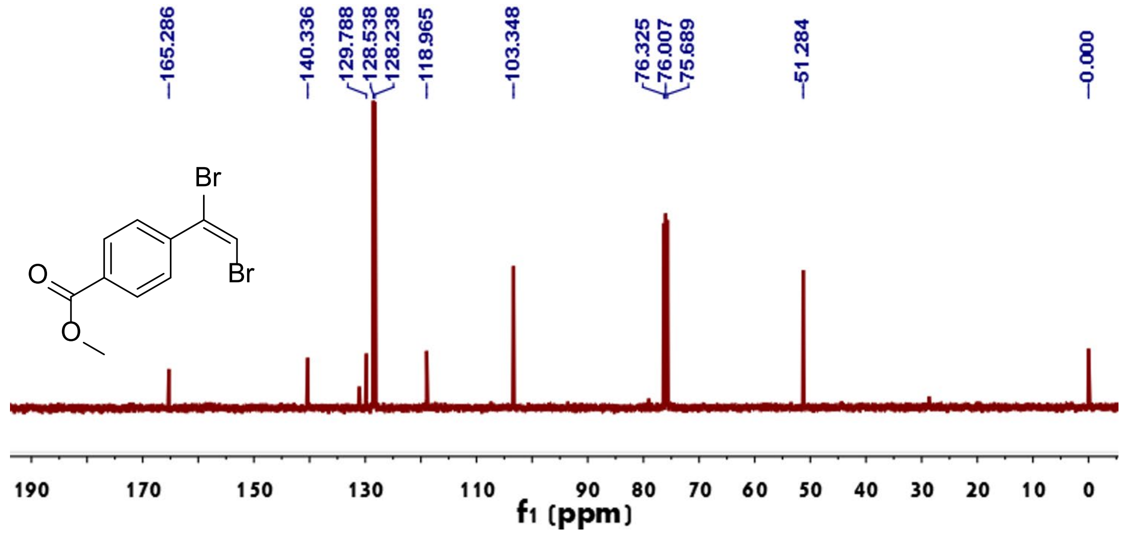

NMR Spectra of (*Z*)-methyl 4-(1,2-dibromovinyl)benzoate (4e):

^{1}H NMR (400 MHz, $CDCl_3$) spectrum of **4e**

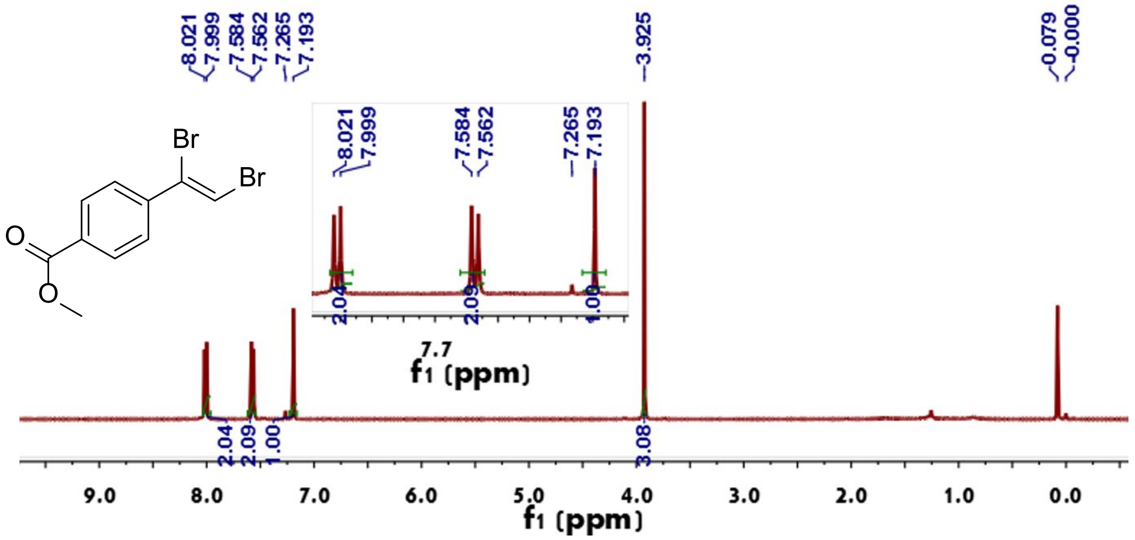

^{13}C{^{1}H} NMR (100 MHz, $CDCl_3$) spectrum of **4e**

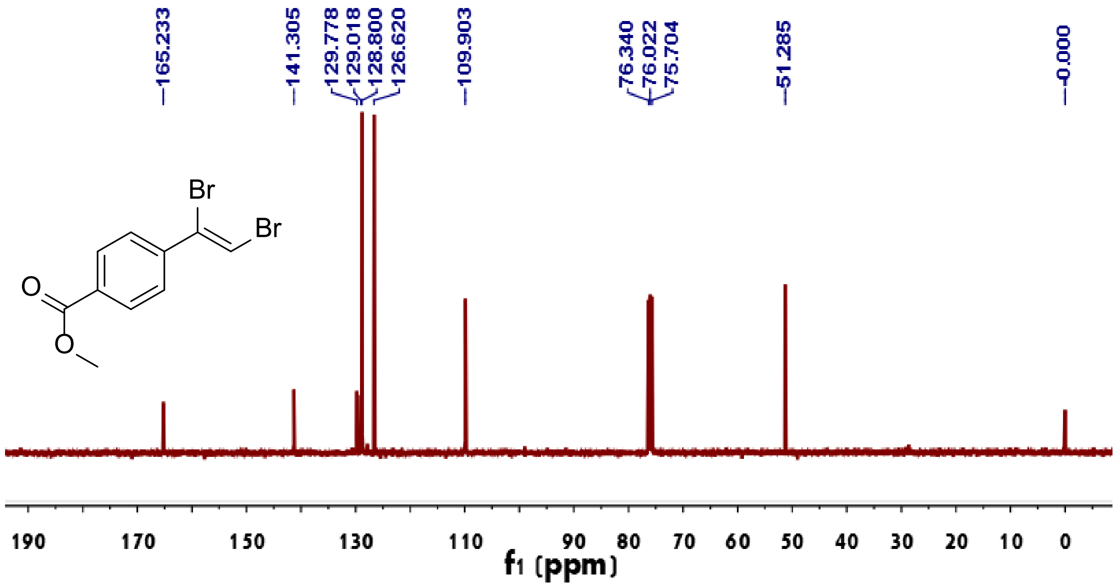

NMR Spectra of methyl 4-(2,2-dibromoacetyl)benzoate (5e):

^{1}H NMR (400 MHz, $CDCl_3$) spectrum of **5e**

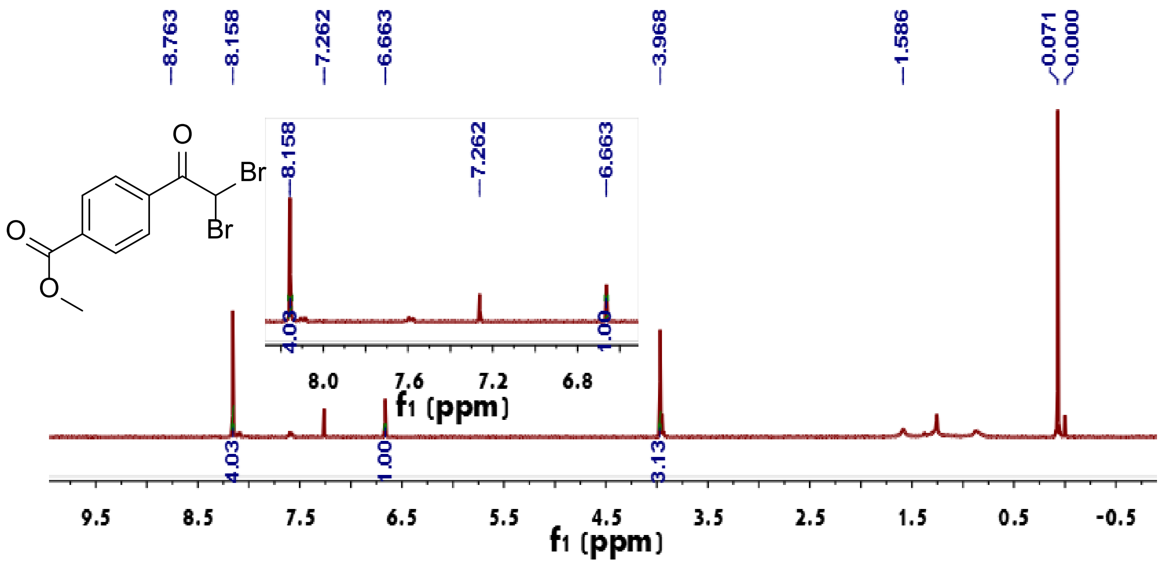

^{13}C{^{1}H} NMR (100 MHz, $CDCl_3$) spectrum of **5e**

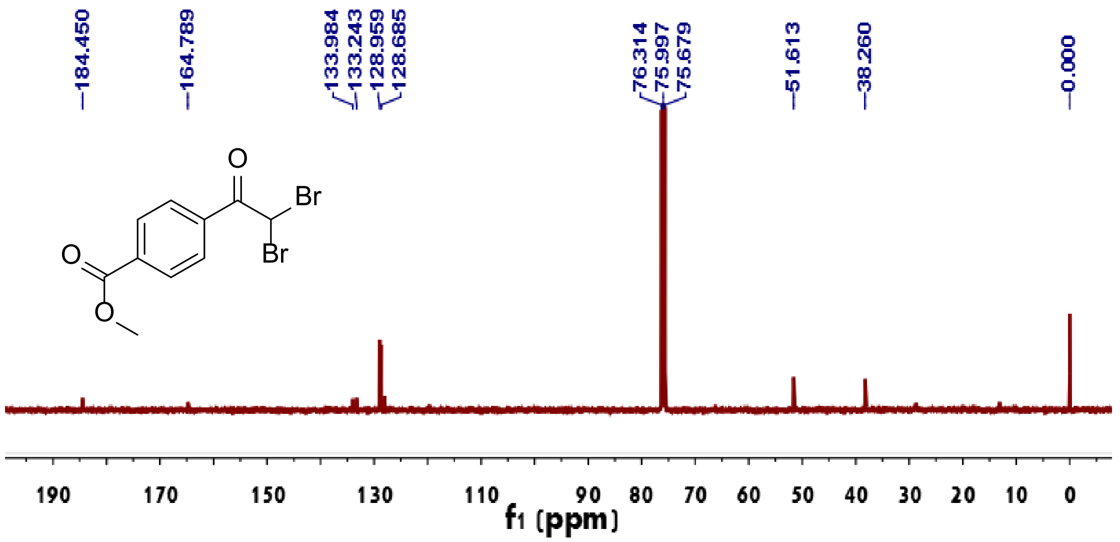

NMR Spectra of methyl 4-(1,1,2,2-tetrabromoethyl)benzoate (6e):

^{1}H NMR (400 MHz, $CDCl_3$) spectrum of **6e**

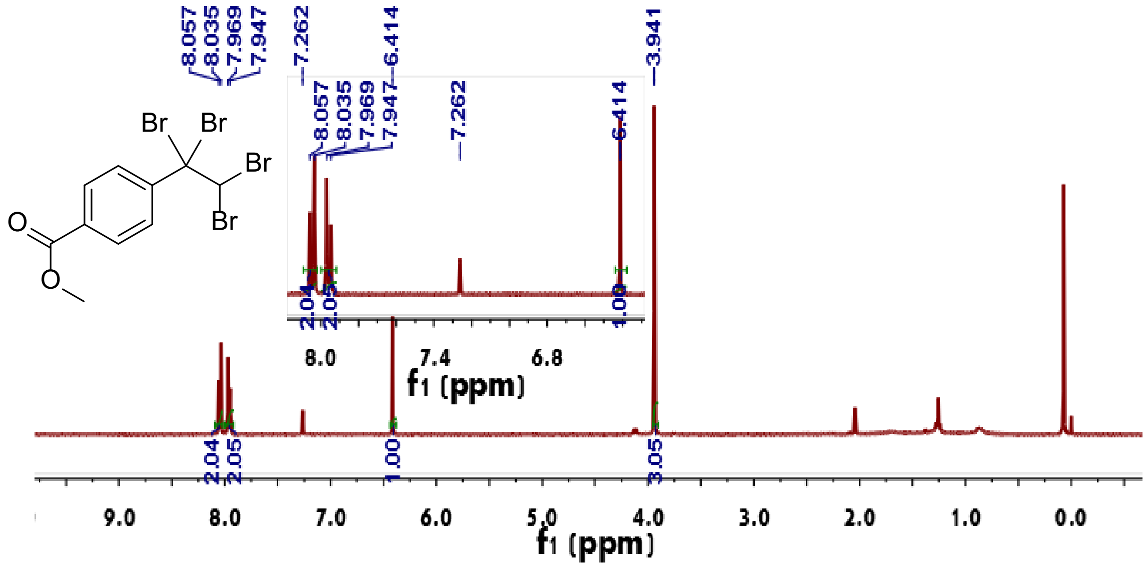

^{13}C{^{1}H} NMR (100 MHz, $CDCl_3$) spectrum of **6e**

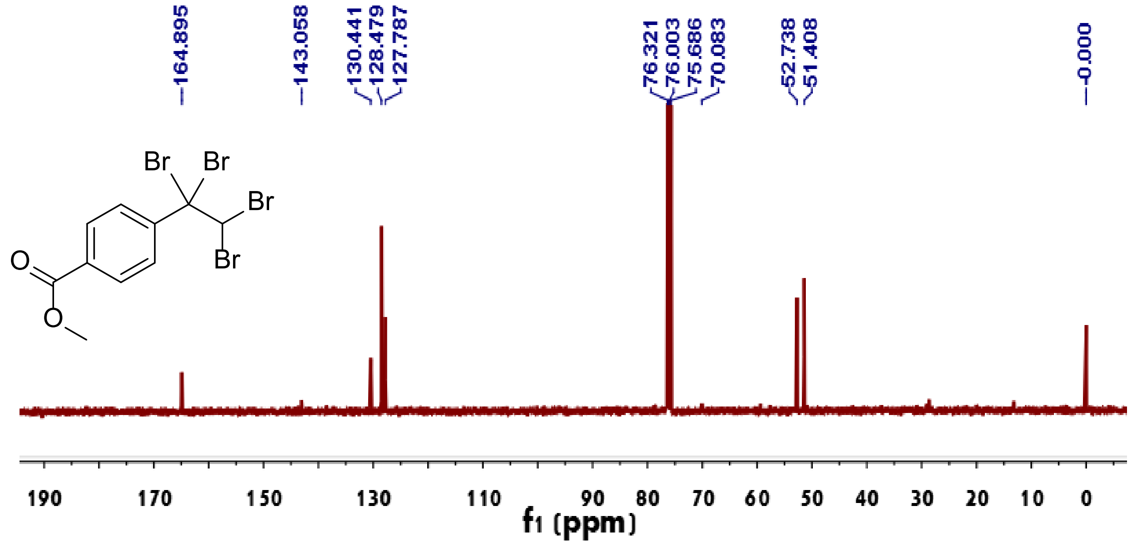

NMR Spectra of 1-(bromoethynyl)-4-fluorobenzene (2f):

^{1}H NMR (400 MHz, $CDCl_3$) spectrum of **2f**

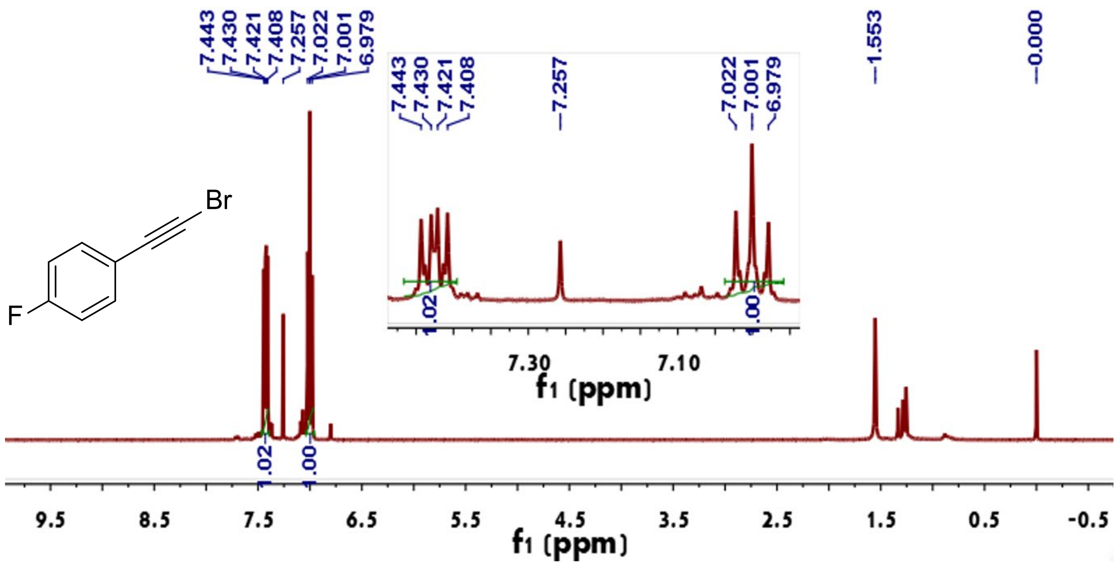

$^{13}C\{^{1}H\}$ NMR (100 MHz, $CDCl_3$) spectrum of **2f**

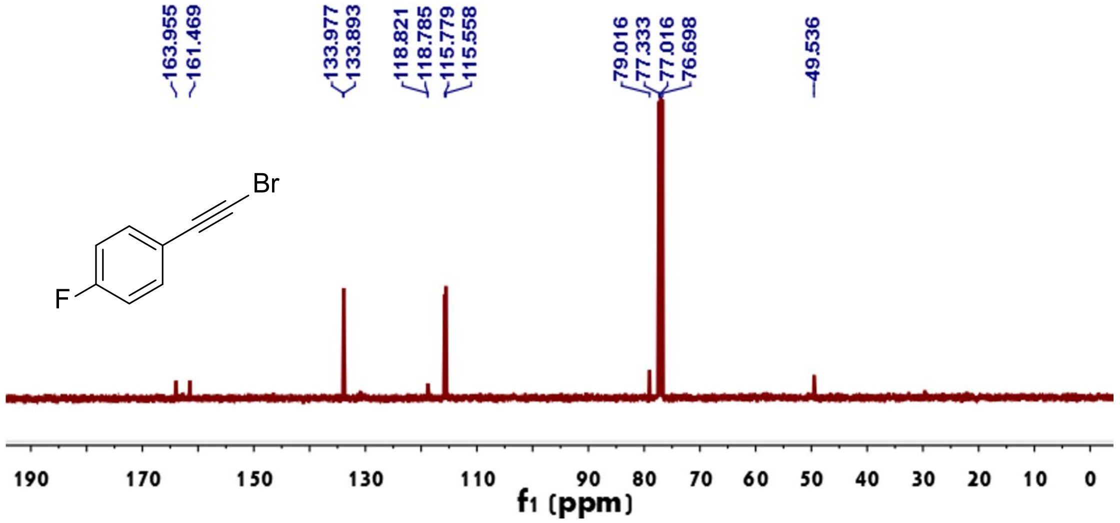

NMR Spectra of (*E*)-1-(1,2-dibromovinyl)-4-fluorobenzene (3f):

^{1}H NMR (400 MHz, $CDCl_3$) spectrum of **3f**

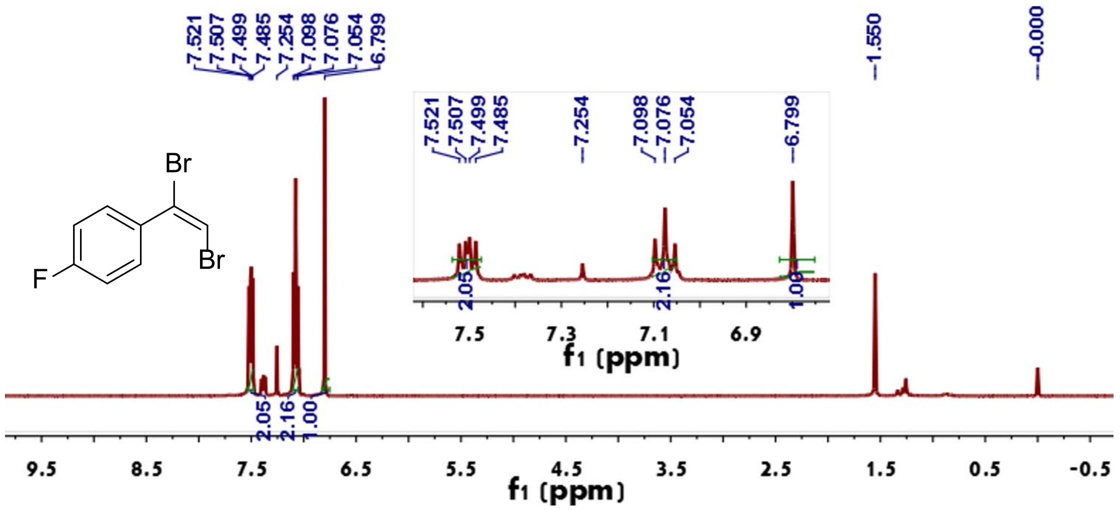

$^{13}C\{^{1}H\}$ NMR (100 MHz, $CDCl_3$) spectrum of **3f**

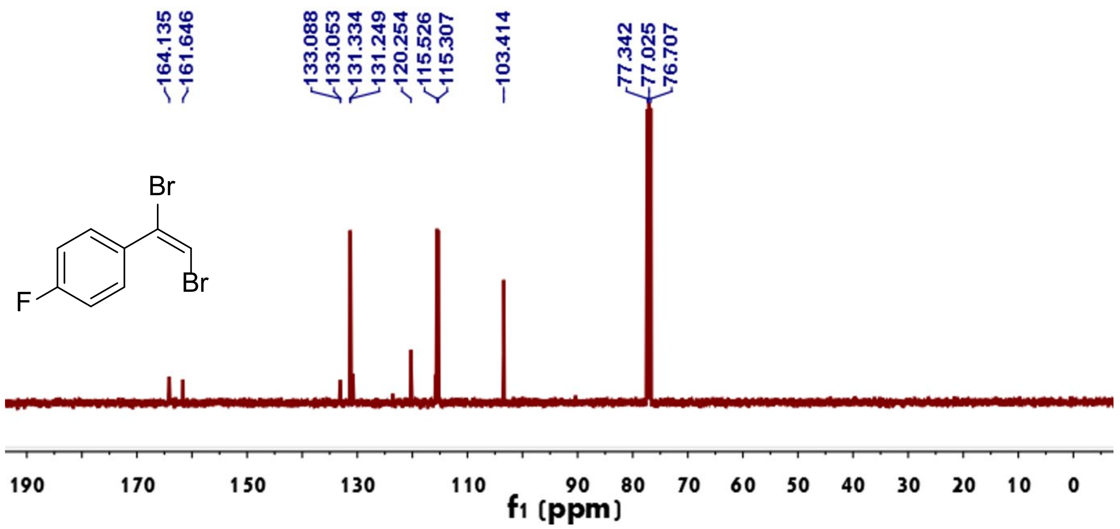

NMR Spectra of (*Z*)-1-(1,2-dibromovinyl)-4-fluorobenzene (4f):

^{1}H NMR (400 MHz, $CDCl_3$) spectrum of **4f**

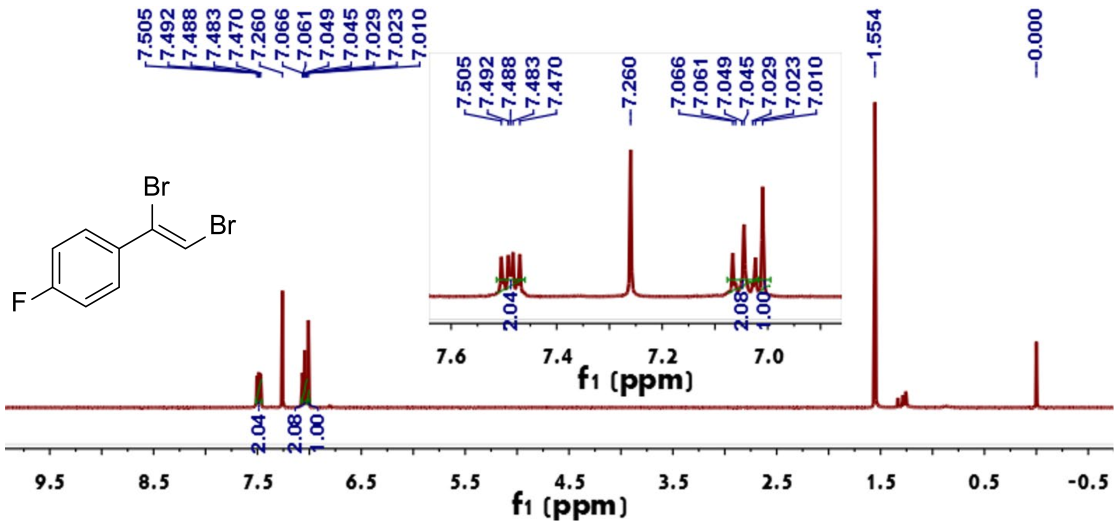

$^{13}C\{^1H\}$ NMR (100 MHz, $CDCl_3$) spectrum of **4f**

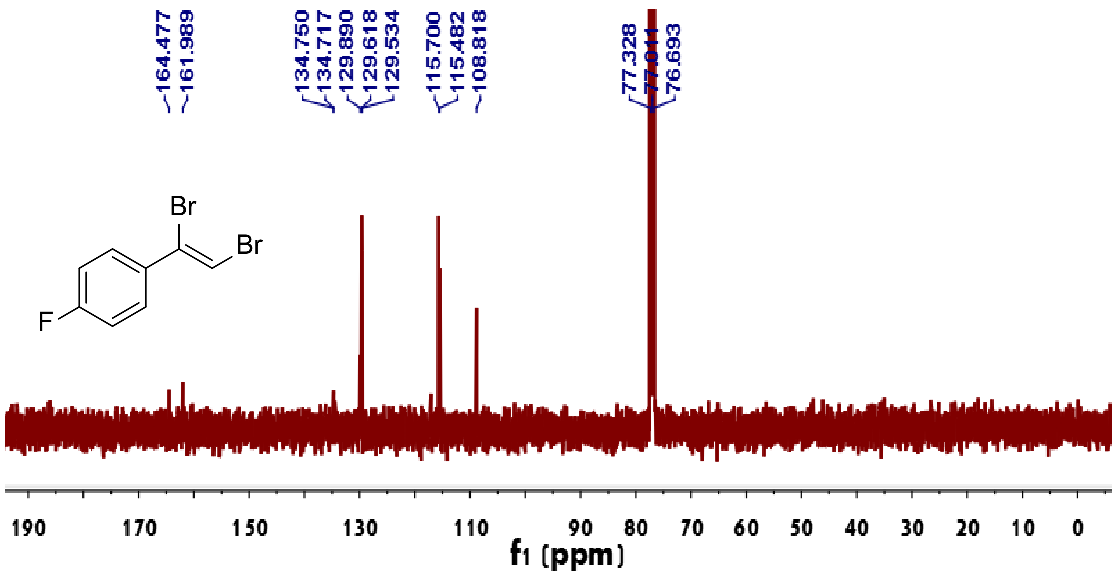

NMR Spectra of 2,2-dibromo-1-(4-fluorophenyl)ethanone (5f):

^{1}H NMR (400 MHz, $CDCl_3$) spectrum of **5f**

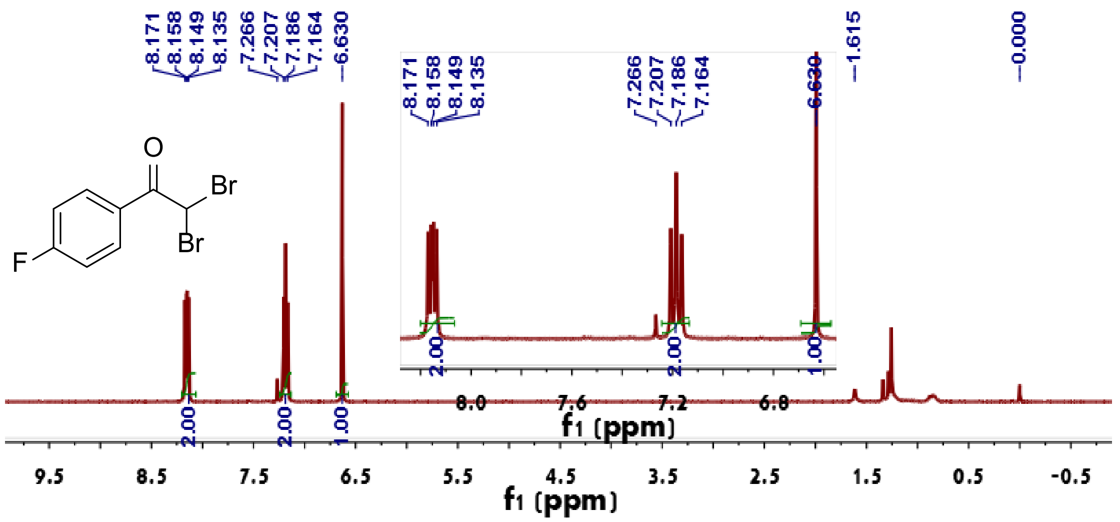

^{13}C{^{1}H} NMR (100 MHz, $CDCl_3$) spectrum of **5f**

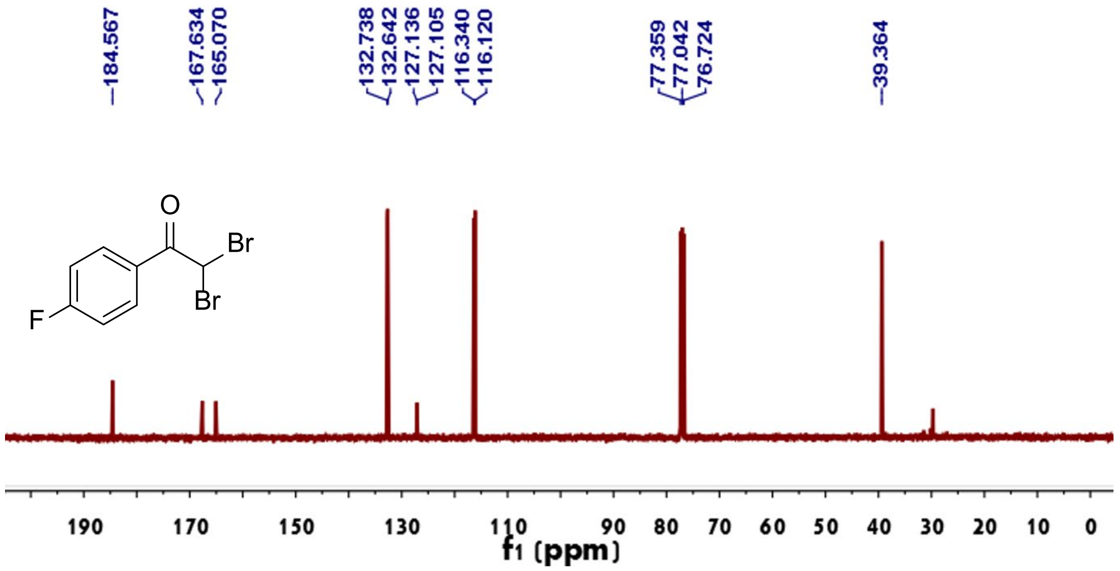

NMR Spectra of 1-fluoro-4-(1,1,2,2-tetrabromoethyl)benzene (6f):

^{1}H NMR (400 MHz, $CDCl_3$) spectrum of **6f**

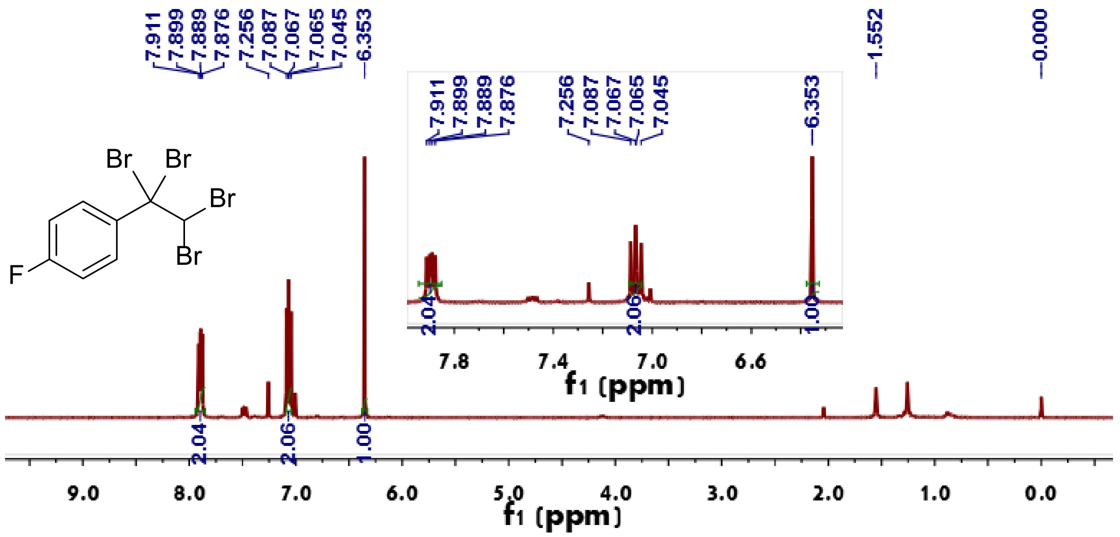

^{13}C{^{1}H} NMR (100 MHz, $CDCl_3$) spectrum of **6f**

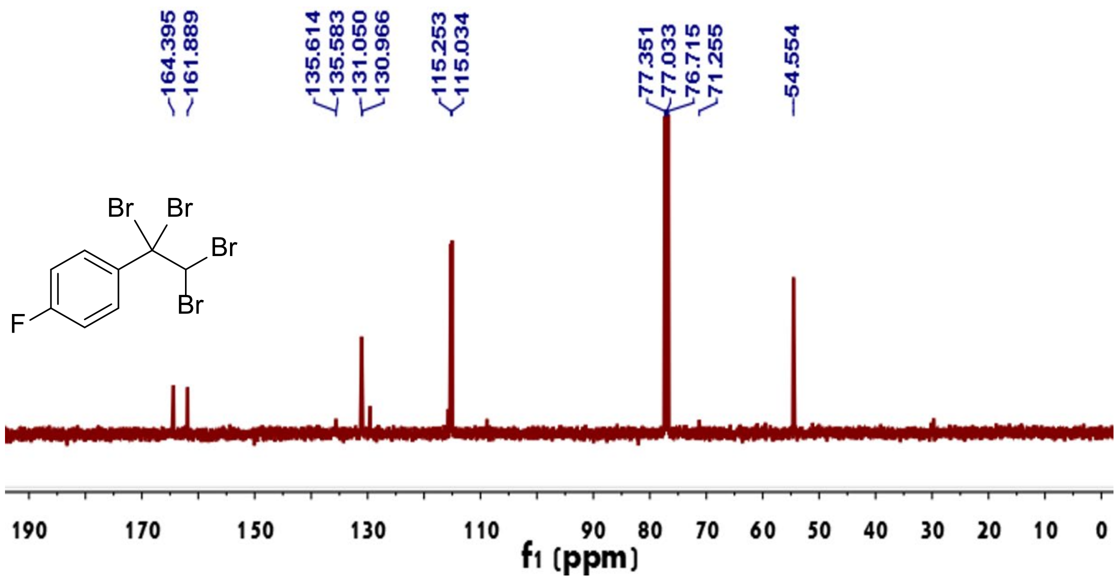

NMR Spectra of 1-(bromoethynyl)-4-chlorobenzene (2g):

^{1}H NMR (400 MHz, $CDCl_3$) spectrum of **2g**

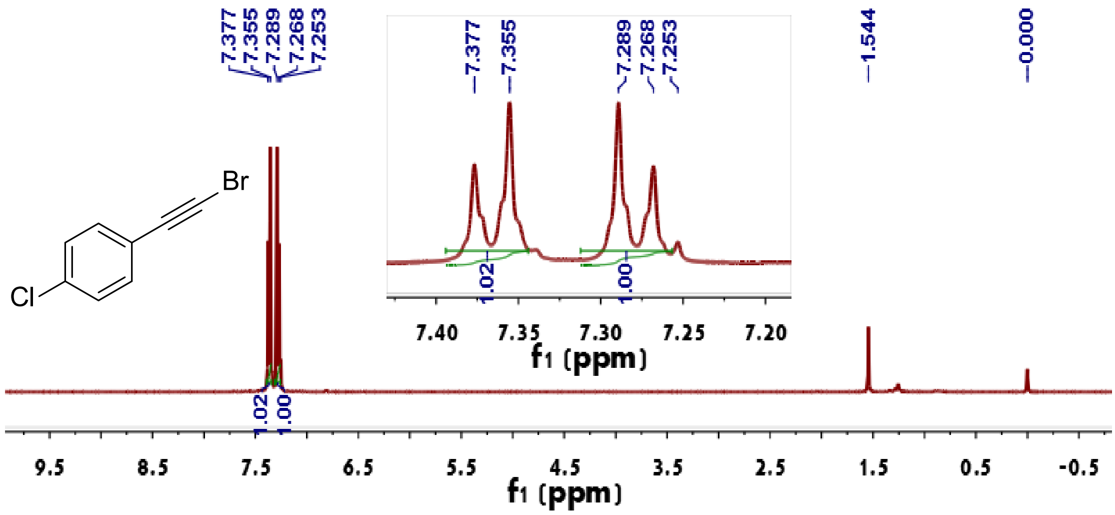

^{13}C{^{1}H} NMR (100 MHz, $CDCl_3$) spectrum of **2g**

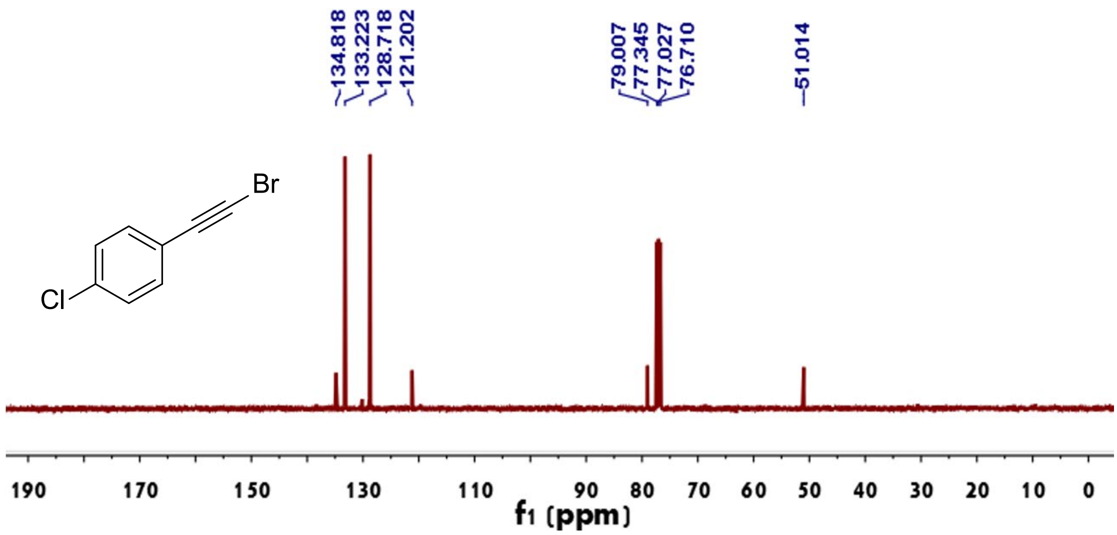

NMR Spectra of (*E*)-1-chloro-4-(1,2-dibromovinyl)benzene (3g):

^{1}H NMR (400 MHz, $CDCl_3$) spectrum of **3g**

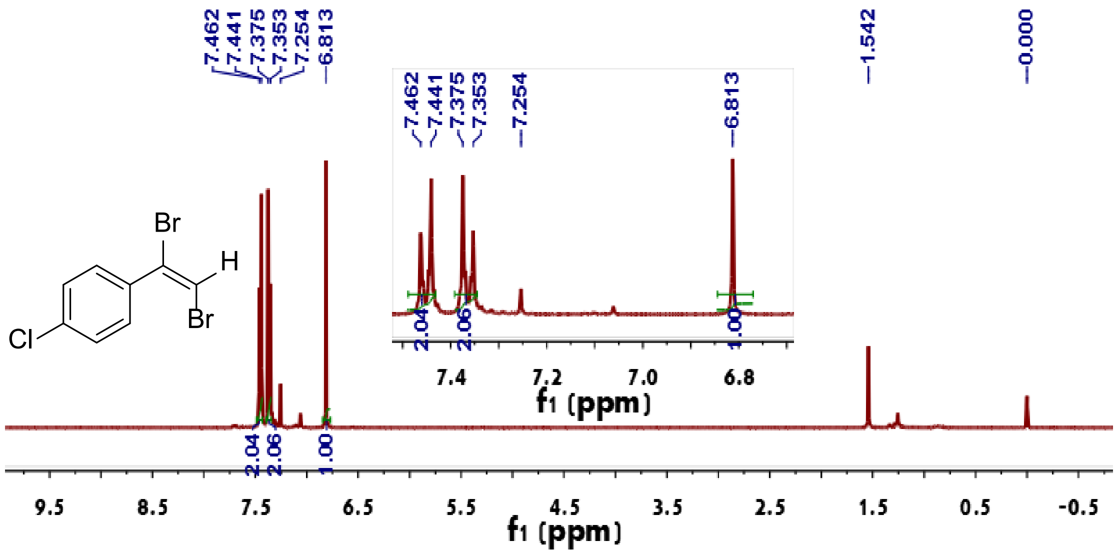

$^{13}C\{^{1}H\}$ NMR (100 MHz, $CDCl_3$) spectrum of **3g**

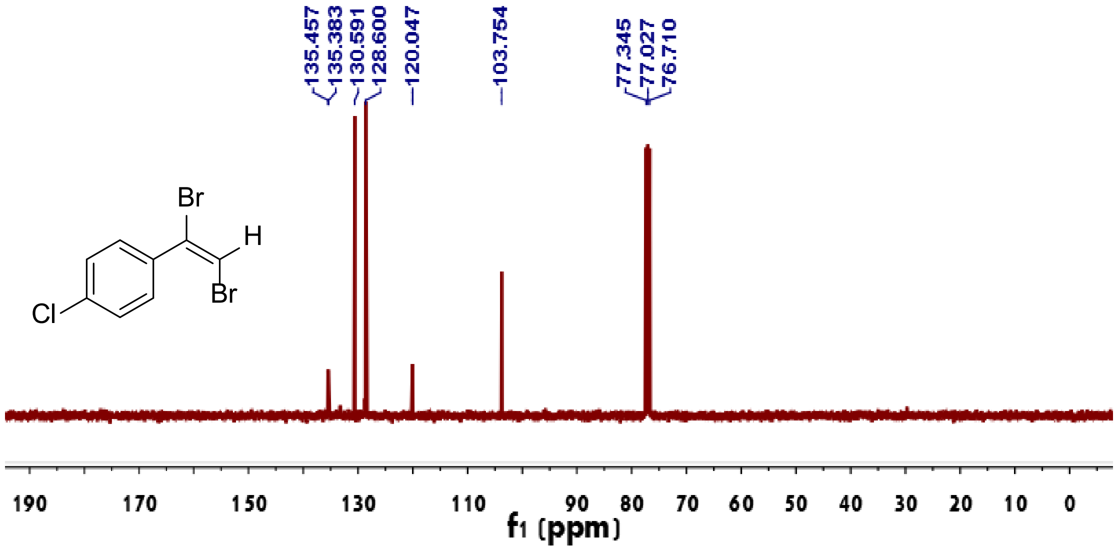

NMR Spectra of (*Z*)-1-chloro-4-(1,2-dibromovinyl)benzene (4g):

^{1}H NMR (400 MHz, $CDCl_3$) spectrum of **4g**

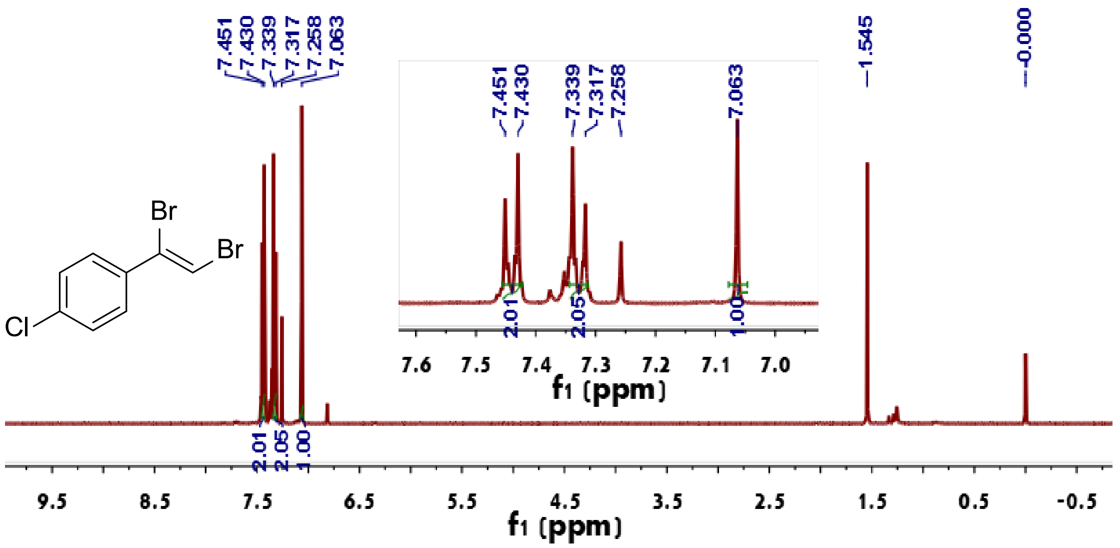

^{13}C{^{1}H} NMR (100 MHz, $CDCl_3$) spectrum of **4g**

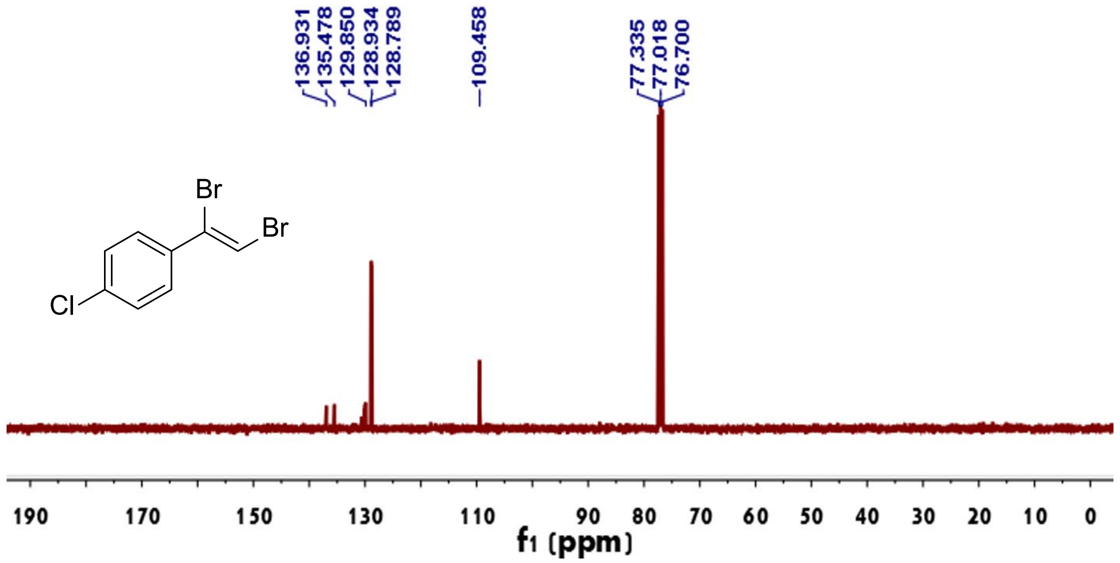

NMR Spectra of 2,2-dibromo-1-(4-chlorophenyl)ethanone (5g):

^{1}H NMR (400 MHz, $CDCl_3$) spectrum of **5g**

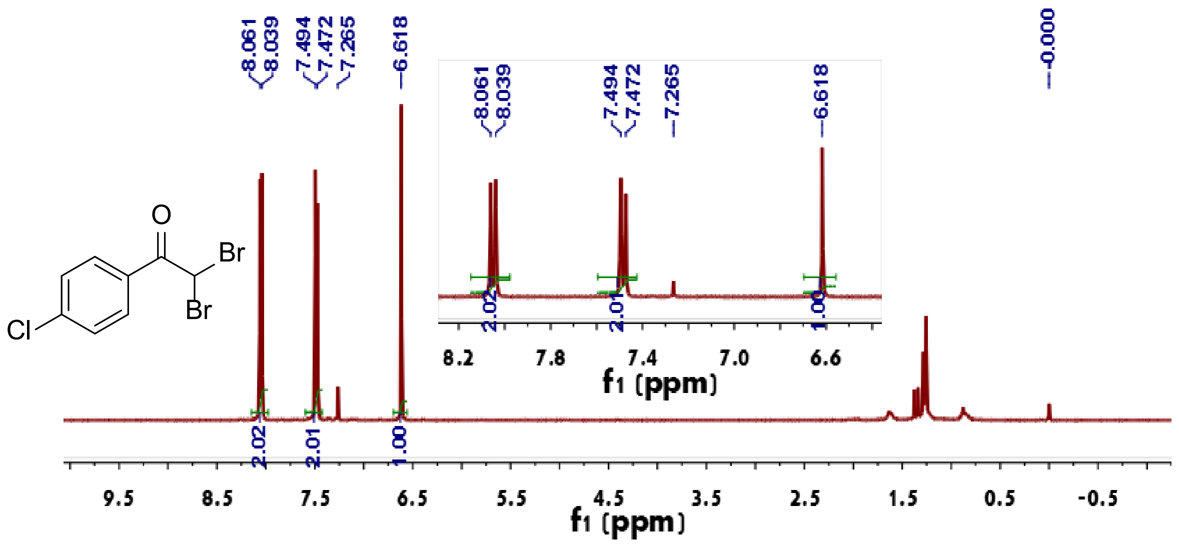

^{13}C{^{1}H} NMR (100 MHz, $CDCl_3$) spectrum of **5g**

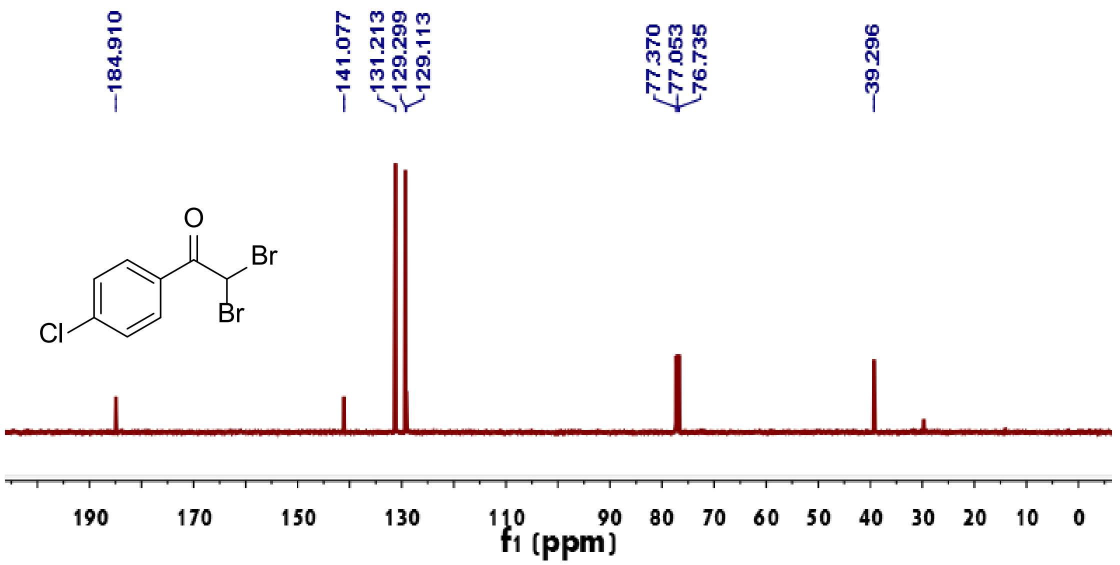

NMR Spectra of 1-chloro-4-(1,1,2,2-tetrabromoethyl)benzene (6g):

^{1}H NMR (400 MHz, $CDCl_3$) spectrum of **6g**

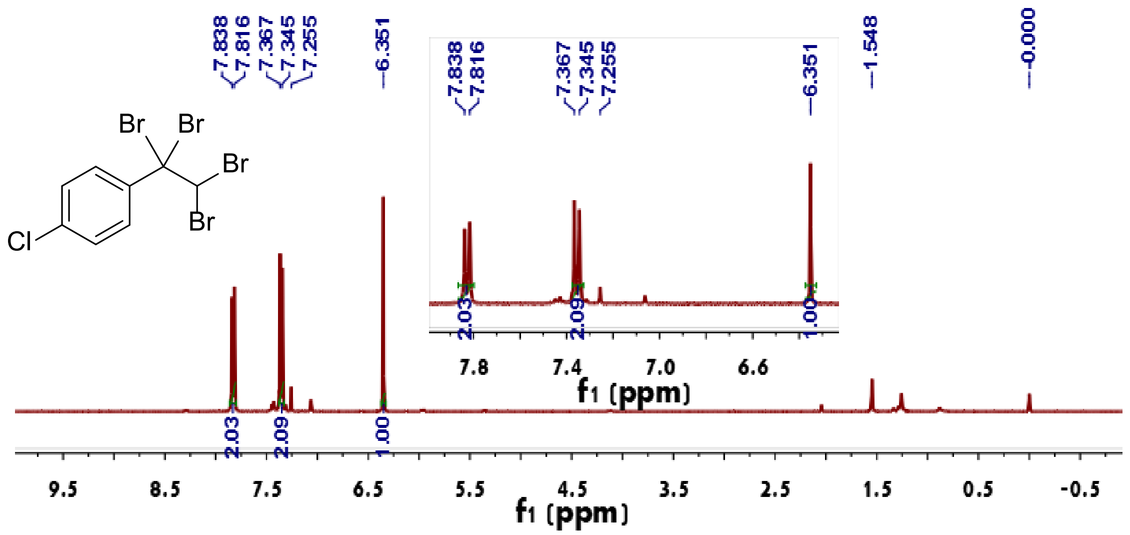

^{13}C{^{1}H} NMR (100 MHz, $CDCl_3$) spectrum of **6g**

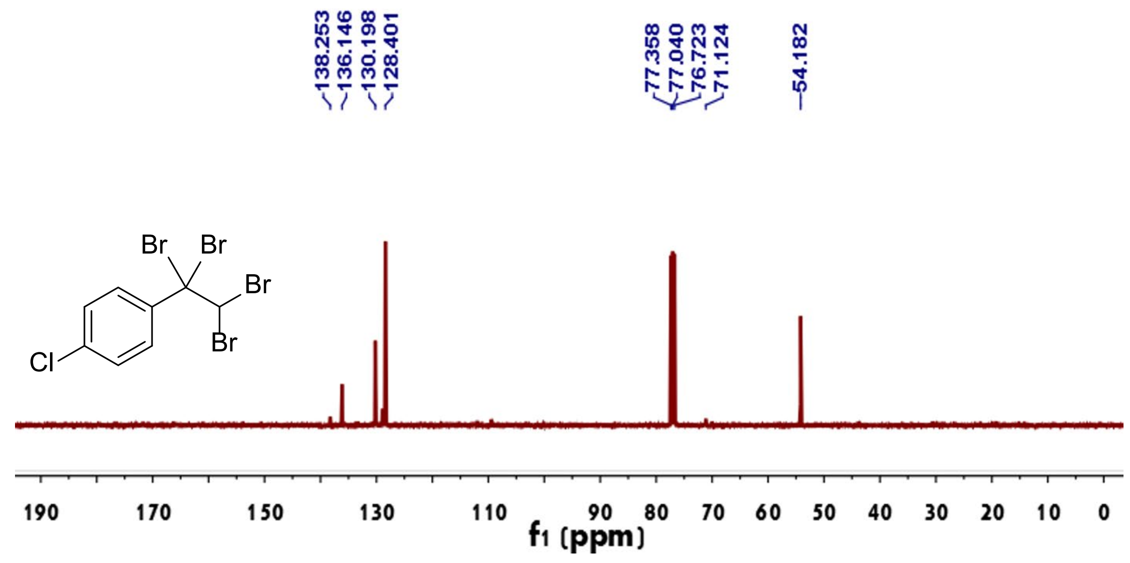

NMR Spectra of 1-bromo-4-(bromoethynyl)benzene (2h):

^{1}H NMR (400 MHz, $CDCl_3$) spectrum of **2h**

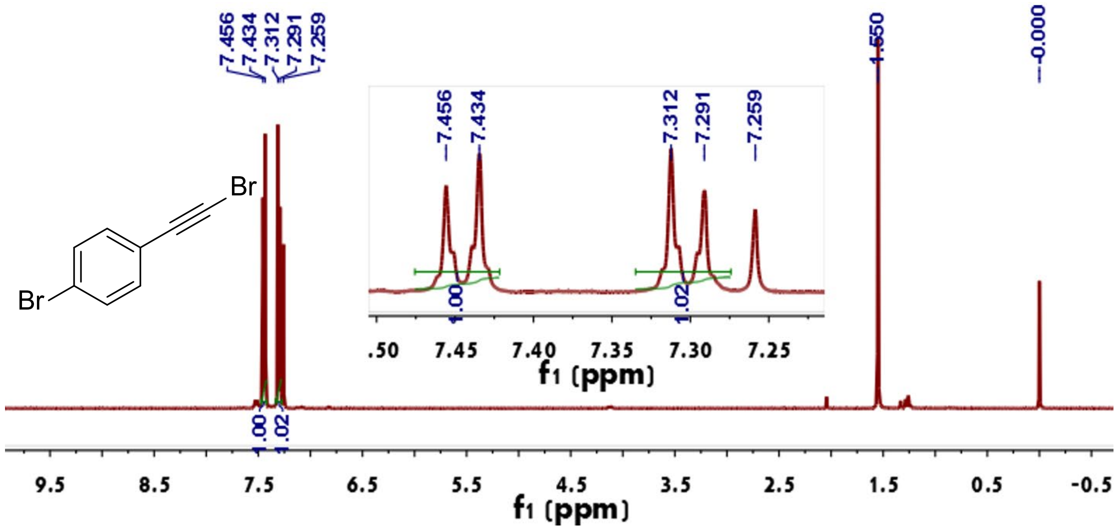

^{13}C{^{1}H} NMR (100 MHz, $CDCl_3$) spectrum of **2h**

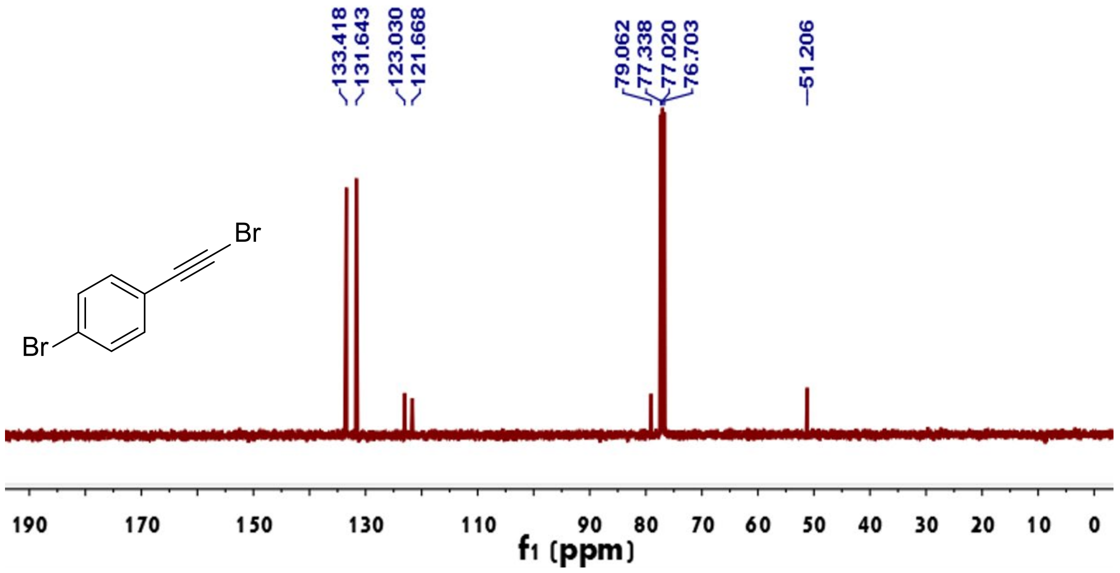

NMR Spectra of (*E*)-1-bromo-4-(1,2-dibromovinyl)benzene (3h):

^{1}H NMR (400 MHz, $CDCl_3$) spectrum of **3h**

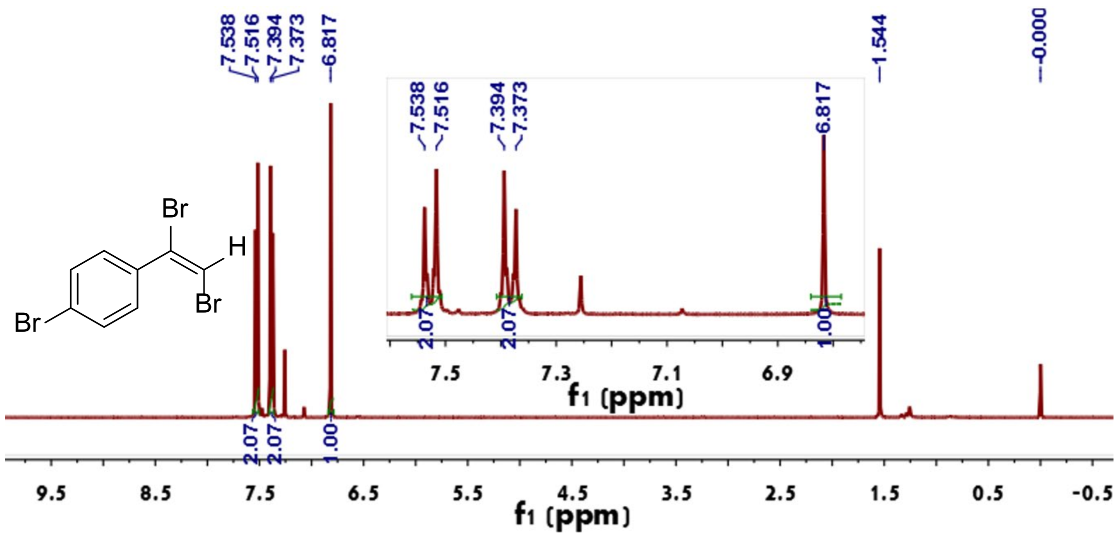

^{13}C{^{1}H} NMR (100 MHz, $CDCl_3$) spectrum of **3h**

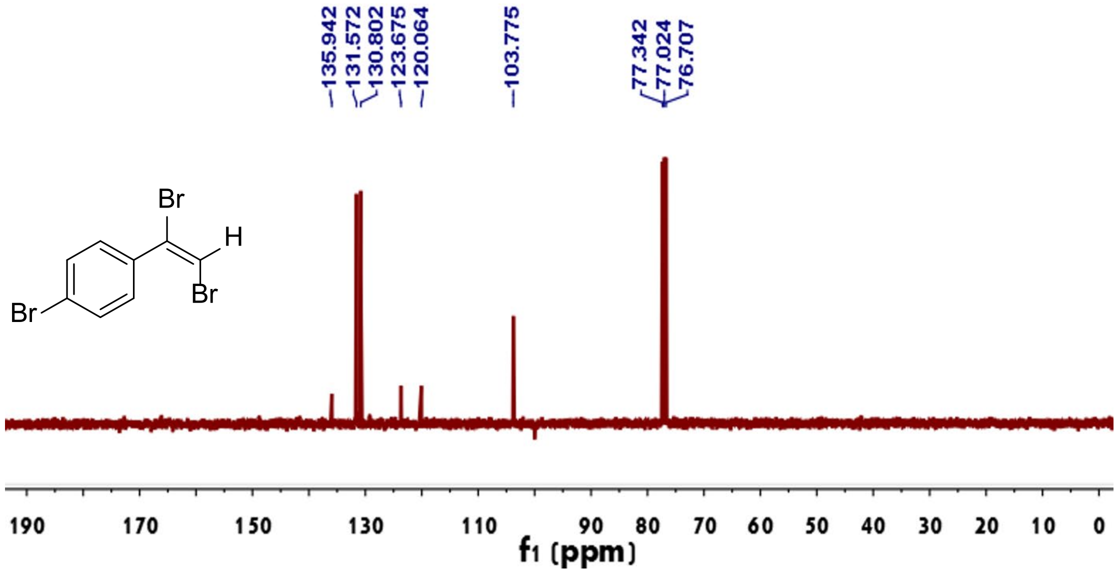

NMR Spectra of (*Z*)-1-bromo-4-(1,2-dibromovinyl)benzene (4h):

^{1}H NMR (400 MHz, $CDCl_3$) spectrum of **4h**

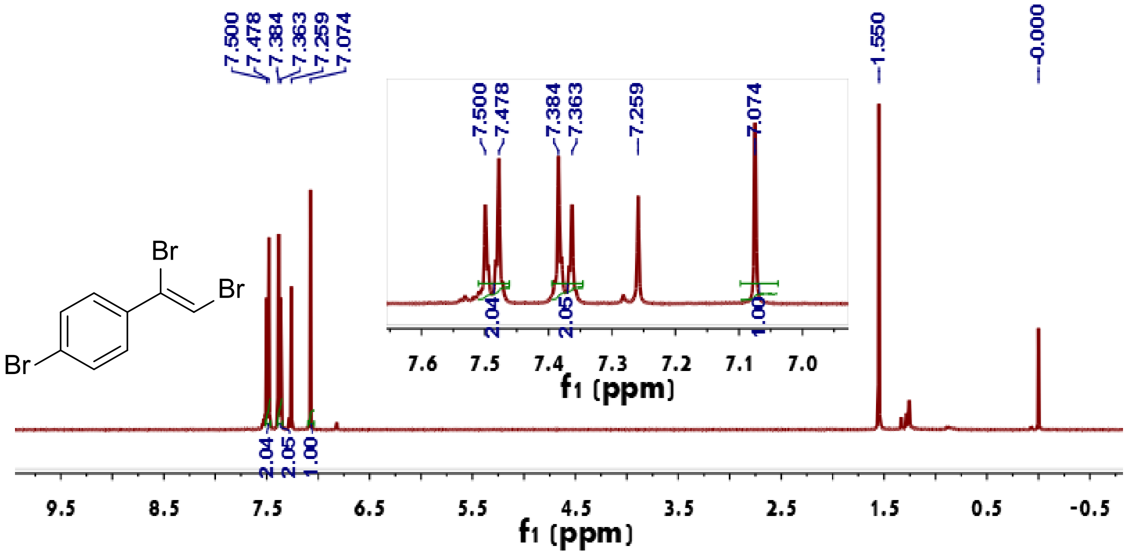

^{13}C{^{1}H} NMR (100 MHz, $CDCl_3$) spectrum of **4h**

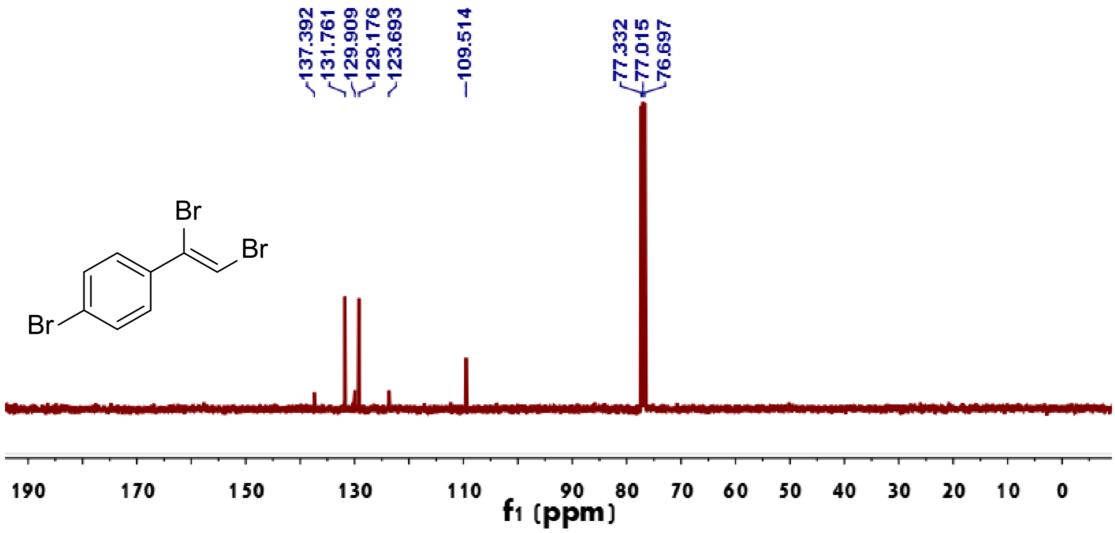

NMR Spectra of 2,2-dibromo-1-(4-bromophenyl)ethanone (5h):

^{1}H NMR (400 MHz, $CDCl_3$) spectrum of **5h**

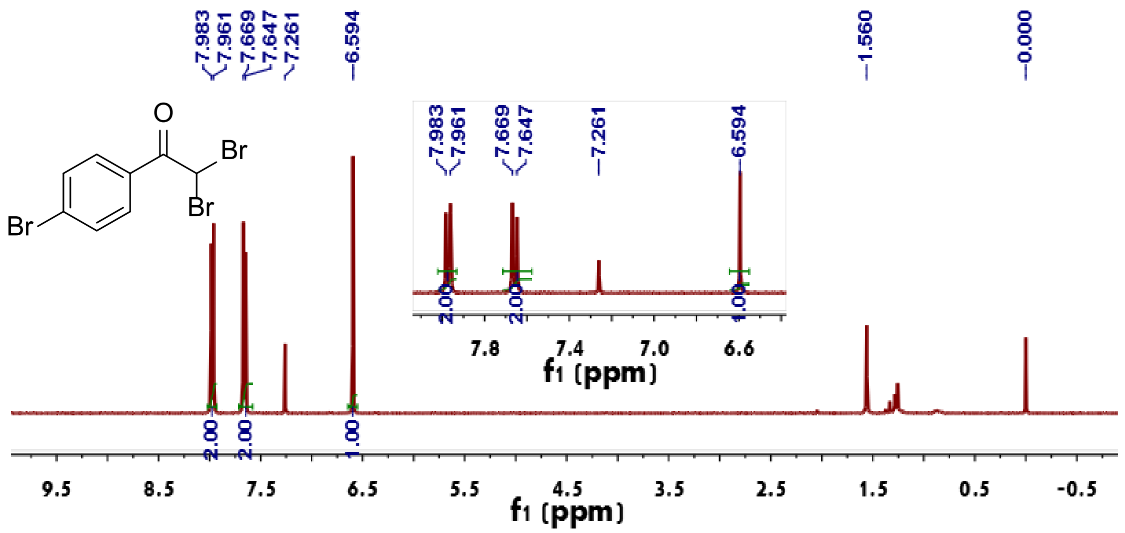

^{13}C{^{1}H} NMR (100 MHz, $CDCl_3$) spectrum of **5h**

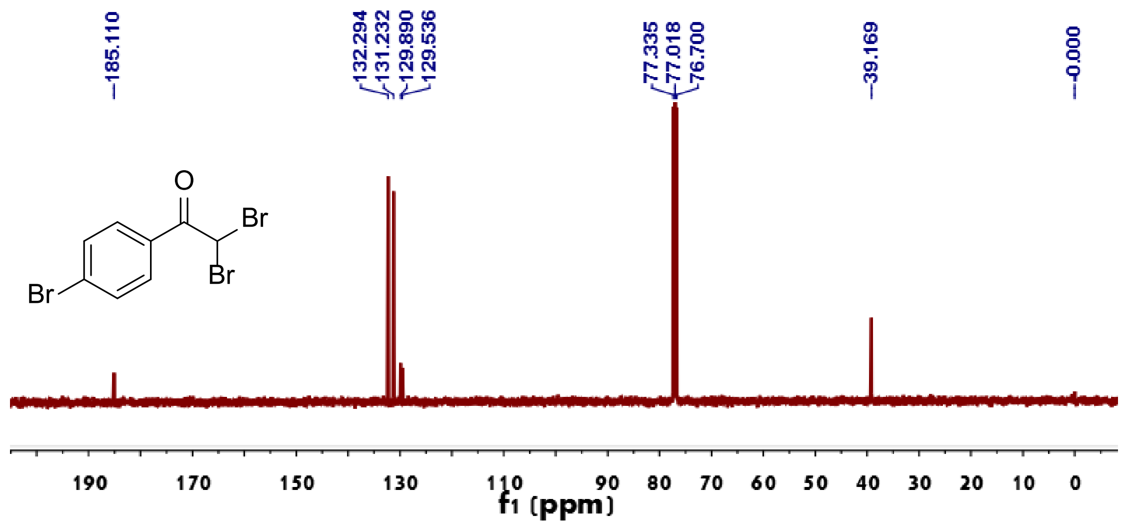

NMR Spectra of 1-bromo-4-(1,1,2,2-tetrabromoethyl)benzene (6h):

^{1}H NMR (400 MHz, $CDCl_3$) spectrum of **6h**

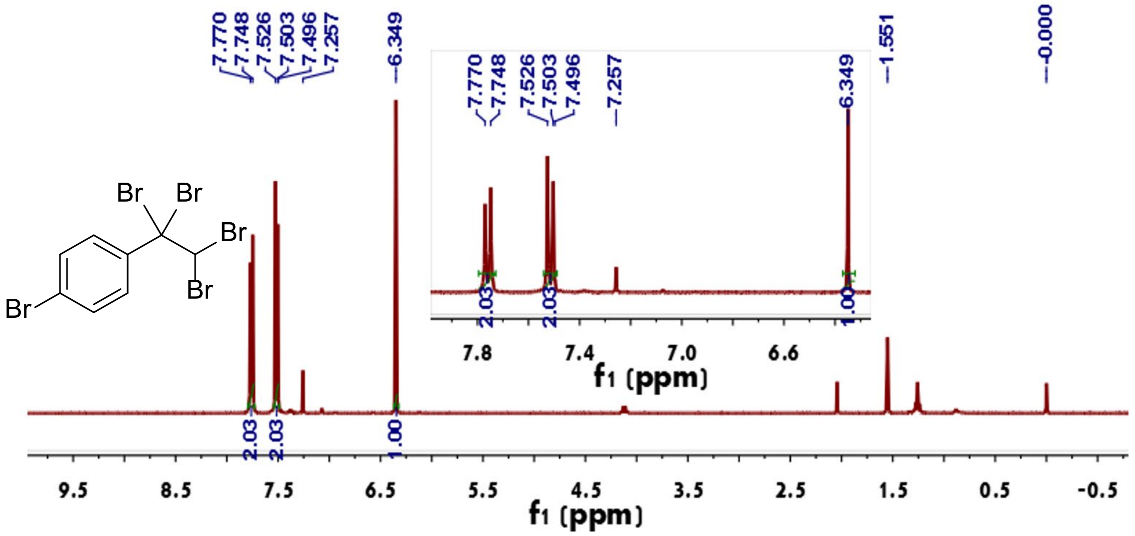

^{13}C{^{1}H} NMR (100 MHz, $CDCl_3$) spectrum of **6h**

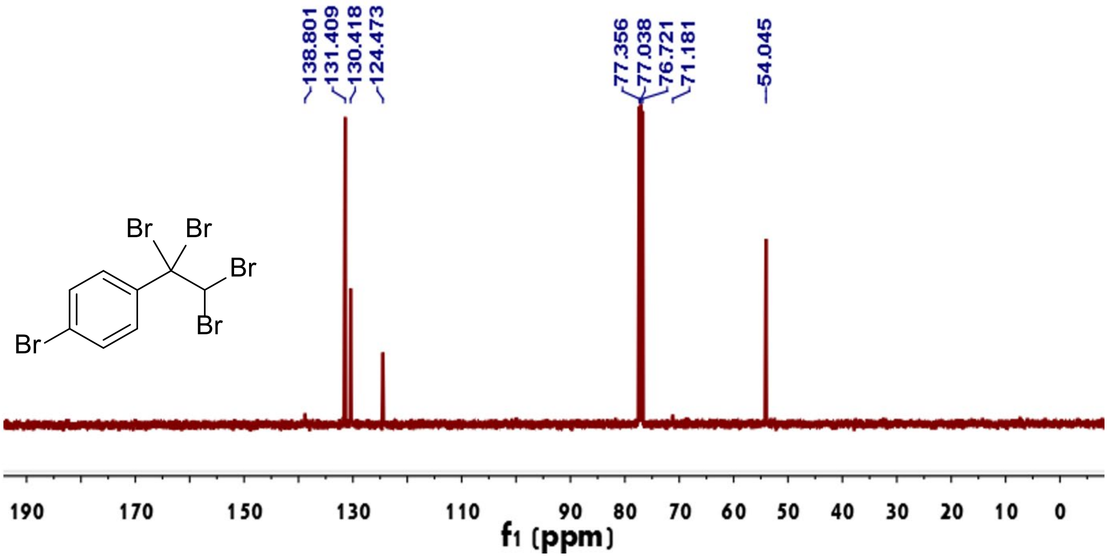

NMR Spectra of 1-(bromoethynyl)-4-(trifluoromethyl)benzene (2i):

^{1}H NMR (400 MHz, $CDCl_3$) spectrum of **2i**

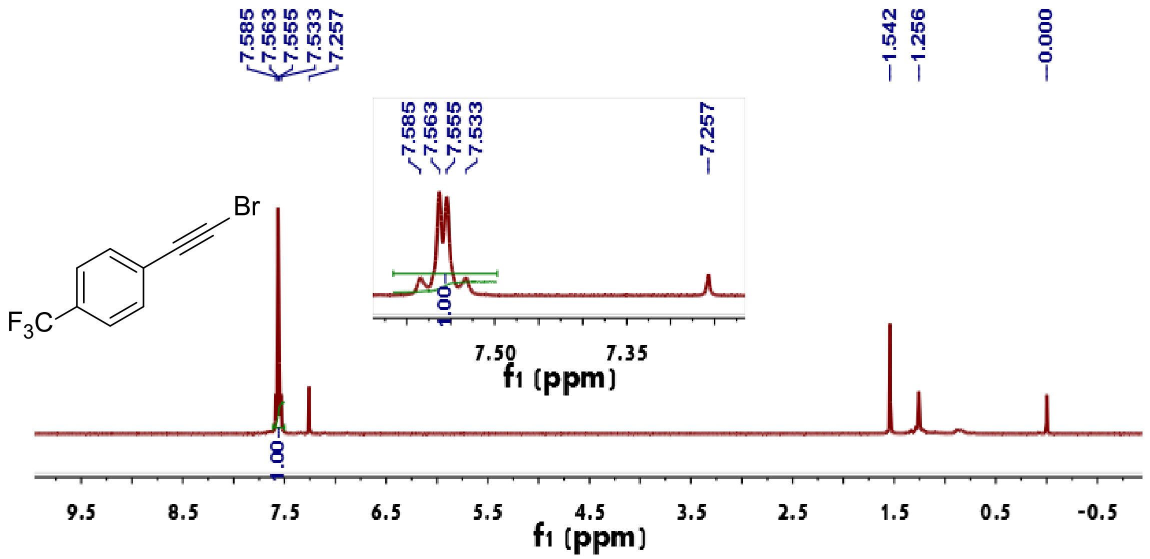

^{13}C{^{1}H} NMR (100 MHz, $CDCl_3$) spectrum of **2i**

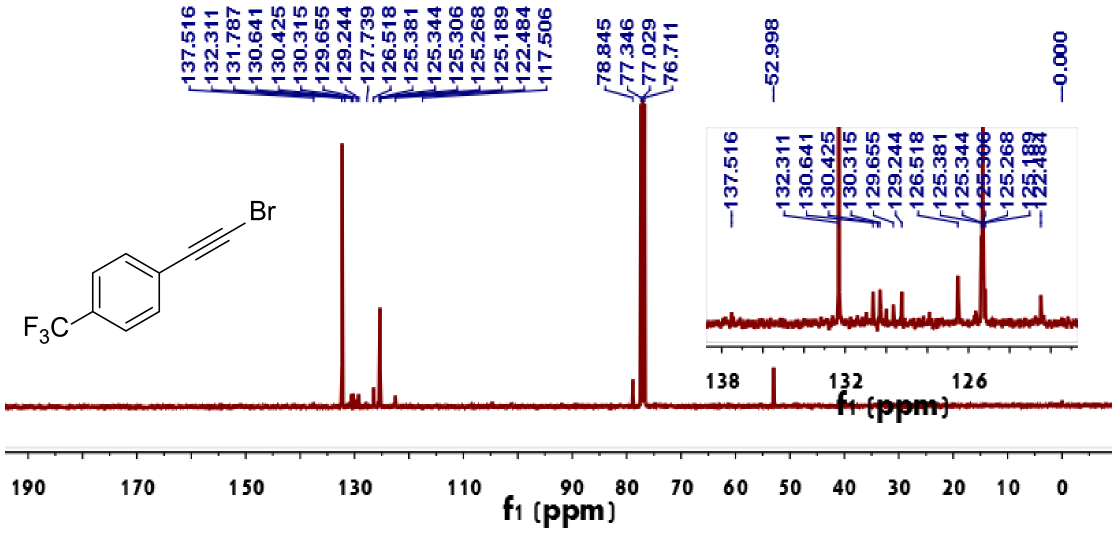

NMR Spectra of (*E*)-1-(1,2-dibromovinyl)-4-(trifluoromethyl)benzene (3i):

^{1}H NMR (400 MHz, CDCl$_3$) spectrum of **3i**

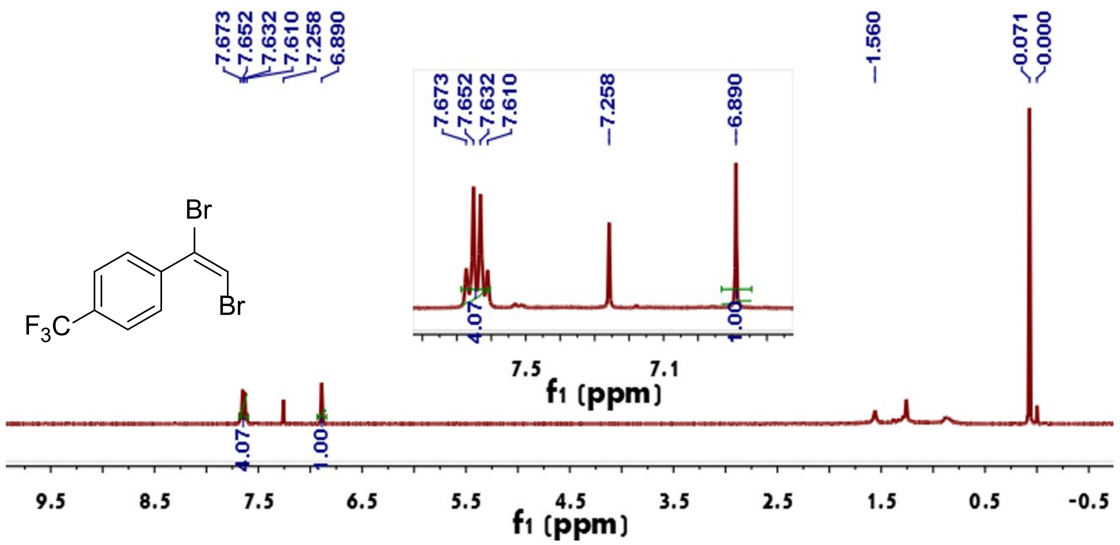

^{13}C{^{1}H} NMR (100 MHz, CDCl$_3$) spectrum of **3i**

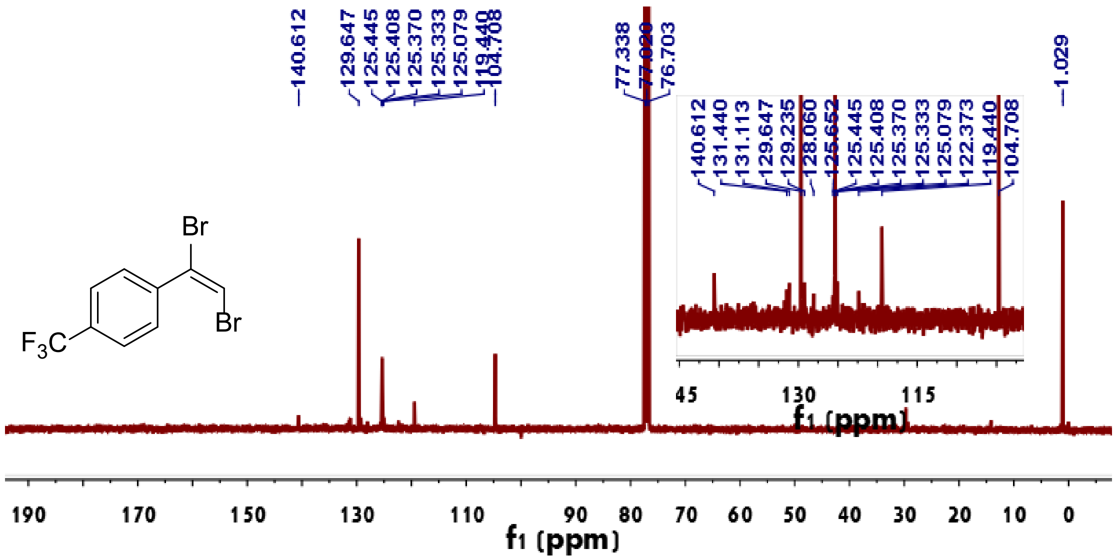

NMR Spectra of (*Z*)-1-(1,2-dibromovinyl)-4-(trifluoromethyl)benzene (4i):

^{1}H NMR (400 MHz, $CDCl_3$) spectrum of **4i**

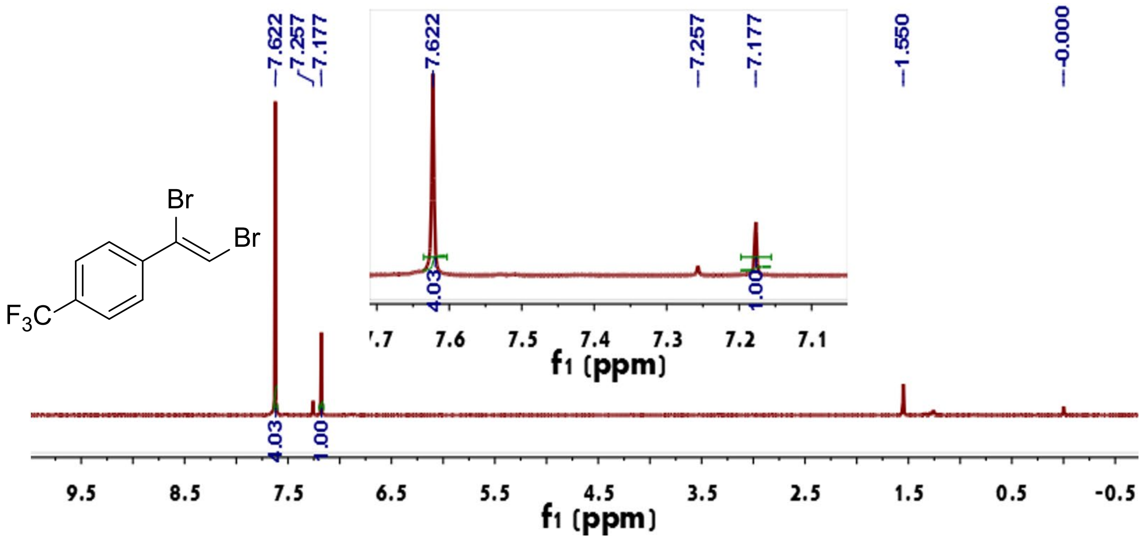

^{13}C{^{1}H} NMR (100 MHz, $CDCl_3$) spectrum of **4i**

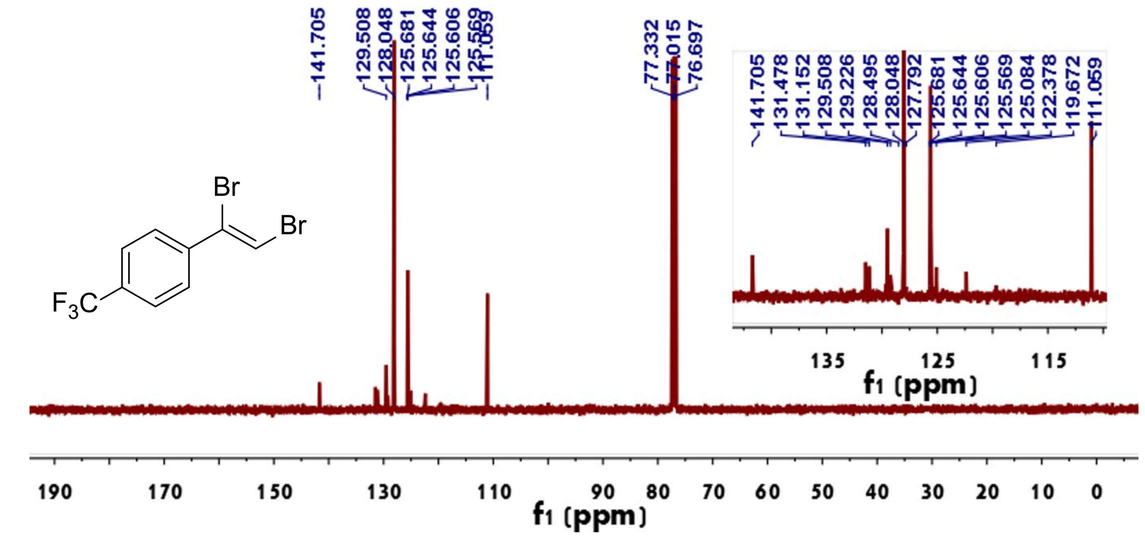

NMR Spectra of 2,2-dibromo-1-(4-(trifluoromethyl)phenyl)ethanone (5i):

^{1}H NMR (400 MHz, $CDCl_3$) spectrum of **5i**

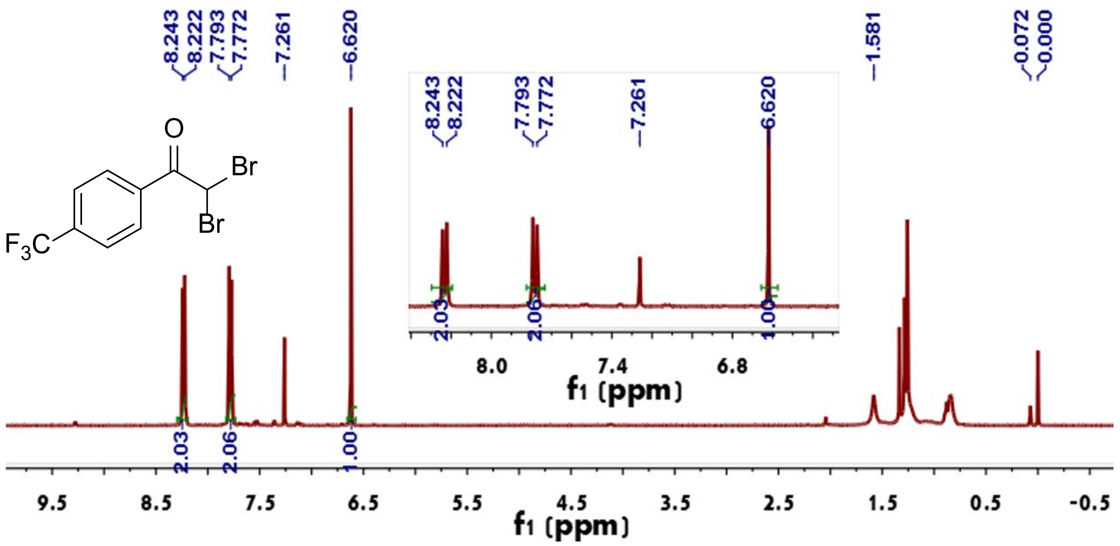

^{13}C{^{1}H} NMR (100 MHz, $CDCl_3$) spectrum of **5i**

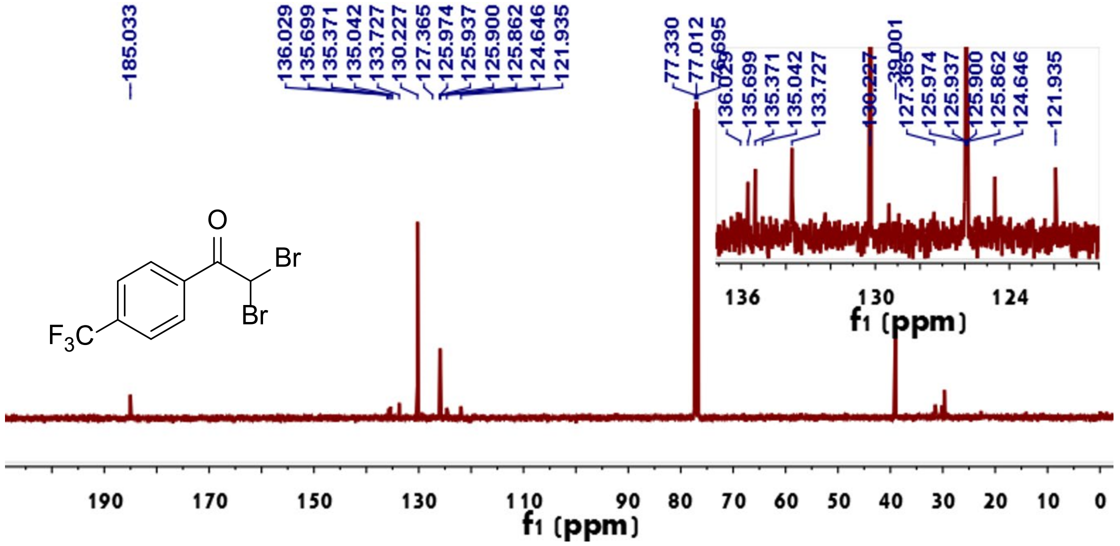

NMR Spectra of 1-(1,1,2,2-tetrabromoethyl)-4-(trifluoromethyl)benzene (6i):

^{1}H NMR (400 MHz, $CDCl_3$) spectrum of **6i**

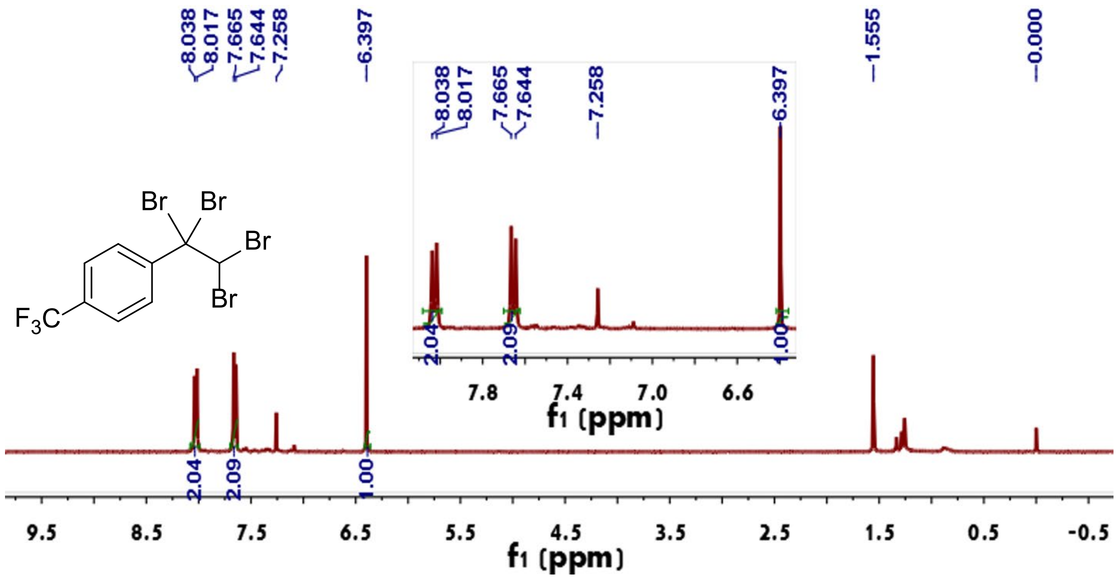

^{13}C{^{1}H} NMR (100 MHz, $CDCl_3$) spectrum of **6i**

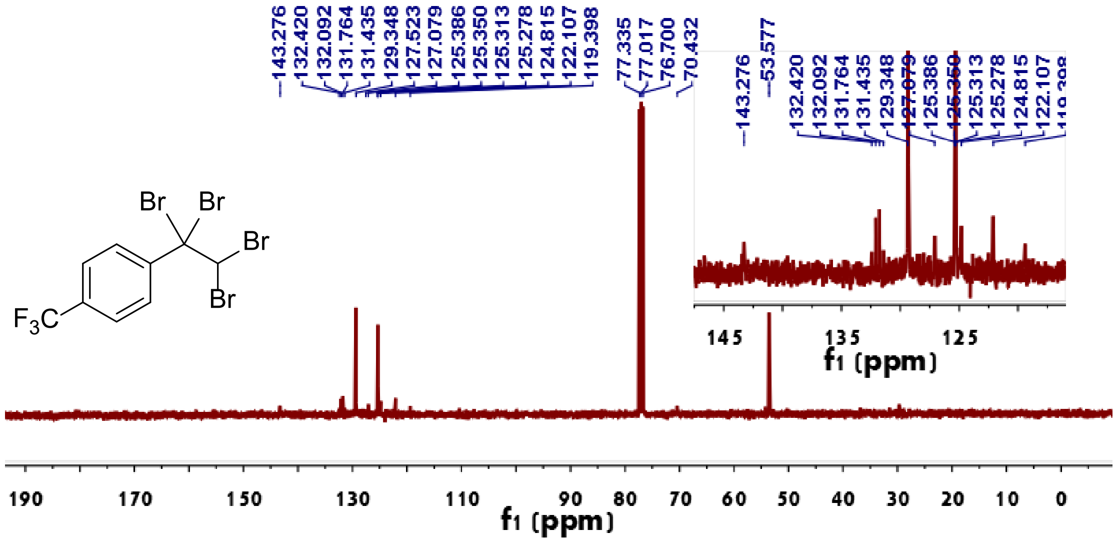

NMR Spectra of 1-(bromoethynyl)-4-nitrobenzene (2j):

^{1}H NMR (400 MHz, $CDCl_3$) spectrum of **2j**

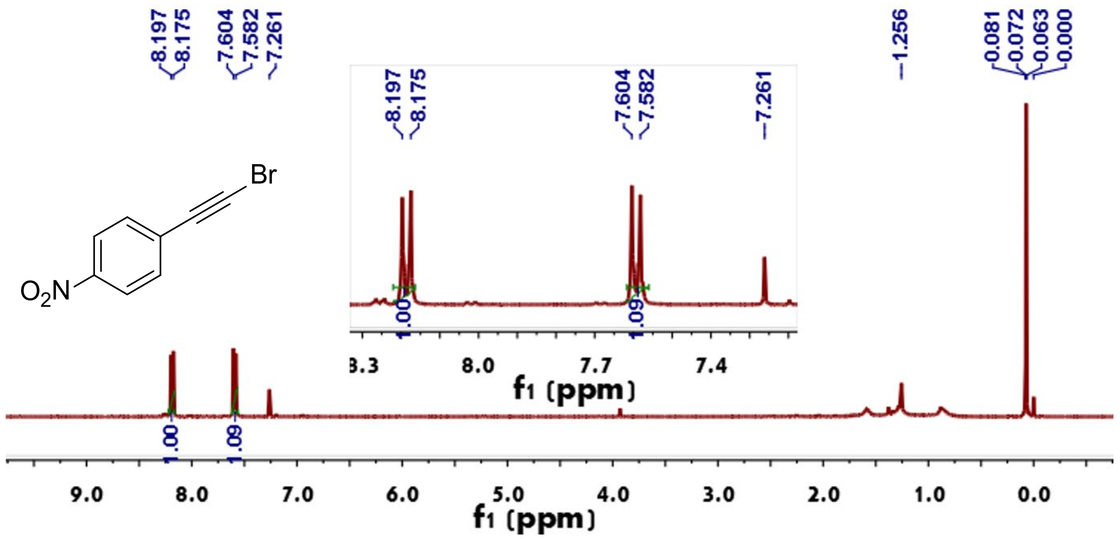

^{13}C{^{1}H} NMR (100 MHz, $CDCl_3$) spectrum of **2j**

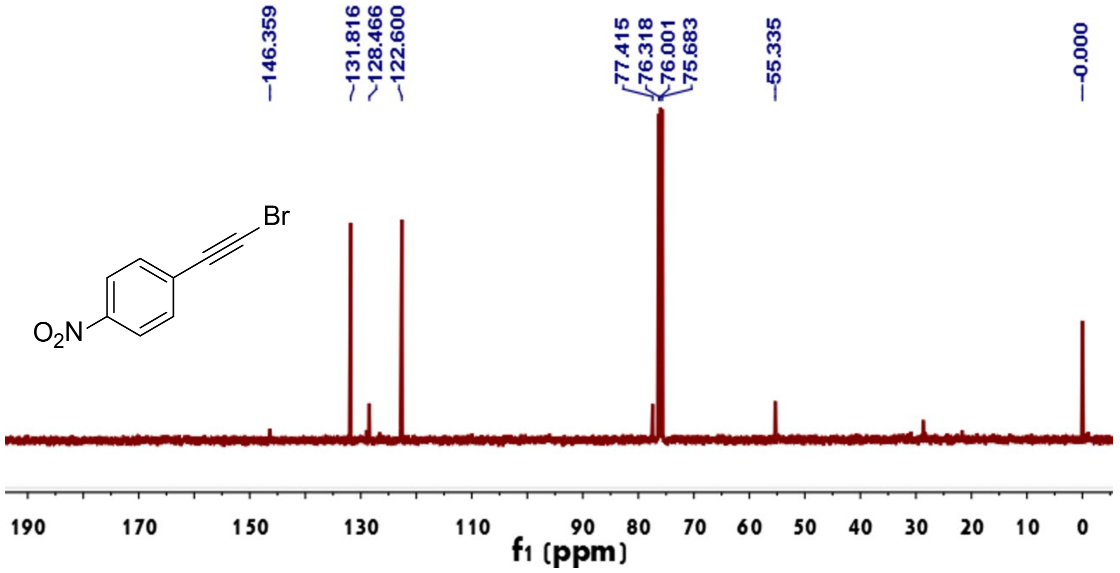

NMR Spectra of (*E*)-1-(1,2-dibromovinyl)-4-nitrobenzene (3j):

^{1}H NMR (400 MHz, $CDCl_3$) spectrum of **3j**

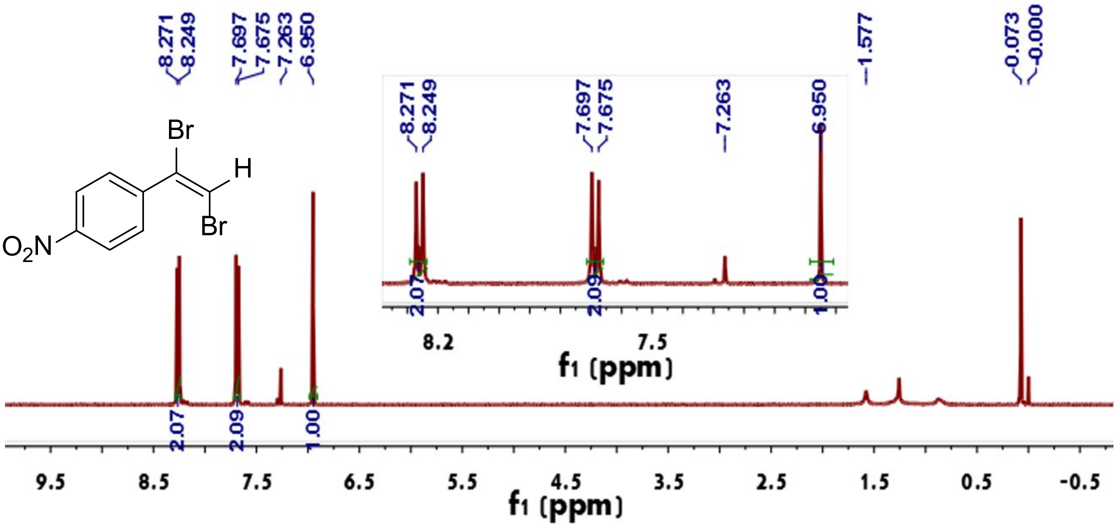

$^{13}C\{^1H\}$ NMR (100 MHz, $CDCl_3$) spectrum of **3j**

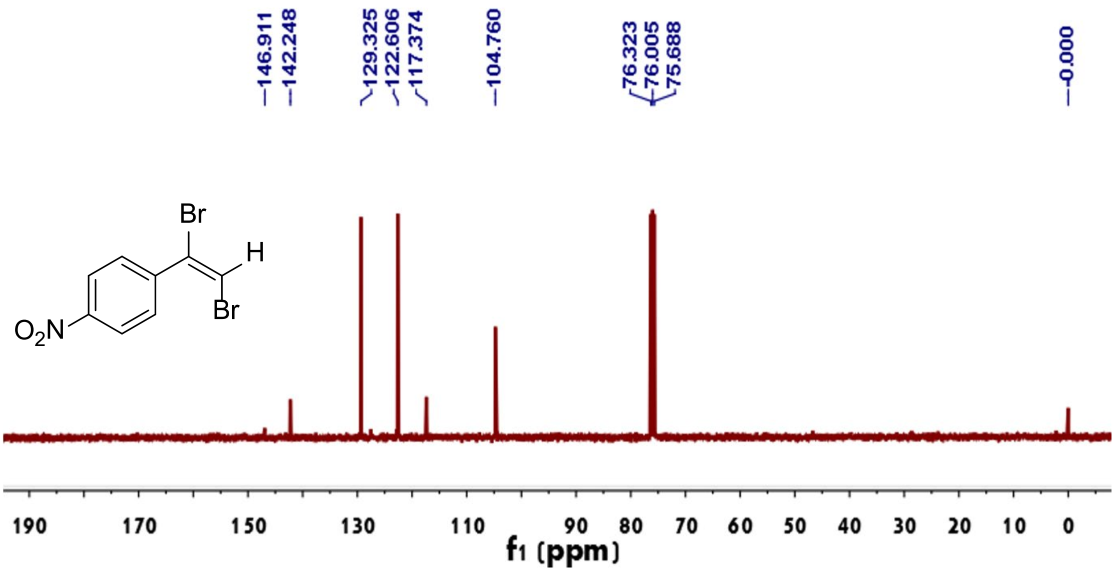

NMR Spectra of (*Z*)-1-(1,2-dibromovinyl)-4-nitrobenzene (4j):

^{1}H NMR (400 MHz, $CDCl_3$) spectrum of **4j**

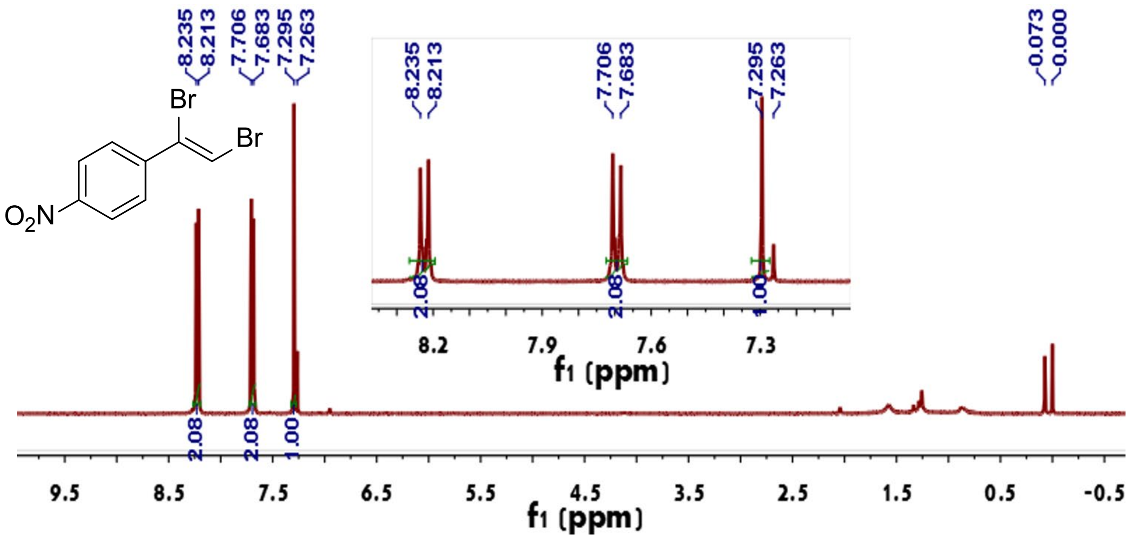

^{13}C{^{1}H} NMR (100 MHz, $CDCl_3$) spectrum of **4j**

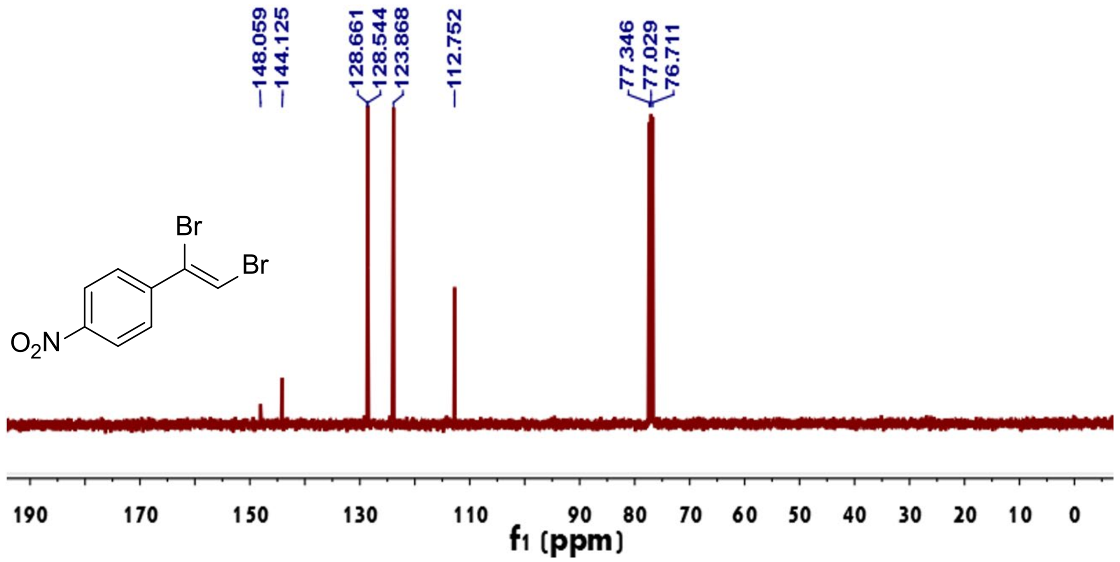

NMR Spectra of 2,2-dibromo-1-(4-nitrophenyl)ethanone (5j):

^{1}H NMR (400 MHz, $CDCl_3$) spectrum of **5j**

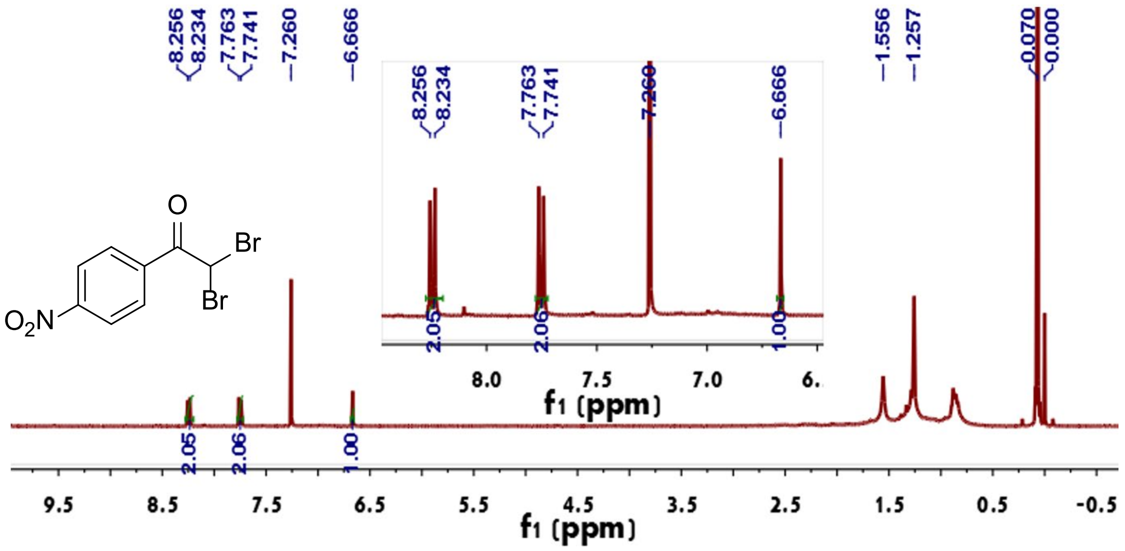

^{13}C{^{1}H} NMR (100 MHz, $CDCl_3$) spectrum of **5j**

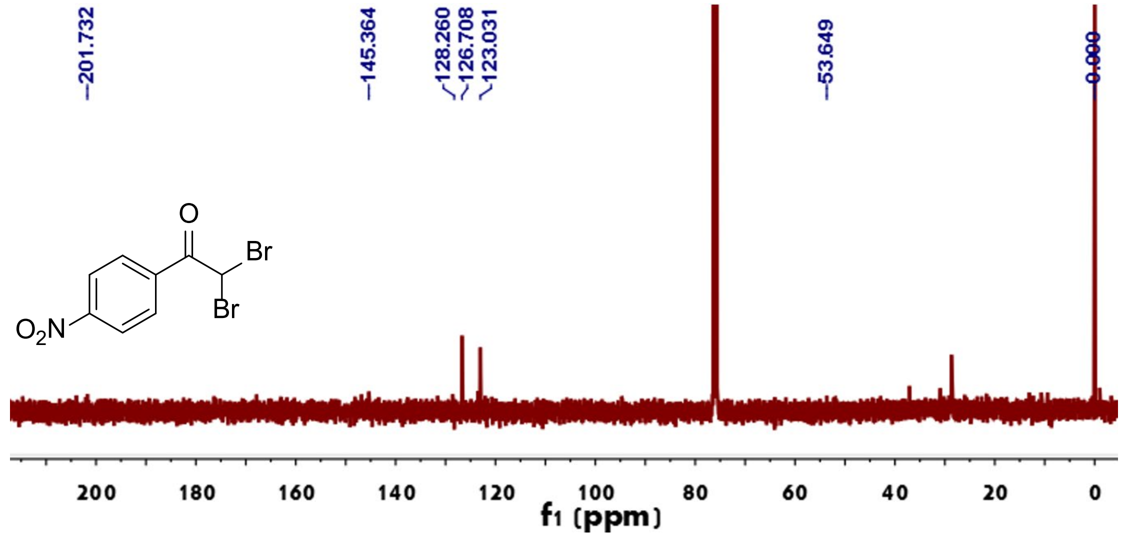

NMR Spectra of 1-nitro-4-(1,1,2,2-tetrabromoethyl)benzene (6j):

^{1}H NMR (400 MHz, $CDCl_3$) spectrum of **6j**

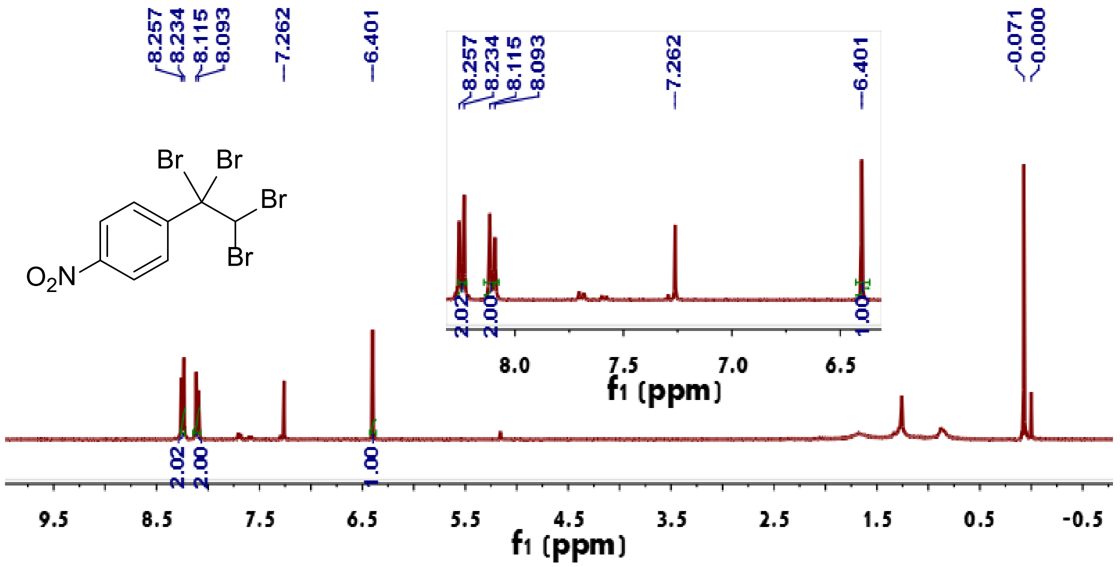

$^{13}C\{^{1}H\}$ NMR (100 MHz, $CDCl_3$) spectrum of **6j**

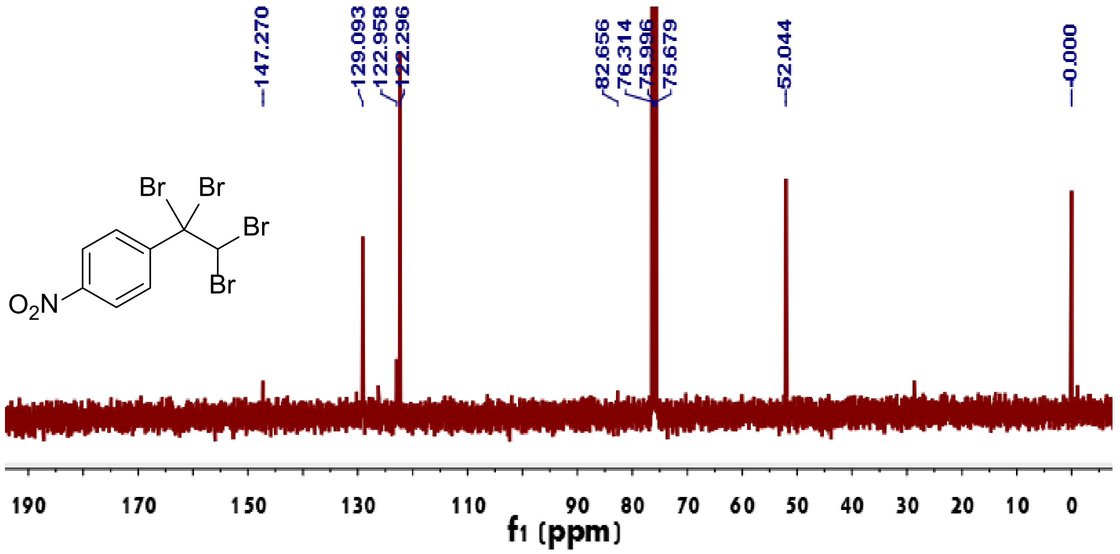

2D NMR Spectra of 3a and 4a

NOESY (400 MHz, $CDCl_3$) spectrum of ***(E)*-1-(1,2-dibromovinyl)-4-methylbenzene** (**3a**):

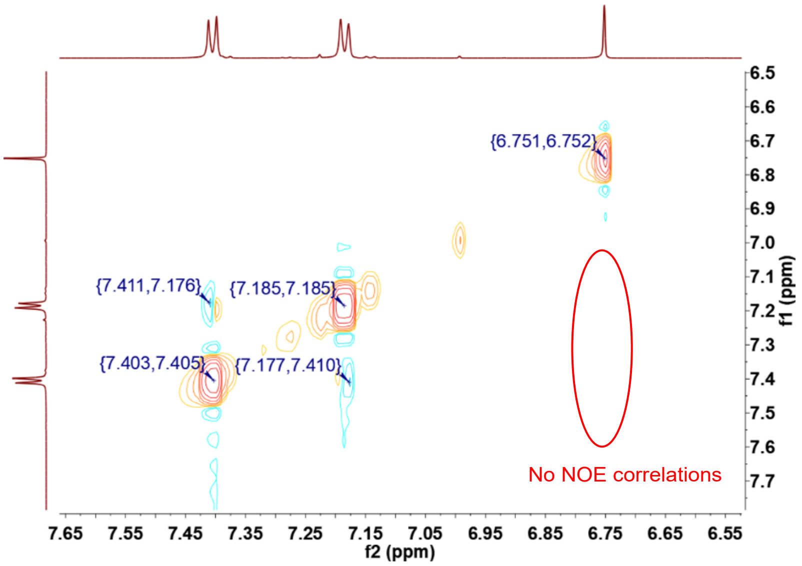

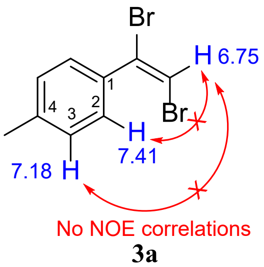

3a

NOESY (400 MHz, $CDCl_3$) spectrum of **(Z)-1-(1,2-dibromovinyl)-4-methylbenzene (4a)**:

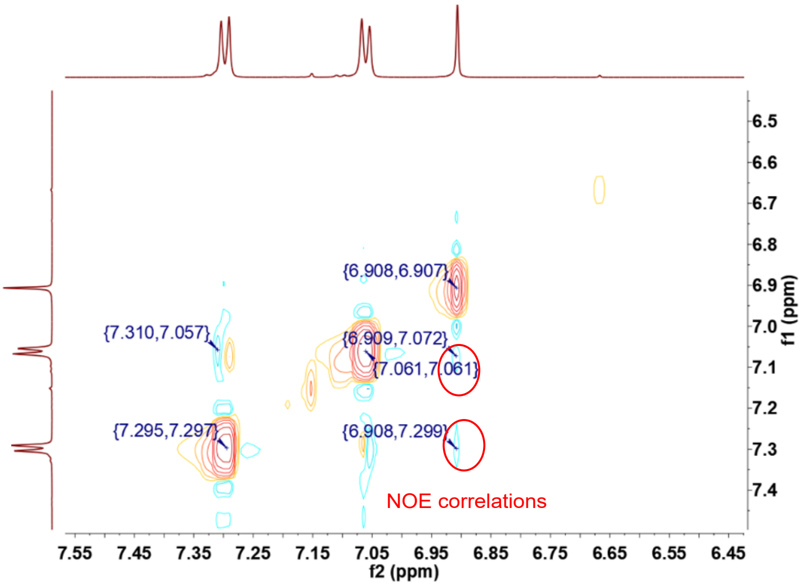

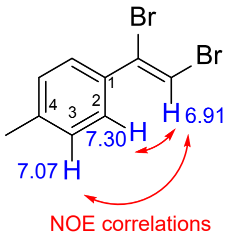

4a

The determination of (E/Z)-alkene by using 2D NMR. NOE correlations are observed between (***Z***)-H_{vinyl} (6.91 ppm) and H_2 (7.30 ppm), and between(***Z***)-H_{vinyl} (6.91 ppm) and H_3 (7.07 ppm) from the 2D NMR spectrum of **4a**. However, NOE correlation could not be able to observe between (***E***)-H_{vinyl} (6.75 ppm) and H_2 (7.41 ppm), or between (***E***)-H_{vinyl} (6.75 ppm) and H_3 (7.18 ppm) from the 2D NMR spectrum of **3a**.
